# PETISCO is a novel protein complex required for 21U RNA biogenesis and embryonic viability

**DOI:** 10.1101/gad.322446.118

**Published:** 2019-07-01

**Authors:** Ricardo J. Cordeiro Rodrigues, António Miguel de Jesus Domingues, Svenja Hellmann, Sabrina Dietz, Bruno F.M. de Albuquerque, Christian Renz, Helle D. Ulrich, Peter Sarkies, Falk Butter, René F. Ketting

**Affiliations:** 1 Biology of Non-coding RNA Group, Institute of Molecular Biology, 55128 Mainz, Germany;; 2 International PhD Programme on Gene Regulation, Epigenetics, and Genome Stability, 55128 Mainz, Germany;; 3 Quantitative Proteomics Group, Institute of Molecular Biology, 55128 Mainz, Germany;; 4 Graduate Program in Areas of Basic and Applied Biology, University of Porto, 4099-003 Porto, Portugal;; 5 Maintenance of Genome Stability Group, Institute of Molecular Biology, 55128 Mainz, Germany;; 6 Medical Research Council London Institute of Medical Sciences, London W12 0NN, United Kingdom;; 7 Institute of Clinical Sciences, Imperial College London, London W12 0NN, United Kingdom

**Keywords:** 21U RNA, *C. elegans*, IFE-3, MID domain, PID-1, PID-3, PRG-1, TOFU-6, enhancer of rudimentary, piRNA

## Abstract

Piwi proteins are important for germ cell development in most animals. These proteins are guided to specific targets by small guide RNAs, referred to as piRNAs or 21U RNAs in *Caenorhabditis elegans*. In this organism, even though genetic screens have uncovered 21U RNA biogenesis factors, little is known about how these factors interact or what they do. Based on the previously identified 21U biogenesis factor PID-1 (piRNA-induced silencing-defective 1), we here define a novel protein complex, PETISCO (PID-3, ERH-2, TOFU-6, and IFE-3 small RNA complex), that is required for 21U RNA biogenesis. PETISCO contains both potential 5′ cap and 5′ phosphate RNA-binding domains and interacts with capped 21U precursor RNA. We resolved the architecture of PETISCO and revealed a second function for PETISCO in embryonic development. This essential function of PETISCO is mediated not by PID-1 but by the novel protein TOST-1 (twenty-one U pathway antagonist). In contrast, TOST-1 is not essential for 21U RNA biogenesis. Both PID-1 and TOST-1 interact directly with ERH-2 using a conserved sequence motif. Finally, our data suggest a role for TOST-1:PETISCO in SL1 homeostasis in the early embryo. Our work describes a key complex for 21U RNA processing in *C. elegans* and strengthens the view that 21U RNA biogenesis is built on an snRNA-related pathway.

## Abstract (precis)

In this study, Cordeiro Rodrigues et al. identified a novel protein complex, PETISCO, acting in 21U RNA production in *C. elegans*, providing new mechanistic insights into small RNA biogenesis. They also identified a second essential function for PETISCO in embryonic development mediated by TOST-1.

Germ cells in many animals depend critically on a small RNA-driven pathway known as the piRNA pathway (Ketting 2011; Weick and Miska 2014). This pathway is characterized by members of the Piwi protein family that, bound to their small RNA cofactors (piRNAs), act in gene regulatory and transposon silencing pathways (Luteijn and Ketting 2013; Czech and Hannon 2016). In the absence of these pathways, germ cells do not form properly, ultimately leading to sterility. While transposons represent major targets for these pathways, it is also clear that nontransposon targets are functionally relevant for these pathways (Rojas-Ríos and Simonelig 2018). Many mechanistic aspects of the Piwi pathway are deeply conserved, such as enzymes involved in biogenesis, such as Zucchini, HEN1, and PNLDC1 or the closely related enzyme PARN (Gainetdinov et al. 2018; Lewis et al. 2018). Also, in most animals, piRNA precursors are long transcripts that are processed into multiple piRNA species. Nevertheless, many differences can be found as well. In *Drosophila*, for example, a piRNA amplification loop driven by two Piwi paralogs is coupled to the activity of a third nuclear Piwi protein that drives transcriptional silencing (Luteijn and Ketting 2013; Czech and Hannon 2016), whereas, in silk moths, a nuclear branch seems to be absent (Sakakibara and Siomi 2018). In mice, the piRNA amplification loop involves only one Piwi protein, Mili, but does drive the nuclear accumulation of another Piwi protein, Miwi2 (Czech and Hannon 2016). Nematodes represent a very interesting phylum with regard to piRNA pathways, as many species seem to have lost this pathway completely (Sarkies et al. 2015). *Caenorhabditis elegans* does have a functional piRNA pathway, but, compared with other animals, it is rather diverged.

The main *C. elegans* Piwi protein is named PRG-1 (*piwi*-related gene 1), and the small RNA cofactors bound by PRG-1 are known as 21U RNAs (Batista et al. 2008; Das et al. 2008; Wang and Reinke 2008). This name stems from the fact that these small RNAs are 21 nucleotides (nt) long and have a strong bias for a 5′ uracil. Target RNA recognition by PRG-1:21U complexes does not depend on full-length base-pairing between the 21U RNA and the target RNA (Bagijn et al. 2012; Lee et al. 2012) but follows microRNA (miRNA) target recognition rules (Seth et al. 2018; Shen et al. 2018; Zhang et al. 2018). This results in a broad target spectrum for PRG-1:21U RNA complexes, including genes whose expression in the germline is required. Both sequence-based Argonaute CSR-1 small RNA-based mechanisms have been proposed to counteract the silencing by PRG-1 on such targets (Seth et al. 2013; Frøkjaer-Jensen et al. 2016; Shen et al. 2018; Zhang et al. 2018). How the PRG-1 and CSR-1 pathways are molecularly connected is currently unknown. Target recognition by PRG-1 results in the recruitment of RNA-dependent RNA polymerase activity, which drives the synthesis of so-called 22G RNAs (Batista et al. 2008; Das et al. 2008; Shen et al. 2018). These 22G RNAs, named after their predominant 22-nt length and 5′ G bias, are bound by argonaute proteins that are specific for nematodes (also referred to as WAGO proteins) that ultimately drive the silencing of the 21U target. However, the majority of 22G RNAs, including the 22G RNAs bound by CSR-1, are not dependent on PRG-1, as other Argonaute proteins, such as ERGO-1, are also strongly driving 22G RNA biogenesis (Almeida et al. 2019), and 22G RNAs are also able to self-maintain (Ashe et al. 2012; Luteijn et al. 2012; Shirayama et al. 2012).

In the absence of PRG-1, the germline deteriorates over generations, eventually leading to sterility (Simon et al. 2014). The defects that accumulate are not genetic, since the phenotype can be reverted by, for instance, starvation (Simon et al. 2014). These observations have led to the suggestion that *prg-1* mutants accumulate a form of stress that ultimately leads to germ cell dysfunction (Heestand et al. 2018). More acute fertility defects can be observed in *prg-1* mutants when the 22G RNA biogenesis machinery is reactivated in zygotes after being defective in both of the parents. In this case, maternally provided PRG-1:21U complexes are required to prevent immediate sterility by preventing accumulation of 22G RNA populations that inappropriately silence genes that should be expressed (de Albuquerque et al. 2015; Phillips et al. 2015). This demonstrates that PRG-1 and its bound 21U RNA have a critical function in maintaining a properly tuned 22G RNA population in the germ cells. Nevertheless, the impact of PRG-1 on transposon silencing is rather modest, as, in *prg-1* mutants, only activation of the Tc3 transposon has thus far been demonstrated (Das et al. 2008).

Interestingly, the silencing of a 21U target can become independent of 21U RNAs themselves (Ashe et al. 2012; Luteijn et al. 2012; Shirayama et al. 2012). In this state, which has been named RNAe (RNAi-induced epigenetic silencing), the silencing has been completely taken over by a self-sustaining 22G RNA response and can be extremely stable. This includes a nuclear component that changes the histone methylation status of the targeted transgene, driven by the nuclear Argonaute protein HRDE-1 (Ashe et al. 2012; Buckley et al. 2012; Luteijn et al. 2012; Shirayama et al. 2012). Possibly, such an RNAe state may explain why transposons are not more broadly up-regulated in *prg-1* mutants because the Tc1 transposon is strongly activated in *prg-1;hrde-1* double mutants (de Albuquerque et al. 2015).

Given the important function of 21U RNAs driving a potentially very powerful silencing response, the biogenesis of 21U RNAs is a critical step. The large majority of genes encoding 21U RNAs are found in two main clusters on chromosome IV. Strikingly, each 21U RNA gene appears to encode a single 21U RNA and is characterized by a very specific sequence motif in the promoter that defines the 5′ end of the mature 21U RNA (Ruby et al. 2006). Transcription of these genes requires a protein named PRDE-1 and the transcription factor SNPC-4 (Kasper et al. 2014; Weick et al. 2014; Weng et al. 2018), the latter of which is also known to be involved in transcription of other short structural RNAs, such as snRNAs and splice leader RNAs (Kasper et al. 2014). An evolutionary analysis of 21U RNA loci in diverse nematodes has revealed that 21U loci may have evolved from snRNA loci (Beltran et al. 2019). These loci include both the strongly conserved U1 and U2 loci as well as loci producing so-called splice leader RNAs (SL1 and SL2) that are *trans*-spliced to the 5′ ends of a large fraction of all mRNAs in *C. elegans* (Blumenthal 2012). Strikingly, fragments of SL1 and snRNAs have been found in immunoprecipitated PRG-1, albeit at low levels (Gu et al. 2012), suggesting that such RNA molecules indeed have aspects in common with 21U RNA precursor molecules and that other aspects of the 21U RNA pathway also may have mechanistic links to snRNA biogenesis.

The 21U RNA precursor transcripts are between 23 and 30 nt and capped (Gu et al. 2012). The available data suggest the following order of steps in the maturation of the 21U precursor transcripts into mature 21U RNAs (Ruby et al. 2006; Gu et al. 2012; de Albuquerque et al. 2014; Goh et al. 2014; Weick et al. 2014). First, the precursors are processed at the 5′ end, resulting in decapping and removal of two nucleotides. The enzymes involved have not yet been identified. This step is followed by loading of the 5′ processed precursor into PRG-1 and trimming of the 3′ end by the 3′–5′ exonuclease PARN-1 (Tang et al. 2016). Finally, the 3′ end is 2′-O-methylated by HENN-1 (Billi et al. 2012; Kamminga et al. 2012; Montgomery et al. 2012). Little is known about other proteins acting at these 21U maturation steps even though a number of genes have been implicated in this process (Cecere et al. 2012; de Albuquerque et al. 2014; Goh et al. 2014; Kasper et al. 2014; Weick et al. 2014).

Here, we follow up on our previous identification of PID-1 (piRNA-induced silencing-defective 1) as a protein essential for 21U RNA production (de Albuquerque et al. 2014). Mutants lacking PID-1 produce very low amounts of mature 21U RNAs, and it was suggested that PID-1 acts at some step in 21U precursor processing. Other factors potentially acting at this step of 21U biogenesis (TOFU-1, TOFU-2, TOFU-6, and TOFU-7) were identified in a genome-wide RNAi screen (Goh et al. 2014). How these factors interconnect, however, remained unclear. Using immunoprecipitation and label-free quantitative mass spectrometry (IP-MS), we found that PID-1 interacts with two proteins that were identified by Goh et al. (2014): TOFU-6 and the unnamed protein Y23H5A.3, referred to here as PID-3. In addition, we identified two strongly conserved proteins interacting with PID-1: IFE-3, a *C. elegans* eIF4E homolog, and ERH-2, one of the *C. elegans* homologs of “enhancer of rudimentary.” Enhancer of rudimentary homologs are evolutionarily very well conserved proteins, with homologs present from plants to humans. Its mechanistic role is not very well established, but in *Schizosaccharomyces pombe*, ERH1 drives the decay of meiotic transcripts and interacts with the nuclear exosome and the nuclear CCR4–NOT complex (Sugiyama et al. 2016). Since we always found PID-3, ERH-2, TOFU-6, and IFE-3 together in a complex, we named it PETISCO (PID-3, ERH-2, TOFU-6, and IFE-3 small RNA complex). All PETISCO proteins are required for 21U biogenesis, and we show that PETISCO interacts with 21U RNA precursor molecules. Additionally, PETISCO mutants display a maternal effect lethal (Mel) phenotype, whereas *pid-1* and *prg-1* mutants are viable. We found that besides binding to PID-1, PETISCO can bind a protein, C35D10.13, with similarities to PID-1. We named this protein TOST-1 (twenty-one U pathway antagonist). Mutants for *tost-1* produce 21U RNAs, display enhanced 21U-driven silencing, and have a Mel phenotype. Both PID-1 and TOST-1 interact with ERH-2 using a conserved motif, strongly suggesting a mutually exclusive mode of binding to PETISCO. PID-1-bound PETISCO has a role in 21U RNA biogenesis, and the TOST-1-bound version functions in another pathway, which is essential for embryonic viability. Our data suggest that the latter might be linked to SL1 splice leader RNA homeostasis.

## Results

### Identification of PID-1 interactors

**Figure 1.**
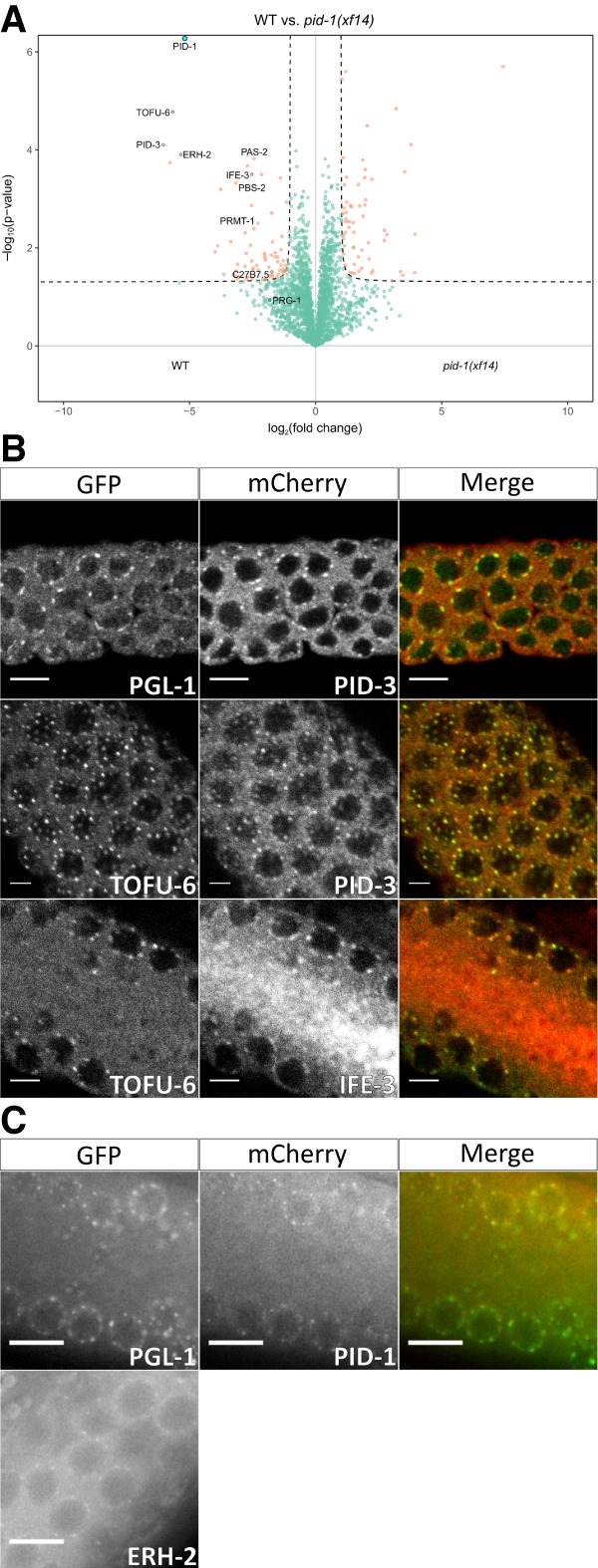
PID-1 interactors reside in P granules. (*A*) Volcano plot representing label-free proteomic quantification of PID-1 immunoprecipitations from nongravid adult extracts. Immunoprecipitations were performed and analyzed in quadruplicates. The *X-*axis represents the median fold enrichment of individual proteins in wild-type (WT) versus the *pid-1(xf14)* mutant strain. The *Y*-axis indicates −log_10_(*P*-value) of observed enrichments. Dashed lines represent thresholds at *P* = 0.05 and twofold enrichment. Blue data points represent values out of scale. Red and green data points represent values above and below threshold, respectively. (*B*,*C*) Expression pattern and localization of tagged PETISCO components and P-granule marker PGL-1. Endogenous promoters and 3′ untranslated regions were used for PETISCO proteins. Proteins and observable tags are indicated. (*B*) Immunostaining images were acquired with a laser scanning confocal microscope. (*C*) Live worm images were acquired under a wide-field fluorescent microscope. Scale bars, 5 µm. The contrast of the images has been enhanced.

To identify proteins interacting with the 21U biogenesis factor PID-1 (de Albuquerque et al. 2014), we performed immunoprecipitations with PID-1-specific antibodies followed by protein identification using mass spectrometry (IP-MS). As a control, we precipitated PID-1 from two independent *pid-1* loss-of-function strains. Both experiments identified a rather restricted set of proteins (Fig. 1A; Supplemental Fig. S1A). TOFU-6 and PID-3 were identified as the most prominent PID-1 interactors, and, for the latter, we further validated the interaction with PID-1 through immunoprecipitation-Western blotting (Supplemental Fig. S1B). Given that these two proteins were identified in an RNAi screen for 21U biogenesis factors (Goh et al. 2014), we considered these as functionally relevant PID-1 interactors. TOFU-6 is a protein with a Tudor domain, a potential eIF4E interaction motif, and an RRM domain, whereas PID-3 has an RRM domain and a MID domain (Supplemental Fig. S1C). Two other proteins were consistently identified as PID-1 interactors: IFE-3 and ERH-2 (Fig. 1A; Supplemental Fig. S1A). IFE-3 is one of the five *C. elegans* homologs of eIF4E. Previous work demonstrated that IFE-3 binds to the 7-methylguanylate (m7G) cap (Jankowska-Anyszka et al. 1998). Interestingly, 21U precursor transcripts appear to be not *trans*-spliced (Gu et al. 2012), implying that they have an m7G cap structure at their 5′ end. Hence, IFE-3 may bind to the 5′ cap structure of the 21U precursor transcripts. ERH-2 is one of the two *C. elegans* homologs of a protein known as “enhancer of rudimentary.” Homologs of this protein are strongly conserved from plants to mammals. Even though a protein structure for the human homolog has been described (Arai et al. 2005), its molecular function is still unclear. In Supplemental Figure S1C, we present a schematic of all of these PID-1 interactors with their identified domains.

We next probed the subcellular localization of PID-1 and its four identified interactors by expressing them from transgenes generated through the so-called miniMos approach (see the Materials and Methods; Frøkjaer-Jensen et al. 2014). For each gene, we used its own promoter and 3′ untranslated region (UTR) sequences (Supplemental Table S1). All transgenes were able to rescue mutant phenotypes (Supplemental Figs. S2, S3A), indicating that the expressed proteins are functional. All four interactors show a characteristic perinuclear punctate localization in the germline syncytium, overlapping partially, but not fully, with the P-granule marker PGL-1 (Fig. 1B,C). IFE-3 has a clear granular expression in the primordial germ cells in the embryo (Supplemental Fig. S3B), whereas the remaining interactors show a dispersed cytoplasmic distribution across the entire early embryo (Supplemental Fig. S3C,D). These results show that all of the identified PID-1 interactors are expressed concomitantly in early embryos, and the germline, where they are found in close proximity to P granules.

### PID-1 interactors mutually interact to form PETISCO

**Figure 2.**
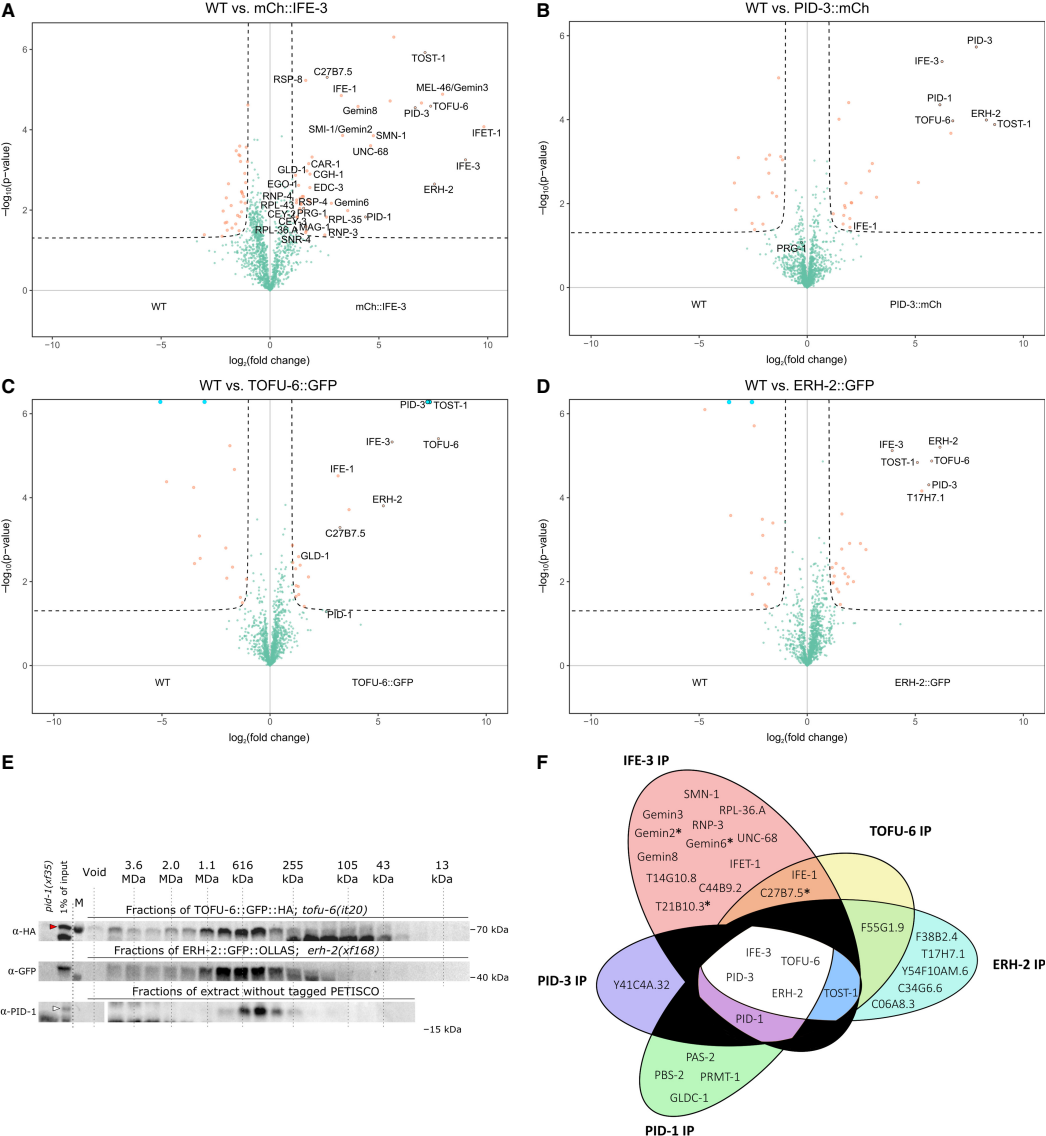
PID-1 interactors form a novel protein complex, PETISCO. (*A*–*D*) Volcano plot representing label-free proteomic quantification of quadruplicate 3xFlag::mCherry::IFE-3;*ife-3(xf101)* (*A*), PID-3::mCherry::Myc;*pid-3(tm2417)* (*B*), TOFU-6::GFP::HA;*tofu-6(it20)* (*C*), and ERH-2::GFP::OLLAS;*erh-2(xf168)* (*D*) immunoprecipitations from nongravid adult extracts. The *X*-axis represents the median fold enrichment of individual proteins in the control (wild-type [WT]) versus the transgenic strain. The *Y*-axis indicates −log_10_(*P*-value) of observed enrichments. Dashed lines represent thresholds at *P* = 0.05 and twofold enrichment. Blue data points represent values out of scale. Red and green data points represent above and below threshold, respectively. (*E*) Size exclusion chromatography of nongravid adult worm extracts of the indicated strains. Fractions were collected and probed for HA, GFP, and PID-1, respectively. The approximate molecular weights of the fractions are indicated. *pid-1(xf35)* extract was used as probing control. The red arrowhead indicates full-length TOFU-6::GFP, and the white arrowhead indicates PID-1. *pid-1(xf35)* extract was used as negative control for PID-1. (*F*) Venn diagram summarizing significant interactions in PETISCO protein immunoprecipitations. The asterisk represents protein found significantly enriched in only one experiment of 3xFlag::mCherry::IFE-3;*ife-3(xf101)* immunoprecipitation.

To probe to what extent the identified PID-1 interactors reciprocally interact and to what extent they in turn interact with additional proteins, we performed IP-MS on TOFU-6, IFE-3, PID-3, and ERH-2 in nongravid animals using the tagged proteins expressed from the above-described transgenes. Extracts from nontransgenic wild-type animals were used as negative controls. As shown in Figure 2, A–D, these experiments revealed that all four proteins coprecipitate with each other. In addition, we also found an uncharacterized protein (C35D10.13) that systematically coprecipitated with the PID-1 interactors but was absent from the PID-1 immunoprecipitations. This factor, which we named TOST-1, is further described below. RNase treatment did not disrupt PID-3 interactions (Supplemental Fig. S4A) and had very little effect on IFE-3 partners, resulting only in the loss of PID-1 (Supplemental Fig. S4B). PID-3 IP-MS in the absence of PID-1 demonstrated that all interactions were maintained (Supplemental Fig. S4C). Finally, in size exclusion chromatography, PID-1, TOFU-6, and ERH-2 displayed similar elution profiles, with an apparent molecular weight of ∼400 kDa (Fig. 2E). We summarize the network of PID-1 interactors in Figure 2F. Given the presented data, it is likely that they form a discrete complex. We named this complex PETISCO.

As mentioned before, IFE-3 is one of the *C. elegans* eIF4E homologs. Surprisingly, in our experiments, we did not detect any of the known translation initiation factors to be associated with IFE-3. The IFE-3-expressing transgene does rescue the *ife-3* mutant phenotype (Supplemental Fig. S2A,B), implying that IFE-3 may not play a role in initiating translation. We did detect a number of additional proteins bound to IFE-3 (Fig. 2A) that provide clues to IFE-3 function. One such protein is IFET-1, a homolog of human EIF4E nuclear import factor 1 and a negative regulator of translation (Sengupta et al. 2013). We further consistently detected many components of the SMN complex (Fig. 2A; Cauchi 2010). The SMN complex plays a major role in snRNP biogenesis, implicating IFE-3 in this process as well.

### PETISCO architecture

**Figure 3.**
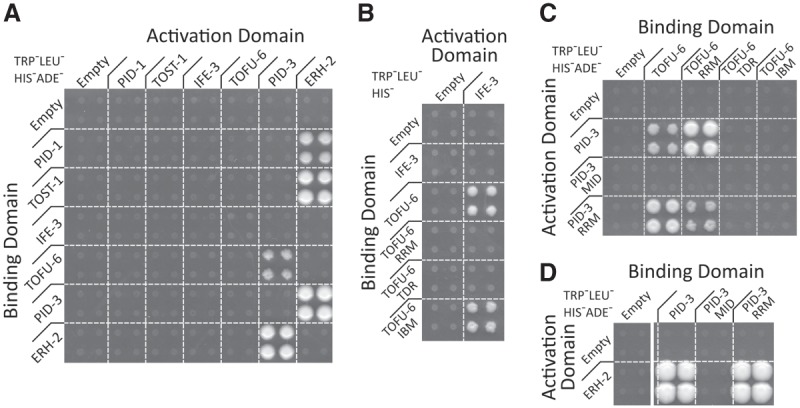
PETISCO architecture. (*A*–*D*) Y2H interaction assays of PETISCO subunits in low-stringency (TRP^−^LEU^−^HIS^−^) or high-stringency (TRP^−^LEU^−^HIS^−^ADE^−^) medium as indicated. Interactions were screened in both Y2H orientations. (*A*) Full-length proteins. (*B*) TOFU-6 and individual domains tested for interaction with full-length IFE-3. (*C*) Interactions between PID-3 and TOFU-6. (*D*) Interaction between PID-3 and ERH-2. For details on domains and other selection conditions, see Supplemental Figures S1C and S5.

Having established the components of PETISCO, we next dissected the molecular interactions within this complex. Using the yeast two-hybrid (Y2H) system, we scored interactions between each possible pair of individual PETISCO subunits (Fig. 3A; Supplemental Fig. S5A). This resulted in the following interactions that are most likely direct: IFE-3 interacts only with TOFU-6. TOFU-6 in turn interacts with PID-3, which in turn also interacts with ERH-2. Finally, both PID-1 and TOST-1 were found to interact only with ERH-2. Self-interaction was detected for ERH-2, although under lower-stringency conditions, indicating that ERH-2 may be present as a dimer in PETISCO.

We also investigated which domains of the PETISCO subunits are responsible for the protein interactions using the same Y2H setup. The putative eIF4E interaction motif (Mader et al. 1995; Grüner et al. 2016) within the C terminus of TOFU-6 is indeed responsible for the interaction between TOFU-6 and IFE-3 (Fig. 3B). No interactions were found for the Tudor domain of TOFU-6. The RRM domain of TOFU-6 was found to bind to the RRM domain of PID-3 (Fig. 3C), and the same RRM domain of PID-3 was found to also interact with ERH-2 (Fig. 3D). Lower-stringency and control selections of the same experiments are shown in Supplemental Fig. S5, B–D. This Y2H setup did not allow us to determine whether the PID-3 RRM domain can sustain both the TOFU-6 and the ERH-2 interactions simultaneously; however, the fact that we found PID-3, TOFU-6, and ERH-2 strongly enriched in the immunoprecipitations of one another suggests that it can.

### PETISCO subunits are required for 21U RNA biogenesis

**Figure 4.**
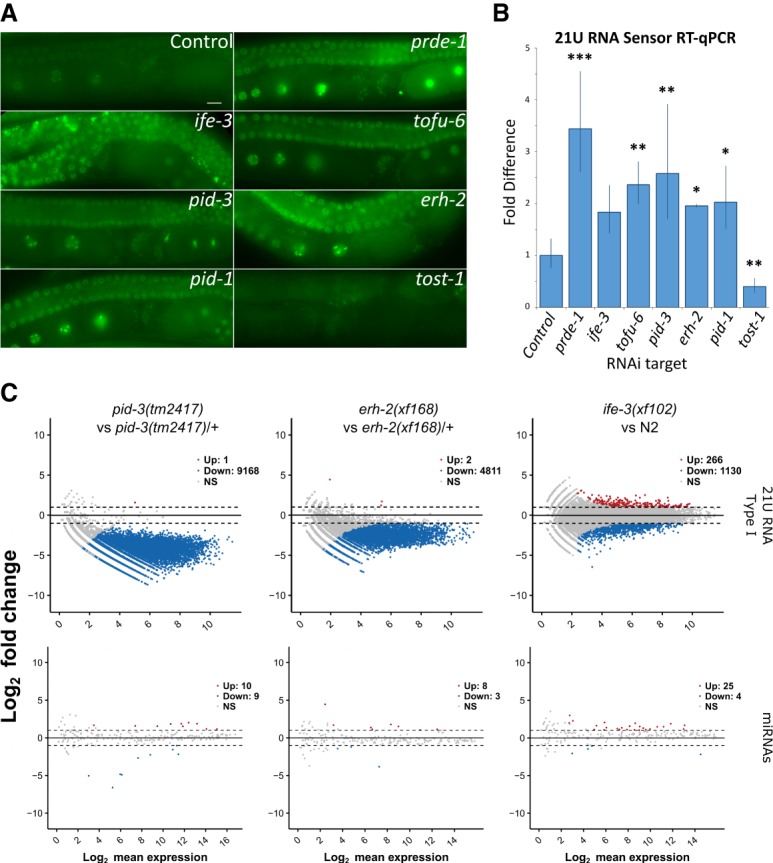
PETISCO is required for 21U RNA biogenesis. (*A*) Wide-field fluorescent microscopy of adult hermaphrodites carrying the GFPH2B-21U sensor transgene in a sensitized background. Worms were subjected to RNAi via feeding (targets are indicated) from L1 larval stage to adulthood. Empty RNAi vector served as a negative control. Scale bar, 10 µm. (*B*) Quantitative RT-PCR of the 21U sensor transgene in the adult worm populations of *A*. Values were obtained from experimental triplicates and technical duplicates, normalized to *pmp-3* mRNA levels. The significance of sample versus control was tested with Dunnett's post hoc test. (***) *P*-value < 0.001; (**) *P*-value < 0.01; (*) *P*-value < 0.05. Error bars represent standard deviation. (*C*) Pairwise differential gene expression of type I 21U RNAs and miRNAs in *pid-3(tm2417)*, *erh-2(xf168)*, and *ife-3(xf102)* versus the respective controls. Samples consisted of nongravid adult individuals. Red and blue dots indicate up-regulated and down-regulated transcripts, respectively.

Since at least two components of PETISCO (PID-1 and TOFU-6) play a major role in 21U RNA biogenesis, we hypothesized that the other components are also part of this pathway. We first tested whether they affect the silencing of a GFP sensor construct that reports on the activity of the 21U pathway (21U sensor) (Bagijn et al. 2012). We used a strain that contains this sensor plus a *pid-1(xf14)* mutation that is rescued by transgenic expression of PID-1. In this strain, the sensor is silenced (Fig. 4A,B), although not completely. This partial 21U sensor silencing brings two advantages: First, the 21U sensor is still activated by loss of 21U RNAs. Second, partial silencing allows for scoring of both increased and decreased sensor activity to be detected.

Animals from this sensitized sensor strain were subjected to RNAi targeting the various PETISCO subunits, and sensor activity was evaluated by microscopy and RT-qPCR (Fig. 4A,B). As expected, RNAi against *pid-1* and *prde-1* activated the sensor. Likewise, RNAi against *ife-3*, *pid-3*, *tofu-6*, and *erh-2* activated the sensor. These data show that PETISCO subunits, like the PETISCO interactor PID-1, are required for a fully functional 21U RNA silencing pathway.

To extend the above observation, we sequenced small RNAs from mutants. To enrich for 21URNAs, we did not pretreat RNA with 5′ pyrophosphatase, preventing analysis of the highly abundant 22G RNAs. We created mutant alleles using CRISPR–Cas9 for each of the PETISCO subunit genes. We were able to derive deletion alleles for *pid-3*, *erh-2*, and *ife-3* (Supplemental Fig. S6A; Supplemental Table S2) and isolated and sequenced small RNAs. We did not make mutants for *tofu-6*, since RNAi on *tofu-6* was already shown to significantly reduce 21U RNA production (Goh et al. 2014). All experiments were done in triplicates and normalized to total library depth. Following this normalization, replicates correlate very well (Supplemental Table S3). First, miRNAs were found to be largely unaffected (Fig. 4C; Supplemental Fig. S6B). Next, we analyzed 21U RNA abundance and found that all tested PETISCO subunits significantly affected 21U RNA abundance (Fig. 4C; Supplemental Fig. S6C). The effect of *ife-3(xf102)* mutation on 21U RNA accumulation is less pronounced (Fig. 4C), but, overall, 21U RNA levels are still significantly reduced (Supplemental Fig. S6C). Redundancy between IFE-3 and other eIF4E homologs could be a reason for this less pronounced effect on 21U RNA levels, as we found IFE-1, a nonselective TMG/m7G binder (Jankowska-Anyszka et al. 1998), enriched in some of our IP-MS experiments (Fig. 2A–C; Supplemental Fig. S4B). As described previously for *pid-1* mutants (de Albuquerque et al. 2014), the 21U RNA species that are left in PETISCO mutants have the normal bias for U at position 1 and a size of 21 nt (Supplemental Fig. S6D).

The so-called type II 21U RNAs, which come from loci lacking the canonical Ruby motif (Gu et al. 2012), are only mildly affected by loss of PETISCO components (Supplemental Fig. S6E). The 26G RNAs are similarly weakly expressed and surprisingly also appear to be mildly affected by loss of PID-3, ERH-2, or IFE-3 (Supplemental Fig. S6E). This finding may reflect a currently unknown mechanistic link between 26G RNA and 21U RNA biogenesis but may also result from indirect effects.

### PETISCO subunits are essential for embryogenesis

Besides the defects in 21U biogenesis, the PETISCO mutants also display a so-called Mel phenotype: Homozygous mutant offspring from a heterozygous animal develop into fertile adults, but the embryos display a fully penetrant arrest before gastrulation and never hatch (Supplemental Fig. S2C; Supplemental Table S4). This phenotype has already been described for *tofu-6*, which is also known as *mel-47* (Minasaki et al. 2009). The *ife-3* mutant displays a mixed phenotype, as described previously (Keiper et al. 2000): Homozygous mutant offspring from a heterozygous animal develop into adults that can display either a masculinized germline (Mog) or a Mel phenotype. Interestingly, we note that *pid-1* mutants also display a Mog phenotype, albeit at low frequency (Supplemental Table S4; Supplemental Fig. S7).

### PID-1 and TOST-1 define distinct functions of PETISCO

The fact that PETISCO subunits are essential for embryogenesis is in contrast to the observation that loss of 21U RNAs through other means, such as loss of PRG-1 or PID-1, does not result in a Mel phenotype. Interestingly, RNAi knockdown of *tost-1* resulted in embryonic lethality. In our IP-MS experiments, we consistently found this protein to interact with the PETISCO subunits (Fig. 2A–D) yet not with PID-1 (Fig. 1A; Supplemental Fig. S1A). Furthermore, *tost-1(rnai)* displays enhanced rather than disrupted silencing of the sensitized 21U sensor (Fig. 4A,B). This phenotype led us to name this protein TOST-1.

**Figure 5.**
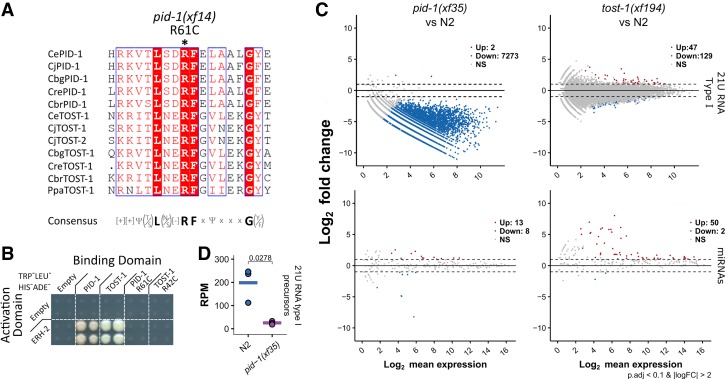
PID-1 and TOST-1 define different PETISCO functions. (*A*) Alignment of a short region of PID-1 and TOST-1 homologs from different nematodes. A conserved arginine residue was found to be mutated in *pid-1(xf14)*, as indicated. A consensus sequence is presented *below* the alignment. Alignment was performed with Muscle version 3.8 (Edgar 2004), and representation was performed with ESPrit version 3.0 (Robert and Gouet 2014). (*B*) Y2H interaction assay of PID-1, TOST-1, and PID-1/TOST-1 carrying the corresponding arginine mutation found in pid-1(xf14). High-stringency plates (TRP^−^LEU^−^HIS^−^ADE^−^) were used. For other conditions, see Supplemental Figure S4D. (*C*) Global levels of type I 21U RNA precursors in wild-type (N2) and *pid-1(xf35)* nongravid adults. Values were obtained from CIP-RppH-treated smRNA libraries. (RPM) Reads per million mapped reads. Individual data points of three independent replicates are shown, and the horizontal bar represents the mean. Significance was tested with Student's *t*-test, and *P*-values are indicated in the graph. (*D*) Pairwise differential gene expression of type I 21U RNAs and miRNAs in *pid-1(xf35)* and *tost-1(xf194)* versus wild-type (N2) control. Samples consisted of gravid adult individuals. Red and blue dots indicate up-regulated and down-regulated transcripts, respectively.

We tested both PID-1 and TOST-1 for interactions with PETISCO subunits. We found that both PID-1 and TOST-1 specifically interact only with ERH-2 (Fig. 3A). While the overall amino acid sequences of PID-1 and TOST-1 do not display convincing homology (Supplemental Fig. S8A), when aligned with PID-1 and TOST-1 homologs from other nematode species, a conserved motif emerges {[_+_][_+_]_Ψ(T/S)_**L**_(N/S)_[_−_]**RF**_xΨxxx_**G**_(Y/F)_} (Fig. 5A). Strikingly, the *xf14* allele that we identified for *pid-1* in our previously described genetic screen caries a mutation of the fully conserved arginine residue within this motif (R61C) (de Albuquerque et al. 2014). When introduced into the Y2H experiment, PID-1(R61C) did not interact with ERH-2, and the analogous mutation in TOST-1 also disrupted its ERH-2 interaction (Fig. 5B). These data strongly suggest that both PID-1 and TOST-1 share a conserved short motif required for ERH-2 interaction.

To further understand TOST-1 function, we generated loss-of-function alleles for *tost-1* using CRISPR–Cas9 (Supplemental Fig. S6A). Loss of TOST-1 did not significantly affect 21U RNA biogenesis (Fig. 5C; Supplemental Fig. S8B) but did result in a fully penetrant Mel phenotype (Supplemental Table S4). Among the alleles that we generated, we found one allele, *tost-1(xf196)*, with a 58-bp deletion that removes the splice acceptor site of its third exon (Supplemental Fig. S6A), resulting in either a frameshift or a C-terminal truncation. *tost-1(xf196)* displays a temperature-sensitive (TS) effect: At 25°C, the animals are fully Mel, while, at 15°C, the animals are viable. The fact that this allele is viable at 15°C suggests that the N-terminal part of TOST-1 is critical. The interaction motif that we identified is still intact in TOST-1(xf196)*,* consistent with the idea that it is essential for TOST-1 function. This TS allele allowed us to probe the Mel phenotype in more detail through temperature shift experiments (Supplemental Fig. S8C). First, animals grown at the restrictive temperature were able to produce viable offspring after shifting them to the permissive temperature. This shows that there are no structural or developmental defects in the germline of these animals that prevent them from producing live offspring. Second, the TS allele allows us to probe when TOST-1 function is required. The time required for animals shifted to the permissive temperature to produce viable embryos (8 h) is markedly longer than the time it takes from fertilization to egg-laying (∼200 min at 15°C), implying that the embryos laid after 8 h were fertilized and raised at the permissive temperature. Despite functional TOST-1 in these embryos, they still arrest; thus, TOST-1 activity in the germline is essential for embryonic development of the subsequent generation. Nevertheless, TOST-1 function is still required in early embryos because, when animals are shifted from permissive to restrictive temperature, the first arrested embryos are laid already after 2 h. This is very close to the time of residency within the uterus (∼150 min at 25°C), and hence these embryos had been fertilized very close to the time of the temperature shift in a gonad that still had functional TOST-1 (Supplemental Fig. S3C). Small RNA sequencing from animals shifted to nonpermissive temperatures revealed no significant defects in 21U RNA populations (Supplemental Fig. S8D).

In conclusion, both TOST-1 and PID-1 bind to the same PETISCO subunit, and loss of these two proteins results in different phenotypes. The combined phenotypes of both *pid-1* and *tost-1* mutants is found in mutants for the other PETISCO subunits, strongly suggesting that PID-1 and TOST-1 define two distinct aspects of PETISCO function.

### PID-1 is required for 21U precursor accumulation

To gain insight into a potential role for PID-1—and TOST-1 by analogy—in PETISCO, we asked whether 21U precursor sequences are affected by loss of PID-1. Following dephosphorylation and decapping, we cloned and sequenced small RNA from wild-type and *pid-1(xf35)* mutants in triplicate. This revealed a significant reduction of 21U precursors in *pid-1* mutants (Fig. 5D), as measured by reads from 21U loci that start at position −2 with respect to the mature 5′ end >22 nt (Gu et al. 2012). This indicates that PID-1 increases the stability of 21U precursor RNA molecules. Such stabilization may occur through binding to PETISCO.

### PETISCO interacts with 21U precursor RNA

**Figure 6.**
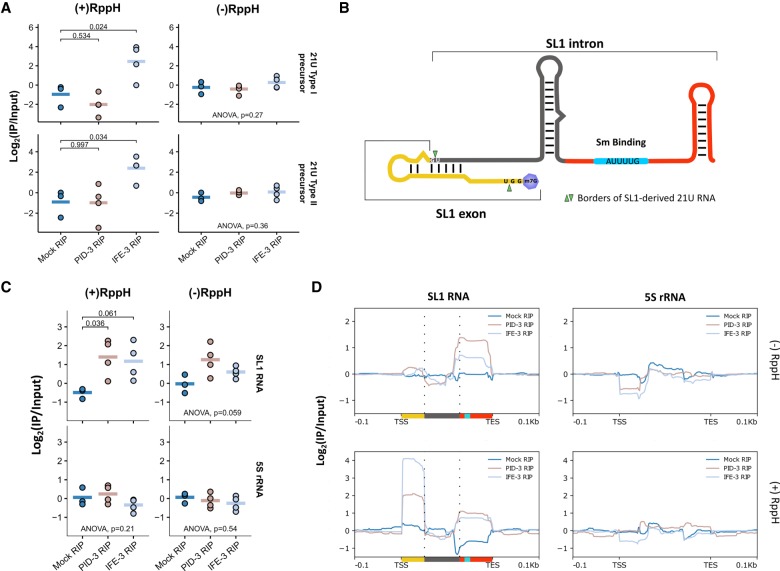
PETISCO interacts with 21U precursors and with SL1 snRNA. (*A*) Fold enrichments of 21U RNA precursors in mock (N2), 3xFlag::mCherry::IFE-3;*ife-3(xf101)*, and PID-3::mCherry::Myc;*pid-3(tm2417)* RIPs over paired inputs in nongravid adult worms. The *left* column displays RppH-treated samples, and the *right* column shows nontreated samples. Individual data points of three or more independent replicates are shown, and the horizontal bar represents the mean. The significance of sample versus mock RIP was tested with Dunnett's post hoc test, and *P*-values are indicated in the graph. (*B*) Schematic representation of the SL1 RNA. Green arrows represent sequence borders of the previously SL1-derived 21U RNA. (*C*) Fold enrichments of SL1 RNA and 5S rRNA in mock (N2), 3xFlag::mCherry::IFE-3;*ife-3(xf101)*, and PID-3::mCherry::Myc;*pid-3(tm2417)* RIPs over paired inputs in nongravid adult worms. The *left* column displays RppH-treated samples, and the *right* column shows nontreated samples. Individual data points of three or more independent replicates are shown, and the horizontal bar represents the mean. The significance of sample versus mock RIP was tested with Dunnett's post hoc test, and *P*-values are indicated in the graph. (*D*) Coverage profile, normalized to paired input of SL1 of the data displayed in *C*. Colors *below* SL1 RNA correspond to scaled colors represented in *B*.

To test whether PETISCO subunits interact with 21U precursor RNA, we performed RIP-seq (RNA immunoprecipitation [RIP] combined with deep sequencing) experiments on IFE-3 and PID-3, including RppH treatment to remove 5′ caps before cloning, and checked the abundance of precursor molecules, as defined above. While PID-3 immunoprecipitations did not show enrichment, we detected significant enrichment of 21U RNA precursors in immunoprecipitations for IFE-3 only after decapping (Fig. 6A), indicating that the PETISCO subunit IFE-3 interacts with capped 21U precursor molecules.

### PETISCO interacts with SL1 RNA

We noticed a striking accumulation of a 3′ fragment of SL1 RNA in a number of the mutants that we tested (Fig. 6B; Supplemental Fig. S9A,B). Such accumulation was less pronounced or absent for another small noncoding RNA that is produced from the same gene cluster as SL1: 5S rRNA (Fig. 6B,C). Importantly, this enrichment of SL1 fragments was very clear in *tost-1* mutants but not in *pid-1* mutants. Also, *ife-3* and *erh-2*, but not *pid-3*, mutants displayed a similar accumulation (Fig. 6C). For SL2, we observed the enrichment only in *ife-3* mutant libraries (Supplemental Fig. S8B) but point out that SL2 read counts are comparatively very low. U-snRNA-derived fragments were only mildly affected in these mutants (Supplemental Fig. S9B).

We next probed whether SL1 or SL1 fragments may be bound by PETISCO using the RIP data as described above. We detected strong and significant enrichment of SL1 and, to a lesser extent, SL2 sequences in both PID-3 and IFE-3 immunoprecipitations (Fig. 6C; Supplemental Fig. S10A). In contrast, 5S rRNA and U-snRNAs are not enriched or are even depleted (Fig. 6C; Supplemental Fig. S10A). The read coverage over SL1 and SL2 shows that two fragments are detected: one at the 5′ end and another at the 3′ end (Fig. 6D; Supplemental Fig. S10B). The 5′ fragment is capped, as this fragment is absent from libraries from non-RppH-treated RNA (Fig. 6D). Interestingly, this 5′ fragment fits the expected precursor molecule for the previously reported SL1-derived 21U RNA (Fig. 6B; Gu et al. 2012). We conclude that PETISCO binds to SL1 RNA and that loss of PETISCO leads to an accumulation of SL1 RNA fragments.

### Evolutionary analysis of PETISCO

**Figure 7.**
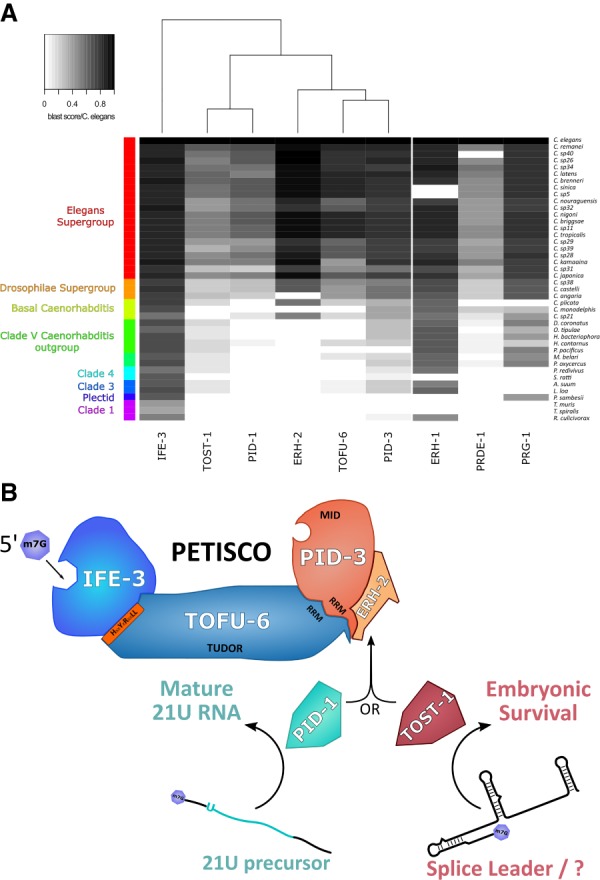
Schematic representation of PETISCO function and conservation in nematodes. (*A*) Heat map representing the conservation of PETISCO orthologs in nematodes according to BLASTp score versus *C. elegans*. ERH-1, PRDE-1, and PRG-1 are included for comparison. (*B*) A schematic of the proposed PETISCO function displaying its dual function: one in 21U biogenesis and another needed for embryonic survival. The latter may be connected to splice leader homeostasis. The m7G capped transcripts interact with the IFE-3. The presence of a 5′P-binding activity in PID-3 may reflect 5′ end processing of transcripts bound by PETISCO. The nuclease responsible has not yet been identified. ERH-2 serves as an anchor for PID-1 or TOST-1 driving PETISCO function toward either 21U RNA biogenesis or its embryonic survival function, respectively.

Many nematodes outside the *Elegans* and *Drosophilae* supergroups have lost PRG-1 (Sarkies et al. 2015). We therefore probed the conservation of PETISCO subunits among nematode species (Fig. 7A), also including PRG-1, PRDE-1, and ERH-1. ERH-1 is a close paralog of ERH-2, more closely related to Erh proteins in other organisms such as *Schizosaccharomyces pombe* or humans. The result shows that the PETISCO subunits IFE-3, PID-3, TOST-1, and, to a lesser extent, TOFU-6 are widely conserved, including in species lacking PRG-1, such as *Ascaris suum* and *Loa loa*. We hypothesize that the function of these factors in these species may relate to the Mel phenotype displayed by the respective mutants in *C. elegans*. PID-1 conservation is restricted to species that have PRG-1. This is consistent with the idea that PID-1 may be specialized to 21U RNA biogenesis. Nevertheless, many species with PRG-1 do not have PID-1 but do have TOST-1, suggesting that the separation of function between PID-1 and TOST-1 is not absolute. We note that, indeed, in *tost-1* mutants, 21U RNAs seem to be slightly affected even if not statistically significantly (Supplemental Fig. S8B). Finally, ERH-1 is more strongly conserved than ERH-2, with ERH-2 generally being absent in species in which PID-1 is not detected. Possibly, the ERH-2:PID-1 combination represents a specialization of PETISCO to 21U RNA biogenesis in the *Elegans* and *Drosophilae* supergroups.

## Discussion

We identified a protein complex, PETISCO, that is involved in at least two different pathways. PID-1:PETISCO acts in 21U RNA biogenesis, whereas TOST-1:PETISCO is essential for embryogenesis (Fig. 7B). Here we discuss various aspects of this complex and present hypotheses for the different potential functions of PETISCO.

### PETISCO as a 21U precursor processing platform

We found that PETISCO contains two proteins with predicted 5′ end interaction domains: a 5′ cap-binding activity in IFE-3 and a potential 5′P-binding domain, the MID domain (Schirle and MacRae 2012), in PID-3. We also found that 5′ unprocessed 21U precursors are bound by IFE-3 but are not found in PID-3 immunoprecipitations. We suggest that following binding of a capped 21U RNA precursor to IFE-3, the precursor loses its two 5′ nucleotides, including the cap structure, relatively quickly. Given that we did not detect the capped precursors in PID-3 immunoprecipitations, PID-3 may join the complex only after 5′ processing in order to stabilize the resulting 5′P-carrying intermediate. The RRM domains present in both PID-3 and TOFU-6 may further help to stabilize precursor binding even if they are also involved in protein–protein interaction (Maris et al. 2005). This may then allow for handing over the 21U intermediate to PRG-1 followed by further 3′ end trimming and 2′O-methylation. TOFU-6 has an additional Tudor domain, which may be involved in keeping PETISCO within the observed perinuclear granules, providing the proper subcellular environment for PETISCO function. However, detailed functional analysis of the individual domains will require further studies, most notably in vitro analyses with purified components. Finally, the nuclease responsible for 5′ end processing still remains elusive, since none of the PETISCO members presents obvious homology with a nuclease.

### Two strongly conserved proteins in PETISCO: ERH-2 and IFE-3

Two subunits of PETISCO are very well conserved evolutionarily: ERH-2 and IFE-3. IFE-3 has been shown to bind m7G cap structures and bind much less efficiently to the typical TMG cap structures found on the mature SL1 snRNA and hence on the majority of mRNAs in *C. elegans* (Jankowska-Anyszka et al. 1998). This activity of IFE-3 likely allows the observed interaction with 21U precursor RNAs. It is not immediately clear, though, why *ife-3* mutants display only a weak defect in 21U RNA biogenesis, but this could be due to functional redundancy with IFE-1, which we regularly detected in our IP-MS experiments. IFE-3 was also found to interact with many members of the SMN complex. SMN complex proteins are not found in immunoprecipitations of any of the other PETISCO subunits, so the IFE-3–SMN interaction is likely to be physically separated from PETISCO. The SMN complex is well known for its involvement in snRNP assembly (Cauchi 2010) and, in *C. elegans*, has been shown to be required for SL1 *trans*-splicing (Philippe et al. 2017). Interestingly, a homolog for Gemin5, the m7G cap-binding protein in the human SMN complex (Xu et al. 2016), is not found in *C. elegans*. Possibly, IFE-3 could fulfill this function in *C. elegans*. We note that, like loss of IFE-3 (Keiper et al. 2000), loss of the *C. elegans* Gemin3 homolog MEL-46 (Minasaki et al. 2009) and the U2 snRNP-associated factor MOG-2 (Zanetti et al. 2011) also results in Mel and Mog phenotypes, strengthening the link between IFE-3 and snRNP homeostasis. Possibly, this dual function of IFE-3 leads to the sporadic production of the so-called type II 21U RNAs, such as the 21U RNA derived from SL1 and those derived from other transcripts carrying an m7G cap (Gu et al. 2012).

ERH-2 is one of the two “enhancer of rudimentary” proteins of *C. elegans*. The other homolog, ERH-1, is more closely related to Erh-related proteins in other species and also is more broadly conserved in nematodes. However, ERH-1 was not found in any of our mass spectrometry analyses. This suggests that ERH-2 is specific for PETISCO and, given that ERH-2 conservation closely follows PID-1 (Fig. 7A), may be a specialization with regard to 21U RNA biogenesis. In *S. pombe*, Erh1 forms a tight complex with a protein named Mmi, and together they are involved in nuclear mRNA degradation of meiotic transcripts involving proteins such as ARS2 and the CCR4–NOT complex (Sugiyama et al. 2016). Hence, Erh1 in *S. pombe* appears to act as a bridge between an RNA-binding protein (Mmi) and an RNA processing machinery. Our data suggest that ERH-2 in *C. elegans* may have a similar function in that it connects PETISCO to a 21U and/or a SL1 precursor processing enzyme. The size of PETISCO, as determined by size exclusion chromatography (∼400 kDa) (Fig. 2E), suggests that the complex might contain multiple copies of its subunits. In this light, it is interesting to note that purified human and *S. pombe* Erh proteins are dimeric (Arai et al. 2005; Xie et al. 2019) and that we found self-association of ERH-2 in our Y2H analysis (Supplemental Fig. S5A).

### PID-1 and TOST-1: two handles for two different PETISCO functionalities?

We show that PETISCO has at least two functions: 21U RNA biogenesis and another essential function in embryogenesis. The latter may be related to SL1/2 snRNP homeostasis but may also relate to other yet unidentified RNA species. Independent of what this additional function may be, an image arises in which the 21U RNA pathway may have evolved out of an already existing small noncoding RNA network that became linked to an Argonaute-driven gene silencing program.

We show that the different functionalities of PETISCO can be separated through two different “adapter” proteins (PID-1 and TOST-1) that bind to PETISCO via ERH-2. The overall sequence similarity between PID-1 and TOST-1 is very low. Nevertheless, we identified a motif that is required for binding to ERH-2; hence, both proteins likely bind to PETISCO in a mutually exclusive way. Indeed, no TOST-1 was detected in PID-1 immunoprecipitations (Supplemental Fig. S1A), and also no PID-1 was detected in TOST-1 immunoprecipitations (Zeng et al. 2018). An IP-MS experiment on PID-3 in the absence of PID-1 (therefore enriching for TOST-1:PETISCO specifically) did not reveal much change compared with wild type (Supplemental Fig. S4C), suggesting that stable interactions within PID-1:PETISCO and TOST-1:PETISCO are very similar, if not identical.

What could the different functions of PETISCO be and how can these be steered by PID-1 and TOST-1? First, related to different PETISCO functions, this could entail differential processing of different substrate RNAs. We found SL1 transcripts as well as 21U RNA precursor transcripts bound by PETISCO, yet the former is not turned into 21U RNA effectively, while the latter is. This difference could be related to PID-1 versus TOST-1 association with PETISCO. Whether PID-1 and TOST-1 act in substrate selection and/or substrate processing remains an open question. We did find that in *pid-1* mutants, 21U precursor levels are lower than in wild type. This could hint that PID-1 plays a role in 21U precursor recruitment to PETISCO; however, other explanations for this result certainly also exist. To resolve the function of PID-1/TOST-1 and also the other subunits, PETISCO complexes will need to be made and studied in vitro. In addition, cross-linking before the IP-MS experiment may be able to trap more transient interactions between PETISCO and RNA processing enzymes, providing new leads to study PETISCO function in further detail. Finally, PID-1 was not found to be expressed in embryos, whereas other subunits, such as PID-3 and TOFU-6, are readily detectable in embryos. Hence, interaction studies on PETISCO at different developmental stages may also be a promising route for further studies.

## Materials and methods

### *C. elegans* genetics and culture

*C. elegans* strains were cultured according to standard laboratory conditions (Brenner 1974) unless stated otherwise. Animals for IP-MS were grown at 20°C in OP50 high-density plates (Schweinsberg and Grant 2013) for two generations, synchronized, and plated on standard plates for the generation before harvest, unless indicated otherwise. The Bristol N2 strain was used as a reference wild-type strain. A strain list and the procedures for genome editing, transgenesis, and RNAi are in the Supplemental Material.

### Microscopy

Wide-field fluorescence microscopy images were obtained using a Leica DM6000B, and confocal microscopy images were acquired with a Leica TCS SP5. Images were processed using Leica LAS software, ImageJ, and Adobe Photoshop. The protocol used for immunostaining is in the Supplemental Material.

### Y2H

Two-hybrid assays were performed in the haploid strain PJ69-4a and the pGAD and pGBD plasmid series as described previously (James et al. 1996). Cell pinning was performed with Rotor HAD (Singer Instruments, ROT-001).

### RNA isolation, qPCR, and library preparation

RNA was isolated by lysing worms with protK followed by TRIzol LS extraction. In RIP experiments, TRIzol LS was added directly to the immunoprecipitation beads after washing. Quantitative PCR was done in an Applied Biosystems ViiA7 real-time PCR system (Thermo Fisher Scientific) with cDNA made from 500 ng of total RNA using ProtoScript first strand cDNA synthesis kit (New England Biolabs, E6300) and Oligo d(T)_23_VN. Next-generation sequencing library preparation was performed with NEXTflex small RNA-seq kit version 3 following step A to step G of Bioo Scientific`s standard protocol (version 16.06). Further details on these protocols, including details on bioinformatic analysis of next-generation sequencing data, are in the Supplemental Material.

### Immunoprecipitations

Worm pellets were thawed on ice and mixed 1:1 with 2× lysis buffer (20 mM Tris.Cl, 300 mM NaCl, 1 mM EDTA, 1% [v/v] Igepal CO-630 at pH 7.5) with 2× protease inhibitors (cOmplete mini, EDTA-free; Roche, 11836170001). A Bioruptor Plus (Diagenode) sonicator was used to lyse worms (10 cycles of 30/30 sec at 4°C and high energy), and debris was removed by spinning. Lysate protein concentration was determined with Pierce BCA protein assay kit (Thermo Fisher Scientific, 23225). Lysates were diluted in 1× lysis buffer plus 1× protease inhibitors to a final concentration of 1.5 mg of protein per milliliter, and a total of 0.75 mg of protein was used per immunoprecipitation. At this step, input samples were collected into 2× NuPAGE LDS sample buffer (Life Technologies, NP0007) plus 200 mM DTT and boiled for 10 min. Anti-mCherry immunoprecipitations were performed with RFP-Trap_MA beads (Chromotek, rtma-20), and anti-GFP immunoprecipitations were performed with GFP-Trap_MA beads (Chromotek, gtma-20); in both cases, 25 µL of bead slurry was used, and samples were rotated for 2 h at 4°C. Subsequent washes were made with wash buffer (10 mM Tris.Cl, 150 mM NaCl, 0.5 mM EDTA at pH 7.5) plus protease inhibitors in accordance with Chromotek protocols. When appropriate, RNase treatment was done with 20 µL of RNase A/T1 mix per 1 mL of sample. Samples were rotated for 20 min at 4°C followed by the described immunoprecipitation protocol. For RIP-seq experiments, lysates were diluted in 1× lysis buffer + 1× protease inhibitors + 1× SUPERaseIn RNase inhibitor to a final concentration of 1.5 mg/mL protein, and a total of 2.1 mg of protein was used per immunoprecipitation. Further details are in the Supplemental Material.

### Mass spectrometry

Measurement was done on a Q Exactive Plus mass spectrometer (Thermo) operated with a Top10 data-dependent tandem mass spectrometry acquisition method per full scan (Bluhm et al. 2016). Measurements were processed with MaxQuant version 1.5.2.8 (Cox and Mann 2008) using the WormBase protein fasta database (version WS265) and standard settings except that LFQ quantitation and match between runs were activated. Further details are in the Supplemental Material.

## Supplementary Material

Supplemental Material
