# Supplementary material for: PETISCO is a novel protein complex required for 21U RNA biogenesis and embryonic viability
Source: Genes Dev. 2019 Jul 1;33(13-14):857–70. doi: 10.1101/gad.322446.118 (PMC6601512; doi:10.1101/gad.322446.118)
Supplement: Supplemental Material [file supp_33_13-14_857__index.html]

PETISCO is a novel protein complex required for 21U RNA biogenesis and embryonic viability — Supplemental Material 

# PETISCO is a novel protein complex required for 21U RNA biogenesis and embryonic viability

## Supplemental Material

- Supplemental\_Material.pdf
- Supplemental\_Table\_S2.xlsx
- Supplemental\_Source\_material.zip
- Supplemental\_Table\_S1.xlsx
