# Supplementary material for: PETISCO is a novel protein complex required for 21U RNA biogenesis and embryonic viability
Source: Genes Dev. 2019 Jul 1;33(13-14):857–70. doi: 10.1101/gad.322446.118 (PMC6601512; doi:10.1101/gad.322446.118)
Supplement: Supplemental Material [file supp_gad.322446.118_Supplemental_Material.pdf]

Supplemental information to:

PETISCO is a novel protein complex required for 21U RNA biogenesis and embryonic viability

Ricardo J. Cordeiro Rodrigues[1,2], António Miguel de Jesus Domingues[1], Svenja Hellmann[1], Sabrina Dietz[2,3], Bruno F. M. de Albuquerque[1,4], Christian Renz[5], Helle D. Ulrich[5], Peter Sarkies[6,7], Falk Butter[3], René F. Ketting[1]

Institute of Molecular Biology (IMB), Ackermannweg 4, 55128, Mainz, Germany

[1] IMB, Biology of Non-coding RNA Group

[2] International PhD Programme on Gene Regulation, Epigenetics & Genome Stability, Mainz, Germany

[3] IMB, Quantitative Proteomics Group

[4] Graduate Program in Areas of Basic and Applied Biology, University of Porto, 4099-003 Porto, Portugal.

[5] IMB, Maintenance of Genome Stability Group

[6] MRC London Institute of Medical Sciences, London W12 0NN, UK; Institute of Clinical Sciences, Imperial College London, London W12 0NN, UK.

[7] Institute of Clinical Sciences, Imperial College London, London W12 0NN, UK.

correspondence: r.ketting@imb.de

Running title:

PETISCO is required for 21U RNA biogenesis

Materials and Methods

Caenorhabditis elegans strain list

Strain reference	Genotype	Usage
N2	Wild Type	NGS; IP-LFQP and RIP
EG7833	*oxTi559[Peft-3::tdTomato::H2B::unc-54 3'UTR + Cbr-unc-119] I; unc-119(ed3)* III	CRISPR/Cas9
EG7893	*oxTi615[Peft-3::tdTomato::H2B::unc-54 3'UTR + Cbr-unc-119]; unc-119(ed3)* III	CRISPR/Cas9
EG8897	*unc-119(ed3)* III*; oxTi947[Peft-3::GFP::2xNLS::tbb-2 3'UTR + Cbr-unc-119]* V	CRISPR/Cas9
HT1593	*unc-119(ed3)* III	miniMos Transgenes
KK359	*tofu-6(it20); unc-4(e120)/mnC1 dpy-10(e128) unc-52(e444)* II.	
QA137	*tofu-6(yt2)* II*; ytEx100*	
RFK180	*mjls144[Pmex-5::egfp::his-58::21UR-1_as::tbb-2(3'UTR)]; pid-1(xf14)* II	
RFK182	*pid-1(xf35)* II	NGS
RFK183	*pid-1(xf36)* II	
RFK184	*mjSi22[Pmex-5::mCherry::his-58::21UR-1_as::tbb-2(3'UTR)] I; pid-1(xf35)* II	Microscopy
RFK514	*unc-119(ed3)* III*; ife-3(xf101); oxTi947[Peft-3::GFP::2xNLS::tbb-2 3'UTR + Cbr-unc-119]/ oxTi664[Peft-3::TdTomato::H2B::unc-54 3'UTR + Cbr-unc-119]* V	
RFK515	*unc-119(ed3)* III*; ife-3(xf102); oxTi947[Peft-3::GFP::2xNLS::tbb-2 3'UTR + Cbr-unc-119]/ oxTi664[Peft-3::TdTomato::H2B::unc-54 3'UTR + Cbr-unc-119]* V	NGS
RFK523	*pid-3(tm2417)* I*/hT2[bli-4(e937) let-?(q782) qIs48](*I;III*).*	NGS
RFK625	*unc-119(ed3)* III*; xfIs137[Ppid-3::pid-3::mCherry::Myc::pid-3(3'UTR); Cbr-unc-119]* II.	Microscopy
RFK647	*pid-1(xf14); mjls144[Pmex-5::egfp::his-58::21UR-1_as::tbb-2(3'UTR)]* II*; xfIs117[Ppid-1::pid-1::mCherry::V5::pid-1(3'UTR); Cbr-unc-119]* V	RNAi essay
RFK679	*pid-3(tm2417); xfIs136[Ppid-3::pid-3::mCherry::Myc::pid-3 (3'UTR); Cbr-unc-119]* I	IP-LFQP and RIP
RFK684	*xfIs123[Ptofu-6::tofu-6::GFP::HA::tofu-6(3'UTR); Cbr-unc-119]* V	Microscopy
RFK696	*xfIs121[Pife-3::3xFLAG::mCherry::ife-3::ife-3(3'UTR) + Cbr-unc-119]* II*; ife-3(xf101); oxTi947[Peft-3::GFP::2xNLS::tbb-2 3'UTR + Cbr-unc-119]* V	IP-LFQP and RIP

RFK697	xfIs121[Pife-3::3xFLAG::mCherry::ife-3::ife-3(3'UTR) + Cbr-unc-119] II; xfIs123 [Ptofu-6::tofu-6::GFP::HA::tofu-6(3'UTR) + Cbr-unc-119] V	Microscopy
RFK700	xfIs136[Ppid-3::pid-3::mCherry::Myc::pid-3(3'UTR); + Cbr-unc-119] I;xfIs123 [Ptofu-6::tofu-6::GFP::HA::tofu-6(3'UTR) + Cbr-unc-119] V	Microscopy
RFK701	xfIs136[Ppid-3::pid-3::mCherry::Myc::pid-3(3'UTR); + Cbr-unc-119] I; pid-1(xf35) II	IP-LFQP
RFK703	bnIs1[Ppie-1::GFP::pgl-1 + unc-119(+)], xfIs136[Ppid-3::pid-3::mCherry::Myc::pid-3(3'UTR)+ Cbr-unc-119] I	Microscopy
RFK721	tofu-6(it20), unc-4(e120) II; xfIs123[Ptofu-6::tofu-6::GFP::HA::tofu-6(3'UTR)+ Cbr-unc-119] V	IP-LFQP
RFK742	xfIs167[Perh-2::erh-2::EGFP::OLLAS::erh-2 (3'UTR) + Cbr-unc-119] I; erh-2(xf168), oxTi615[eft-3p::tdTomato::H2B::unc-54 3'UTR + Cbr-unc-119]; unc-119(ed3) III	IP-LFQP and Microscopy
RFK810	erh-2(xf168); oxTi615[Peft-3::tdTomato::H2B::unc-54 3'UTR + Cbr-unc-119]/ qC1[dpy-19(e1259) glp-1(q339) qIs26] III	NGS
RFK861	tost-1(xf191); oxTi615[Peft-3::tdTomato::H2B::unc-54 3'UTR + Cbr-unc-119] III	
RFK874	pid-3(xf149), oxTi559[Peft-3::tdTomato::H2B::unc-54 3'UTR + Cbr-unc-119] III/ hT2[bli-4(e937) let-?(q782) qIs48](I;III)	
RFK875	pid-3(xf153), oxTi559[Peft-3::tdTomato::H2B::unc-54 3'UTR + Cbr-unc-119] III/ hT2[bli-4(e937) let-?(q782) qIs48](I;III)	
RFK876	pid-3(xf151), oxTi559[Peft-3::tdTomato::H2B::unc-54 3'UTR + Cbr-unc-119] III/ hT2[bli-4(e937) let-?(q782) qIs48](I;III)	
RFK905	tost-1(xf194), oxTi615[Peft-3::tdTomato::H2B::unc-54 3'UTR + Cbr-unc-119] III/ qC1[dpy-19(e1259) glp-1(q339) qIs26] III	NGS
RFK912	tost-1(xf196), oxTi615[Peft-3::tdTomato::H2B::unc-54 3'UTR + Cbr-unc-119] III	Temperature Shift

Mutant generation with CRISPR/Cas9 system

Mutant alleles were generated as described in (Friedland et al. 2013). gRNAs were selected under the criteria: NGG PAM site, highest GC content and specificity according to CRISPRdirect (Naito et al. 2015) and Zhang Lab's http://crispr.mit.edu. Two to three gRNA, singularly cloned into Addgene plasmid #46169, were injected (35ng/μl) together with Addgene plasmid

#46168 (50ng/μl) and co-injection marker pRR83 (5ng/μl) into adult worms (specific strains below). F1 worms positive for pharynx GFP expression were isolated, allowed reproduction, lysed in single worm lysis buffer (5 mM KCl, 2,5 mM MgCl2, 10 mM Tris pH=8,3, 0,45% IGEPAL, 0,45% Tween-20, 0,01% gelatin) and genotyped for mutations using NEB Taq DNA Polymerase (M0273X) according to manufacturer's instructions. Isolated mutants were outcrossed at least two times before balancing.

**Target**	**CRISPR/Cas9 Guide RNA**
ife-3	GCCTCCGTGCCGGGATTCGA
ife-3	GACACCCCCTCCAGAATCGC
ife-3	GAGCCCAGCGATTCTGGAGG
pid-3	gaaaATGGTTGCCCATCAGA
pid-3	GTGGAAGAATGTGCACGACG
pid-3	GGCGGATTTCAAGTCGAAAT
erh-2	gtgagaattattatgtttaa
erh-2	GAGCAGCTGATTTCTTGGAA
erh-2	GAAGATCATCATAGAAACAT
tost-1	GATAGTTctgaaacataacc
tost-1	GAGCTTCTTCTCATCAGTAG
tost-1	GATGGCAGTAGTCATtctga

miniMos transgene insertion and mapping

Random miniMos insertions were made through injection of unc-119(ed3) carrying worms. C. briggsae unc-119 was used as a selection marker. Injections and mapping were made in accordance to (Frokjaer-Jensen et al. 2014).

Embryonic arrest and transgene complementation tests

Embryonic arrest stage was determined by single picking wildtype and mutant gravid individuals, bleaching and synchronizing them in M9 buffer for 16h. Embryos and larvae were then imaged with wide-field microscopy.

Individuals carrying mutant alleles or carrying both mutant allele and corresponding miniMos transgene were singled at L4 larvae (n>5) stage and allowed to self-fertilize and reproduce for 24h. At this time point progenitors were removed from the plate and embryos/larvae counted. After 48h the number of larvae in each plate was counted and the proportion of arrested embryos for each progenitor was determined.

Immunostaining

Adult worms were dissected in Egg Buffer (25mM HEPES pH 7,4, 118mM NaCl, 48mM KCl, 2mM EDTA, 0,5mM EGTA) with 1%(v/v) Tween20 and fixed 5 minutes by adding 1:1 Egg Buffer+2% formaldehyde followed by a wash step in Egg Buffer. Cuticle was then removed by Freeze cracking (Duerr 2013). An extra fixation step of 1 minute in -20°C Methanol preceded three washes in PBS (137mM NaCl, 2,7mM KCl, 10mM Na_2HPO_4, 2mM KH_2PO_4, pH=7,5) with 0,5%(v/v) Tween20 (PBST). After 1 hour in blocking buffer (PBST+10% Bovine Serum) samples were co-stained overnight at 4°C with 1:200 dilutions of RFP-Booster_Atto647N (Chromotek, rba647n-10) and GFP-Booster_Atto488 (Chromotek, gba488-10). Staining was followed with multiple PBST washes and samples were mounted in ProLong Gold Antifade Mountant (ThermoFisher Scientific, P36930).

RNAi experiments

HT115(DE3) bacteria carrying Timmons and Fire L4440 RNAi feeding vector (Timmons and Fire 1998) were grown over 10 hours and seeded directly onto RNAi plates (standard NGM; 1mM IPTG+and; 50μg/mL ampicillin). HT115(DE3) with empty L4440 or carrying *pid-3* or *tost-1* targeting RNA were taken from the Ahringer RNAi library (Kamath et al. 2003). Remaining

vectors were made by inserting cDNA of its corresponding gene into L4440 and then retransformed into HT115(DE3).

Animals were synchronized at L1 larvae and seeded into RNAi plates containing induced bacteria. For RT-qPCR, worms were imaged in adulthood and harvested for RNA collection. Experiment was repeated three independent times.

RNA isolation

C. elegans were collected off plate and washed with M9 buffer(22mM KH_2PO_4, 42mM Na_2HPO_4, 85mM NaCl, 1mM $MgSO_4$) followed by a wash with ultrapure water and lysis in Worm Lysis Buffer (0,2M NaCl, 0,1M Tris pH=8,5, 50mM EDTA, 0,5% SDS) with 1mg/mL Proteinase K (Sigma-Aldrich, P2308) for 30 minutes at 65°C. After pelleting and removing debris, three volumes of TRIzol LS (ThermoFisher Scientific, 10296-028) were added to sample and RNA precipitation was carried out according to producer's instructions with the aid of Phase lock Gel – Heavy tubes (QuantaBio, 2302830). Eluted RNA samples were depleted of DNA using TURBO DNA-free Kit (Ambion, AM1907).

RNA Immunoprecipitation samples (see below) were obtained by adding TRIzol LS directly to IP beads after washes. The following isolation steps follow the above described process.

RNA RppH and CIP-RppH treatment For RppH treatments a portion of an RNA sample was collected and treated with RNA 5′ Pyrophosphohydrolase (RppH) (NEB, M0356) for the purpose of removing 5′ Cap structures (Almeida et al. 2019). Each was treated in ThermoPol Buffer (20 mM Tris.Cl, 10 mM $(NH_4)_2SO_4$, 10 mM KCl, 2 mM $MgSO_4$, 0,1% Triton X-100, NEB, B9005) for 1 hour at 37°C with 10 units of RppH. Reaction was stopped by adding EDTA to 10 mM and heating to 65°C for 5 minutes. RNA was purified by ethanol precipitation. CIP-RppH samples were treated in CutSmart Buffer (NEB B7204S) with Calf Intestinal Phosphatase (NEB,

M0290L) at 20U/10μg RNA and 0.5U/μl SUPERase.In RNase Inhibitor (Ambion, AM2696) for 1h at 37°C. After TRIzol re-purification samples were treated with RppH as described above.

RT-qPCR

Cultured worms and RNA samples were isolated as described above. Reverse transcription for each sample was performed with 500ng of total RNA using ProtoScript First Strand cDNA Synthesis Kit (NEB, E6300) and Oligo d(T)$_{23}$VN. qPCR 10μl reactions were set up with iTaq Universal SYBR Green Supermix (Bio-Rad, 1725121), 500mM primer concentration and a volume ratio of 1/5 cDNA. PCR cycles and measurements were made in an Applied Biosystems ViiA7 Real Time PCR System (ThermoFisher Scientific). Cycling conditions were made according to iTaq manufacture's recommendations: Standard run, temperature increments of 1,6°C/s; 95°C for 30 seconds, 40 cycles of 95°C for 15 seconds and 60°C for 1 minute; melt curve calculation: 15 seconds at 95°C, 1 minute at 60°C, temperature increments of 0,05°C/s to 95°C and hold for 15 seconds. Technical duplicates and biological triplicates were used. ΔΔCT method was used as an analysis method (Schmittgen and Livak 2008). *pmp-3* was used as a normalization factor (Hoogewijs et al. 2008). Error bars represent the standard deviation of three biological replicates. Used primers are listed below.

**Target**	**Sequence**
*pmp-3*_Fw	GTTCCCGTGTTCATCACTCAT
*pmp-3*_Rev	ACACCGTCGAGAAGCTGTAGA
*GFP*_Fw	ATGGTGTTCAATGCTTCTCG
*GFP*_Rev	TGACTTCAGcacgtgTCTTGT

tost-1(xf196) **temperature shift assays**

Viable progeny quantification RFK912 worms were cultured at 15°C on standard plates. At the start of experiment they were selected and singled into standard plates at L4

larvae stage. After overnight (O/N) culture at 15°C or 25°C individual worms were transferred into a new plate and shifted to corresponding temperature together with the plate of O/N egg lay. Every 2 hours individuals were transferred into a new plate. Eggs were counted in each of these plates on the day of egg lay and two days after larvae were counted for survival assay. As control we include RFK912 worms which underwent the same treatment except with no temperature shift and transferred into new plates every 4 hours.

Small RNA sequencing Synchronized RFK912 and N2 worms were cultured at 15°C on standard plates. At gravid adult stage plates were shifted to 25°C and collected after 4h and 12h and RNA was isolated as indicated above. 0h indicates non-temperature shifted plates.

Small RNA Library preparation and sequencing

NGS library prep was performed with NEXTflex Small RNA-Seq Kit V3 following Step A to Step G of Bioo Scientific`s standard protocol (V16.06). Libraries starting amount and PCR cycles can be consulted in the table below. Amplified libraries were purified by running an 8% TBE gel and size-selected for 18 – 40nt. Libraries were profiled in a High Sensitivity DNA on a 2100 Bioanalyzer (Agilent technologies) and quantified using the Qubit dsDNA HS Assay Kit, in a Qubit 2.0 Fluorometer (Life technologies). Samples of each individual experiment were pooled in equimolar ratio. Sequences were deposited at SRA, submission number PRJNA503945.

Experiment	Sample (each 3x)	Starting Material	PCR cycles	Equipment	Run type
pid-3	*pid-3(tm2417)*	1000ng	15	NextSeq 500 Flowcell	Highoutput 75-cycle-kit, SR for 1x 83 cycles plus 7 cycles for the index read
	pid-3(tm2417)/+	1000ng	15	NextSeq 500 Flowcell	Highoutput 75-cycle-kit, SR for 1x 83 cycles plus 7 cycles for the index read
ife-3	*ife-3(xf102)*	500ng	15	NextSeq 500/550 Flowcell	SR for 1x 75 cycles plus 7 cycles for the index read

	N2	500ng	15	NextSeq 500/550 Flowcell	SR for 1x 75 cycles plus 7 cycles for the index read
erh-2	*erh-2(xf168)*	500ng	15	NextSeq 500/550 Flowcell	SR for 1x 75 cycles plus 7 cycles for the index read
	erh-2(xf168)/+	500ng	15	NextSeq 500/550 Flowcell	SR for 1x 75 cycles plus 7 cycles for the index read
tost-1	*tost-1(xf194)*	2000ng	12	NextSeq 500 Flowcell	Midoutput 150-cycle-kit, PE for 2x 75 cycles plus 16 cycles for the index read
	pid-1(xf35)	2000ng	12	NextSeq 500 Flowcell	Midoutput 150-cycle-kit, PE for 2x 75 cycles plus 16 cycles for the index read
	N2	2000ng	12	NextSeq 500 Flowcell	Midoutput 150-cycle-kit, PE for 2x 75 cycles plus 16 cycles for the index read

Biochemistry

Worm preparation Synchronized non-gravid adult worms were collected off plate and washed with M9 buffer followed by a wash with ultrapure water. Pellets were frozen with liquid nitrogen and kept at -80°C until usage.

Lysate preparation Worm Pellets were thawed on ice and mixed 1:1 with 2x Lysis Buffer (20 mM Tris.Cl, 300 mM NaCl, 1 mM EDTA, 1%(v/v) IGEPAL CO-630, pH 7,5) with 2x protease inhibitors (cOmplete Mini, EDTA-free, Roche, 11836170001). Bioruptor Plus (Diagenode) sonicator was used to lyse worms (10 cycles 30/30 seconds, 4°C, high energy) and debris removed by spinning. Lysate protein concentration was determined with Pierce BCA Protein Assay Kit (ThermoFisher Scientific, 23225).

Immunoprecipitations Lysates were diluted in 1x Lysis Buffer+ 1x Protease inhibitors to a final concentration of 1,5 mg of protein/mL and a total of 0,75 mg of protein was used per IP. At this step input samples were collected into 2x NuPAGE LDS Sample buffer (Life Technologies, NP0007)+ 200 mM DTT and boiled for 10 minutes. Anti-mCherry IPs were

performed with RFP-Trap_MA beads (Chromotek, rtma-20) and anti-GFP IPs with GFP-Trap_MA beads (Chromotek, gtma-20), in both cases 25 μl of bead slurry was used and samples were rotated at $4^{o}C$ for 2 hours. Subsequent washes were made with Wash Buffer (10 mM Tris.Cl, 150 mM NaCl, 0,5 mM EDTA, pH 7,5)+ Protease inhibitors in accordance with Chromotek protocols. Washed beads were resuspended in 2x NuPAGE LDS Sample Buffer + 200 mM DTT and boiled for 10 minutes, making the samples ready for loading.

RNAse treated immunoprecipitations followed the above described protocol with an additional RNAse A/T1 Mix (ThermoFisher Scientific, EN0551) treatment step. After lysate dilution samples were divided in two (Control and +RNAse) and 20 μL of RNAse A/T1 mix was added per 1 mL of +RNase sample. Control and +RNase samples were rotated for 20 minutes at $4^{o}C$ and followed by the described IP protocol.

Western blot Inputs an IP samples were loaded into 4-12% gradient gels (ThermoFisher, NP0321BOX) and run with 1x NuPAGE MES SDS Running Buffer (ThermoFisher, NP0002). Transfer to an Immobilon PVDF, 0,45 μm membrane (Merck Millipore, IPVH00010) was executed with 1x NuPAGE Transfer Buffer (ThermoFisher Scientific, NP0006) 20%(v/v) Methanol. Membrane was probed with rabbit anti-PID-1 Q5941 (de Albuquerque et al. 2014) and detected with Amersham ECL Select Western Blotting Detection Reagent (GE Healthcare, RPN2235). Background recognition by anti-PID-1 ab is used as loading control.

Endogenous PID-1 Immunoprecipitations 200 μL of synchronized adult worms were resuspended in 500 μL of IP lysis buffer (25 mM Tris pH 7,5, 150 mM NaCl, 1,5 mM $MgCl_2$, 1 mM DTT, 0,1% Triton X-100, complemented with 2x protease inhibitor) and sonicated at $4^{o}C$ for 10 cycles of 30/30 seconds, high intensity using a Bioruptor Plus (Diagenode). Cell debris was removed via spinning and 30 μL of washed Dynabeads Protein G (Life Technologies, 1004D) and 10 μL of anti-PID-1 antibody (Q5941) was added to the lysates and incubated

under rotation for 3 hours at 4°C. The beads were then washed 3x 5 minutes in wash buffer (25 mM Tris pH 7,5, 150 mM NaCl, 1,5 mM $MgCl_2$, 1 mM DTT, complemented with 2x protease inhibitor) and resuspended in 30 µL of NuPAGE LDS buffer.

Mass Spectrometry Samples were separated on a 4–12% gradient Bis-Tris gel (ThermoFisher, NP0321) in MOPS SDS Running Buffer (ThermoFisher, NP0001) at 180 V for 10 minutes, afterward separately processed by in-gel digest (Shevchenko et al. 2006) (Kappei et al. 2013) and desalted using a C18 StageTip (Rappsilber et al. 2007). The digested peptides were separated on a 25cm reverse-phase capillary (75µm inner diameter) packed with Reprosil C18 material (Dr. Maisch). Separation of the peptides was done with the EASYnLC 1000 system (Thermo) along a 2 hour gradient increasing from 2 to 40% Buffer B. For PID-1 IPs the gradient was shortened to 90 minutes. Measurement was done on a Q Exactive Plus mass spectrometer (Thermo) operated with a Top10 data-dependent MS/MS acquisition method per full scan (Bluhm et al. 2016). Measurements were processed with MaxQuant version 1.5.2.8 (Cox and Mann 2008) using the wormbase protein fasta database (version WS265) and standard settings except LFQ quantitation and match between runs were activated. The mass spectrometry proteomics data have been deposited to the ProteomeXchange Consortium via the PRIDE partner repository with the dataset identifier PXD011500.

Size exclusion chromatography-Western blot

PID-1 was detected in 3xFLAG::RRF-3 adult extract fractions were refurbished from (Almeida et al. 2018). Lysates for TOFU-6::GFP::HA and ERH-2::GFP::OLLAS were prepared as described above. 4,5mg of total protein were separated on a Superose 6, 10/300 GL size exclusion column (GE Healthcare, 17517201), using a NGC Quest system (BioRad) and samples

were collected as described in (Almeida et al. 2018). Amicon Ultra 3kDa cutoff filter units (Merck-Millipore, UFC500324) were used to 13x concentrate each fraction and 25% of sample was used for SDS-PAGE/Western blot. TOFU-6::GFP::HA, ERH-2::GFP::OLLAS and PID-1 proteins were detected in independent extracts with antibodies anti-HA (Sigma-Aldrich, SAB4300603-100UG), anti-GFP (Santa Cruz Biotechnology, sc-9996) and anti-PID-1 (Q5941), respectively.

RIPseq

Lysate preparation Worm Pellets were thawed on ice and mixed 1:1 with 2x Lysis Buffer (20 mM Tris.Cl, 300 mM NaCl, 1 mM EDTA, 1%(v/v) IGEPAL CO-630, pH 7,5) with 2x protease inhibitors (cOmplete Mini, EDTA-free, Roche, 11836170001) and 2x SUPERase.In RNase Inhibitor (Ambion, AM2696). Bioruptor Plus (Diagenode) sonicator was used to lyse worms (10 cycles 30/30 seconds, high energy). Lysate protein concentration was determined with Pierce BCA Protein Assay Kit (ThermoFisher Scientific, 23225). Lysates were diluted in 1x Lysis Buffer+ 1x Protease inhibitors+ 1x SUPERase.In RNase Inhibitor to a final concentration of 1,5 mg of protein/mL and a total of 2,1 mg of protein was used per IP. Each lysate was cleared with 225 μL of Binding Control magnetic agarose beads (Chromotek, bmab-20) for 1 hour at 4°C.

Immunoprecipitation Quadruplicate anti-mCherry RIPs were performed with RFP-Trap_MA beads (Chromotek, rtma-20). 75 μl of bead slurry per sample blocked for 1 hour with Blocking Buffer [2% (w/v) BSA, 2,5 mg/mL tRNA from *E.coli* MRE 600 (SigmaAldrich, 10109541001), 10 mM Tris.Cl, 150 mM NaCl, 0,5 mM EDTA, pH 7,5] and washed with Wash Buffer (10 mM Tris.Cl, 150 mM NaCl, 0,5 mM EDTA, pH 7,5). Inputs from cleared lysates were

taken and mixed 3:1 with TRIzol. 75 μl of blocked bead slurry was added to the remaining cleared lysate and samples were rotated at 4°C for 2 hours. Subsequent washes were made with Wash Buffer+ Protease inhibitors. Washed beads were resuspended in 100 μL of Nuclease free water and immediately mixed with 400 μL of TRIzol.

Library preparation and Sequencing NGS library prep was performed with NEXTflex Small RNA-Seq Kit V3 following Step A to Step G of Bioo Scientific`s standard protocol (V16.06). Libraries were prepared with a starting amount of 100 ng and amplified in 18 PCR cycles. Amplified libraries were purified by running an 8% TBE gel and size-selected for 18 – 40nt. Libraries were profiled in a High Sensitivity DNA on a 2100 Bioanalyzer (Agilent technologies) and quantified using the Qubit dsDNA HS Assay Kit, in a Qubit 2.0 Fluorometer (Life technologies). Total amount of samples were divided in two pools. Each pool was mixed in equimolar ratio and sequenced on a NextSeq 500/550 Flowcell, SR for 1x 75 cycles plus 7 cycles for the index read.

Bioinformatic analysis

sRNA sequencing analysis Raw reads were first processed to remove adapters with v1.9 cutadapt (https://cutadapt.readthedocs.io/en/stable/) (seqtk trimfq -L 50 | cutadapt -a TGGAATTCTCGGGTGCCAAGG -O 5 -m 26 -M 48), followed by removal of reads containing low quality calls with the FASTX-Toolkit v0.0.14 (fastq_quality_filter -q 20 -p 100 -Q 33). The information of the read sequence and the 5' and 3' random UMIs (NNNN-RNA sequence-NNNN) was then used to collapse reads with identical sequences, including that of the UMIs, using a command-line script. UMIs were then removed (seqtk trimfq -b 4 -e 4), and reads shorter than 15 nucleotides were filtered out (seqtk seq -L 15) before mapping against the *C. elegans* genome (WBcel235, ensembl) with bowtie v0.12.8 (Langmead et al. 2009) (-q –sam –phred33-quals –tryhard –best –strata -v 0 -M 1). Coverage tracks were generated with Bedtools 2.25.0 (Quinlan and Hall 2010) (genomeCoverageBed -bg -split -scale) to summarize genomic read coverage, and the bigwigs created with bedGraphToBigWig. Normalization was done to total mapped reads. For visualization, the alignments of different replicated for the same

sample were merged with bamtools-2.3.0 merge (Barnett et al. 2011). For the RIP-seq experiments, merged alignments were further processed to create $\log_2$(IP/input) normalized tracks using DeepTools (Ramirez et al. 2016) (bigwigCompare –binSize 1 –ratio log2).

Reads mapping to annotated features in the custom GTF were counted with htseq-count v0.9.0 (Anders et al. 2015) (htseq-count -f bam -m intersection-nonempty ~~s reverse)~~. To identify RNA-bound to the complex in RIPseq, we used DESeq2 (Love et al. 2014) with the formula '~replicate+condition' in which each IP is being compared to the corresponding input control. Replicates are paired as they are generated from the same biological sample. All the samples in the dataset were included in construction of the DESeq2 object, in order to estimate the dispersion more robustly.

For the differential expression of 21U and miRNAs we performed pairwise differential gene expression estimation with DESeq2 (Love et al. 2014), using the counts for all conditions in the construction of the DESeq2 object for a more robust dispersion estimate. For visualization, genes belonging to a particular biotype (piRNA or miRNA) were extracted from the final results table and their mean expression versus log2 Fold-Change shown in an MA-plot.

The number of reads mapping to different RNA classes was estimated with a combination of a custom Python script to select reads by size and nucleotide bias, available at https://github.com/adomingues/filterReads/blob/master/filterReads/filterSmallRNAclasses.py, and bedtools intersect to match reads with annotated features. 21U RNAs were defined as reads with 18-40 bases mapping sense (intersectBed -s -f 0.85) to an annotated 21U RNA *locus* for type I, or the type II 21U-RNA identified in Gu et al. (2012), supplementary table 3B, following conversion of the coordinates from WS215 to WBcel235 with crossMap (Zhao et al. 2014). 21U-RNA precursors are reads that map to the -2 position with respect to the mature 5' end of annotated 21U-RNAs and are at least 23 nucleotides in length. 26G RNAs are 26 nucleotide long reads mapping antisense to protein coding genes, pseudogenes and lincRNA. For 26G RNAs a minimum overlap of 1 base was required (intersectBed default). miRNAs were defined as reads mapping sense to annotated miRNAs

(intersectBed -s -f 1.0). Genomic locations were extracted from a custom gtf (genes + transposons) using the biotype information.

Metagene profiles were created with DeepTools. Read coverage was summarized with computeMatrix scale-regions –metagene –missingDataAsZero -b 50 -a 50 –regionBodyLength 98 –binSize 1 –averageTypeBins mean. As SL genes are multicopy genes, the setting –averageTypeBins was set to "sum". Using the "mean" of SL sequences did not alter the profile obtained (data no shown). The final metagene figure was created with plotProfile –plotType lines –perGroup.

Evolutionary analysis of PETISCO Predicted proteomes of *Caenorhabditis* species were downloaded from Caenorhabditis.org. Selected other species were downloaded from Wormbase parasite (parasite.wormbase.org - WBPS5). The proteome of *C. elegans* WS235 was used as the test file for reciprocal blastp searches against all other species, recording only the best hit. The bit score of the best blast hit was extracted for the PETISCO complex, with ERH-1, PRDE-1 and PRG-1 included for comparison. In the case that no best reciprocal blast hit was found, the score was given as 0. Scores were then normalized by dividing by the score from blasting *C. elegans* against itself, to control for the different lengths of the proteins. In the case that no hit was found, genomic nucleotide sequence was downloaded and Exonerate was used to search for potential unannotated orthologues; identified hits were then reconstructed from the predicted protein sequence output from Exonerate and then tested using best reciprocal blastp as above. The heatmap.2 function in gplots within R was used to generate the heatmap. The order of the species in the heatmap was estimated by calculating the mean blastp score for the entire *C. elegans* proteome.

References

Almeida MV, Dietz S, Redl S, Karaulanov E, Hilderbrabdt A, Renz C, Ulrich HD, König J, Butter F, Ketting RF. 2018. GTSF-1 is required for formation of a functional RNA-dependent RNA Polymerase complex in *Caenorhabditis elegans*. *EMBO J* **37**: pii: e99325.

Almeida MV, Domingues AM, Lukas H, Mendez-Lago M, Ketting RF. 2019. RppH can faithfully replace TAP to allow cloning of 5'-triphosphate carrying small RNAs. *MethodsX* **6**: 265-272.

Anders S, Pyl PT, Huber W. 2015. HTSeq—a Python framework to work with high-throughput sequencing data. *Bioinformatics* **31**: 166-169.

Bluhm A, Casas-Vila N, Scheibe M, Butter F. 2016. Reader interactome of epigenetic histone marks in birds. *Proteomics* **16**: 427-436.

Cox J, Mann M. 2008. MaxQuant enables high peptide identification rates, individualized p.p.b.-range mass accuracies and proteome-wide protein quantification. *Nat Biotechnol* **26**: 1367-1372.

de Albuquerque BF, Luteijn MJ, Cordeiro Rodrigues RJ, van Bergeijk P, Waaijers S, Kaaij LJ, Klein H, Boxem M, Ketting RF. 2014. PID-1 is a novel factor that operates during 21U-RNA biogenesis in Caenorhabditis elegans. *Genes Dev* **28**: 683-688.

Duerr JS. 2013. Antibody staining in C. elegans using "freeze-cracking". *J Vis Exp*.

Friedland AE, Tzur YB, Esvelt KM, Colaiacovo MP, Church GM, Calarco JA. 2013. Heritable genome editing in C. elegans via a CRISPR-Cas9 system. *Nat Methods* **10**: 741-743.

Frokjaer-Jensen C, Davis MW, Sarov M, Taylor J, Flibotte S, LaBella M, Pozniakovsky A, Moerman DG, Jorgensen EM. 2014. Random and targeted transgene insertion in Caenorhabditis elegans using a modified Mos1 transposon. *Nat Methods* **11**: 529-534.

Goh WS, Seah JW, Harrison EJ, Chen C, Hammell CM, Hannon GJ. 2014. A genome-wide RNAi screen identifies factors required for distinct stages of C. elegans piRNA biogenesis. *Genes Dev* **28**: 797-807.

Hoogewijs D, Houthoofd K, Matthijssens F, Vandesompele J, Vanfleteren JR. 2008. Selection and validation of a set of reliable reference genes for quantitative sod gene expression analysis in C. elegans. *BMC Mol Biol* **9**: 9.

Kamath RS, Fraser AG, Dong Y, Poulin G, Durbin R, Gotta M, Kanapin A, Le Bot N, Moreno S, Sohrmann M et al. 2003. Systematic functional analysis of the Caenorhabditis elegans genome using RNAi. *Nature* **421**: 231-237.

Kappei D, Butter F, Benda C, Scheibe M, Draskovic I, Stevense M, Novo CL, Basquin C, Araki M, Araki K et al. 2013. HOT1 is a mammalian direct telomere repeat-binding protein contributing to telomerase recruitment. *EMBO J* **32**: 1681-1701.

Naito Y, Hino K, Bono H, Ui-Tei K. 2015. CRISPRdirect: software for designing CRISPR/Cas guide RNA with reduced off-target sites. *Bioinformatics* **31**: 1120-1123.

Rappsilber J, Mann M, Ishihama Y. 2007. Protocol for micro-purification, enrichment, pre-fractionation and storage of peptides for proteomics using StageTips. *Nat Protoc* **2**: 1896-1906.

Schmittgen TD, Livak KJ. 2008. Analyzing real-time PCR data by the comparative C(T) method. *Nat Protoc* **3**: 1101-1108.

Shevchenko A, Tomas H, Havlis J, Olsen JV, Mann M. 2006. In-gel digestion for mass spectrometric characterization of proteins and proteomes. *Nat Protoc* **1**: 2856-2860.

Timmons L, Fire A. 1998. Specific interference by ingested dsRNA. *Nature* **395**: 854.

Legends to supplemental Figures

Figure S1. PID-1 interacts with a restricted set of proteins

(A) Biological replicate of interaction data as described in Fig. 1A.

(B) mCherry pull-down of wild type (WT), *pid-1(xf14)* mutant and PID-3::mCherry::Myc;*pid-3(tm2417)* (PID-3::mCh) carrying worms. IPs were performed in non-gravid adult extracts. Membrane was probed for endogenous PID-1. Background recognition by the anti-PID-1 antibody is used as loading control.

(C) Schematic representation of domain composition of PETISCO components used in the Y2H grid. In all cases proteins were fused to the C-terminal part of budding yeasts' GAL4 activation or binding domains.

Figure S2. PETISCO subunit transgenes complement mutant phenotypes

- **(A)** Percentage of arrested embryos in strains carrying PETISCO mutations and corresponding transgenes. In all cases the progeny from homozygous second generation was counted from at least five individuals.
- **(B)** Complementation test of *ife-3(xf101)* vs *ife-3(xf101);xfIs123* carrying individuals. n>10 individuals.
- **(C)** Embryos from wild-type (N2) and PETISCO homozygous mutant mothers, one day after laying. The mutant embryos arrest between 64-128 cell stage, while the wild-type embryos have mostly hatched (left N2 image). Occasional non-hatching wild-type embryos (right N2 image) do not show the characteristic stage of the PETISCO mutants. Scale bar represents 10μm.

Figure S3. PID-1 interactor miniMos transgenes

(A) Schematic representation of miniMos transgene insertions for each of the PID-1 interactors. *C. briggsae unc-119* is used as selection marker.

(B) Expression pattern of 3xFLAG::mCherry::IFE-3 under endogenous promotor and 3'UTR in germline and embryos. PGL-1::GFP is used as a P-granule marker. Images depict live worms under the wide field fluorescent microscope. Circle and arrow highlight PGL-1:IFE-3 co-localization. Scale Bar represents 10 μm. Contrast of images has been enhanced.

(C and D) Expression pattern of the indicated transgenes of PID-1 and its interactors under respective endogenous promotors and 3'UTR. Images depict live **(C)** embryos or **(D)** adult worms under the wide field fluorescent microscope. Scale Bar represents **(C)** 10 μm and **(D)** 20 μm. Contrast of images has been enhanced.

Figure S4. PETISCO is a stably interacting complex

(A-C) Volcano plots representing label-free proteomic quantification of quadruplicate anti-mCherry IPs from non-gravid adult extracts. In **(A)** and **(B),** respectively, PID-3::mCherry::Myc;*pid-3(tm2417)*; or 3xFLAG::mCherry::IFE-3;*ife-3(xf101)*; were used for immunoprecipitation with or without RNAse A/T1 treatment.. Worms were grown in high density plates for these particular experiments. In **(C)** standard IP-MS protocol was used with PID-3::mCherry::Myc;*pid-1(xf35)* worms. In all cases the x-axis represents the median fold enrichment of individual proteins in control (WT) versus transgenic strain. y-axis indicates -Log_{10}(p-value) of observed enrichments. Dashed lines represent thresholds at p=0.05 and 2-fold enrichment. Blue data points represent values out of scale. Red and Green data points represent above and below threshold respectively.

Figure S5. Yeast Two Hybrid interaction assays of PETISCO

(A-D) Yeast two-hybrid interaction assays of PETISCO subunits in low stringency (TRP^{-}LEU^{-}HIS^{-}), high stringency (TRP^{-}LEU^{-}HIS^{-}ADE^{-}) or control (TRP^{-}LEU^{-}) plates as indicated. **(A)** Full length proteins **(B)** TOFU-6 and individual domains tested for interaction with full length IFE-3 **(C)** Interactions between PID-3 and TOFU-6 **(D)** Interactions with ERH-1.

Figure S6. PETISCO mutant small RNA sequencing and gene structure.

A) Schematic representation of individual gene structures of PETISCO subunits. Alleles are indicated in figure.

(B ,C and E) Global levels of **(B)** miRNAs **(C)** type I 21U RNAs and **(E)** type II 21U RNAs and 26G RNAs, in wild type (N2), *ife-3(xf102)*, *pid-3(tm2417)* and *erh-2(xf168)* worms. For the latter two heterozygous populations were used as a control. Values are in reads per million mapped reads (RPM). Individual data points of three independent replicates are shown and horizontal bar represents the total mean. Significance was tested with Student's t-test and p-values are indicated in the graph.

(D) Length distribution and nucleotide bias of mature 21U RNAs type I that remain in PETISCO mutants. Graphs represent the average of the three replicates of **(C)**. Nucleotide bias is represented as color code.

Figure S7. Masculinization of the germline in *pid-1(xf35)* mutant animals

Fluorescent microscopy of the germline of 21U sensor GFP of *pid-1(xf35)*; 21U sensor worms. *pid-1(xf35)* hermaphrodites display a low frequency (<1%) Mog phenotype (second row). In the bottom row a male germline from the same strain is presented and in the top row a hermaphrodite germline of a *pid-1* knock-down. Scale Bars represent 20 μm. Contrast of images has been enhanced.

Figure S8. TOST-1:PID-1 alignment and effects of TOST-1 on small RNA populations

(A) Protein alignment of nematode PID-1 and TOST-1 orthologues. *C. elegans* orthologues are underlined in blue. Conserved motif is shaded in pink.

(B) Global levels of 21U RNAs type I, type II, miRNAs and 26G RNAs in wild type (N2), *pid-1(xf35)* and *tost-1(xf194)* gravid adult worms. Values are in reads per million mapped reads (RPM). Each dot represents a replicate of three and horizontal bar represents the total mean. Significance tested Dunnett's post hoc test and p-values are indicated in the graph.

(C) Temperature shift assays of the temperature sensitive allele *tost-1(xf196)*. L4 larvae were grown overnight (O/N) at 15°C or 25°C and shifted to 25°C or 15°C the next morning already as adults. O/N egg lay was shifted to corresponding temperature in parallel. Each individual was changed into a new plate every 2h and progeny counted at egg stage and L2 larvae. Each point represents the progeny of an individual worm.

(D and E) Pairwise differential gene expression of type I 21U RNAs and miRNAs in *tost-1(xf196)* vs. wildtype (N2) control. In **(D)** samples consist of triplicate synchronized gravid adult populations, exposed to stringent temperatures (25°C) for 0h, 4h or 12h. In **(E)** samples were cultured at stringent temperatures from synchronized L1 larvae and collected as gravid adults. Red and blue dots indicate upregulated and downregulated transcripts respectively.

Figure S9. snRNAs in PETISCO mutants

(A) Read coverage of SL1 RNA and 5S rRNA genes in wild type (N2), *pid-1(xf35)* and *tost-1(xf194)* gravid adult worms and *pid-3(tm2417), erh-2(xf168)* and *ife-3(xf102)* non-gravid adults. Line represents the average of three replicates. Colors under the x-axis correspond to colors represented in Fig. 6B.

(B) Global levels of SL1, SL2, 5S rRNA and U snRNAs in wild type (N2), *pid-1(xf35)* and *tost-1(xf194)* gravid adult worms and *pid-3(tm2417), erh-2(xf168)* and *ife-3(xf102)* non-gravid adults. Values are in reads per million mapped reads (RPM). Individual data points of three independent replicates are shown and horizontal bar represents the total mean. Significance was tested with either Student's t-test *(pid-3, ife-3, erh-2)* or Dunnett's post hoc test (*tost-1, pid*-1)and p-values are indicated in the graph.

Figure S10. snRNAs in PETISCO RIPs

(A) Fold enrichment of SL2 RNAs and U snRNAs in Mock (N2), 3xFLAG::mCherry::IFE-3;*ife-3(xf101)*; and PID-3::mCherry::Myc;*pid-3(tm2417)* RIPs over paired input in non-gravid adult worms. Left panel displays RppH treated and right panel non-treated samples. Individual data points of three or more independent replicates are shown and horizontal bar represents the total mean. Significance was tested with Dunnett's post hoc test and p-values are indicated in the graph.

(B) Coverage profile, normalized to paired input, of SL2 in the indicated strains.

Table S1. Transgene insertion details

Can be downloaded as excel file.

Table S2. Allele information

Can be downloaded as excel file.

Table S3. Sample correlations after normalization

Pairwise correlation values between biological replicates of different conditions. Reads mapping to each annotated genomic feature was counted and normalized to the number of mapped reads as described in methods. These normalized counts were used to estimate the person correlation value using the R function cor.

Table S4. PETISCO subunit mutants display maternal effect lethality

Summary of phenotypes observed in the various mutants described and used in this study. "n.a." not applicable; "n.d." not determined; "-" mild 21U RNA defect; "--" severe 21U RNA defect; "+" no 21U RNA defect; "(TS)" temperature sensitive; "#" counts are for gonadal arms (n=38) due to mixed phenotypes in individuals; "*" according to Goh et al. (2014).

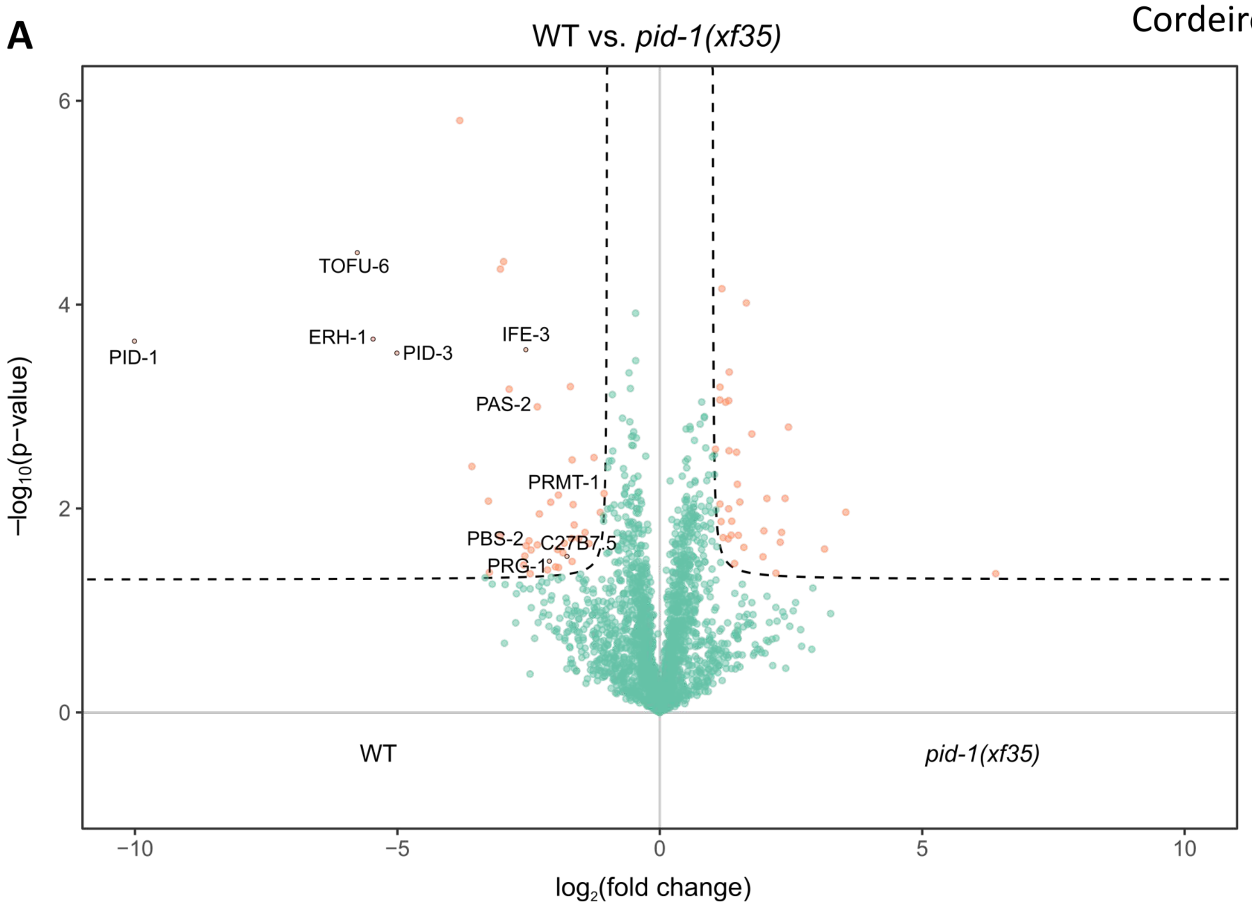
A
WT vs. pid-1(xf35)
−log10(p−value)
log2(fold change)
PID-1
TOFU-6
ERH-1
PID-3
IFE-3
PAS-2
PRMT-1
PBS-2
C27B7.5
PRG-1
WT
pid-1(xf35)

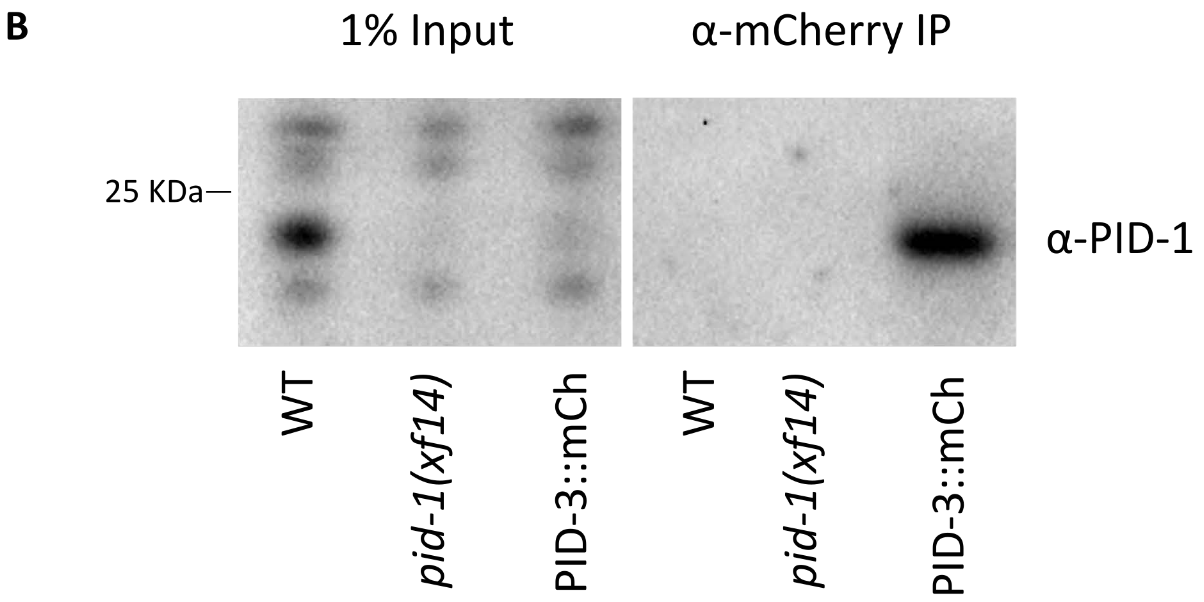
B
1% Input
α-mCherry IP
25 KDa—
α-PID-1
WT
pid-1(xf14)
PID-3::mCh
WT
pid-1(xf14)
PID-3::mCh

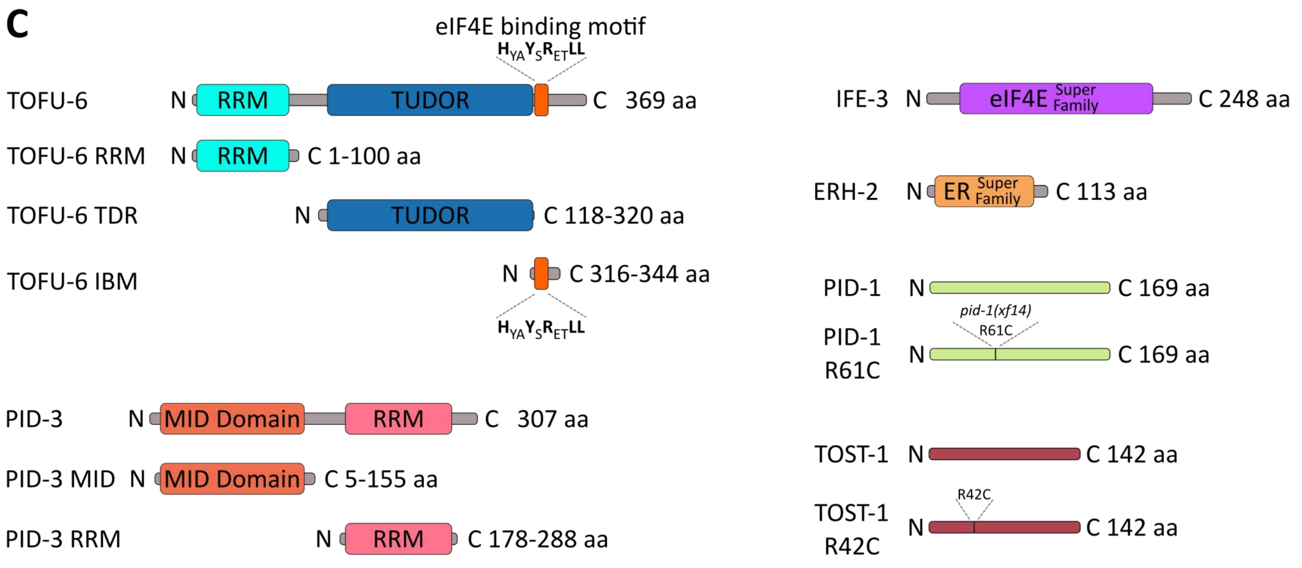
C
eIF4E binding motif
TOFU-6 N RRM TUDOR C 369 aa
TOFU-6 RRM N RRM C 1-100 aa
TOFU-6 TDR N TUDOR C 118-320 aa
TOFU-6 IBM N C 316-344 aa
PID-3 N MID Domain RRM C 307 aa
PID-3 MID N MID Domain C 5-155 aa
PID-3 RRM N RRM C 178-288 aa
IFE-3 N eIF4E Super Family C 248 aa
ERH-2 N ER Super Family C 113 aa
PID-1 N C 169 aa
PID-1 R61C N C 169 aa
pid-1(xf14)
R61C
TOST-1 N C 142 aa
TOST-1 R42C N C 142 aa
R42C

Cordeiro_Rodrigues_FigS2

A

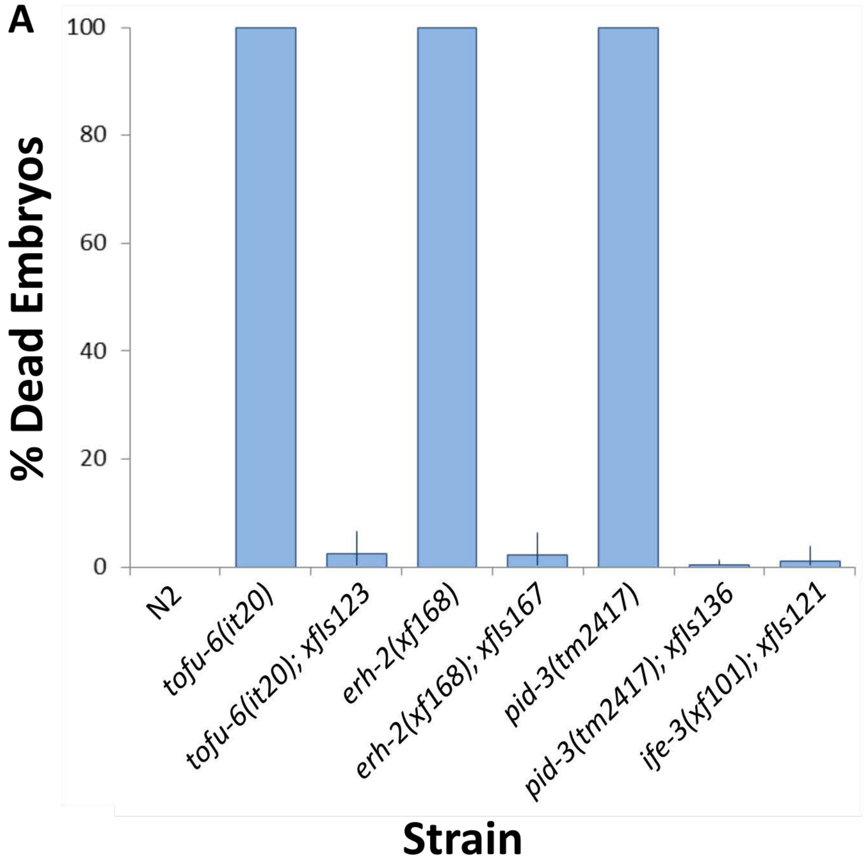

B

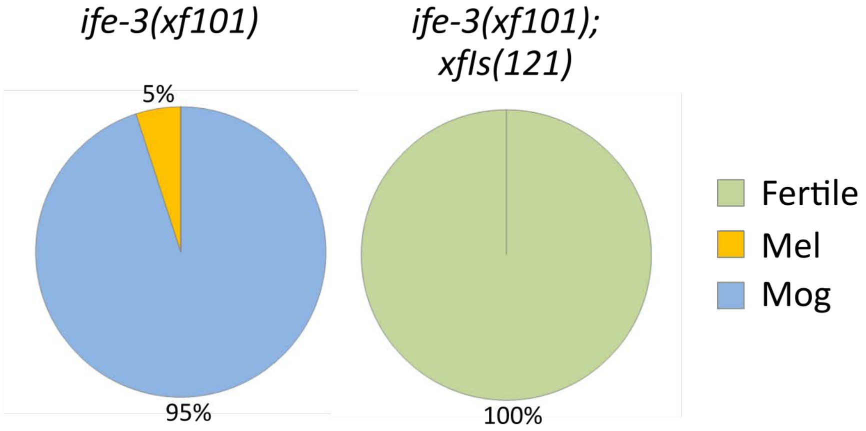

C

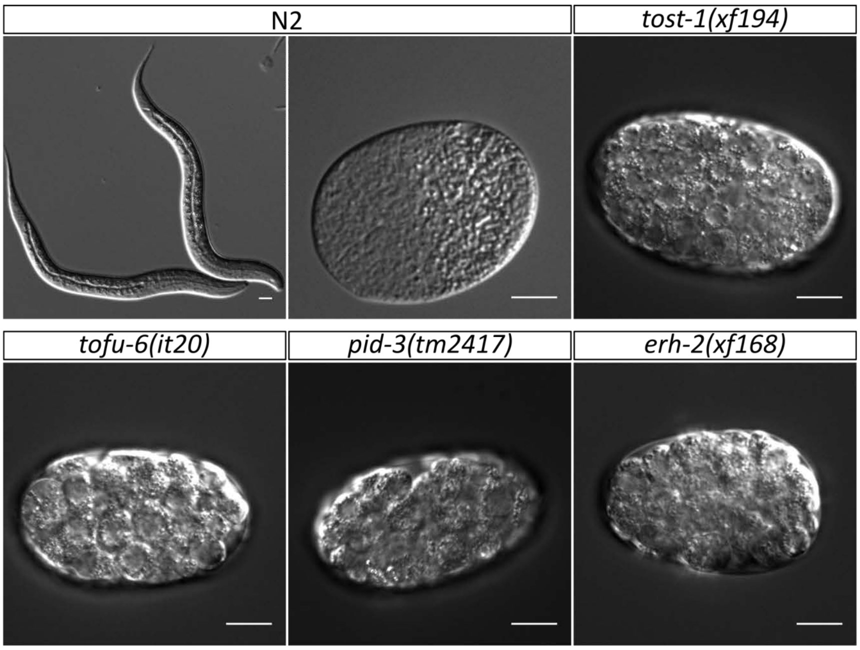

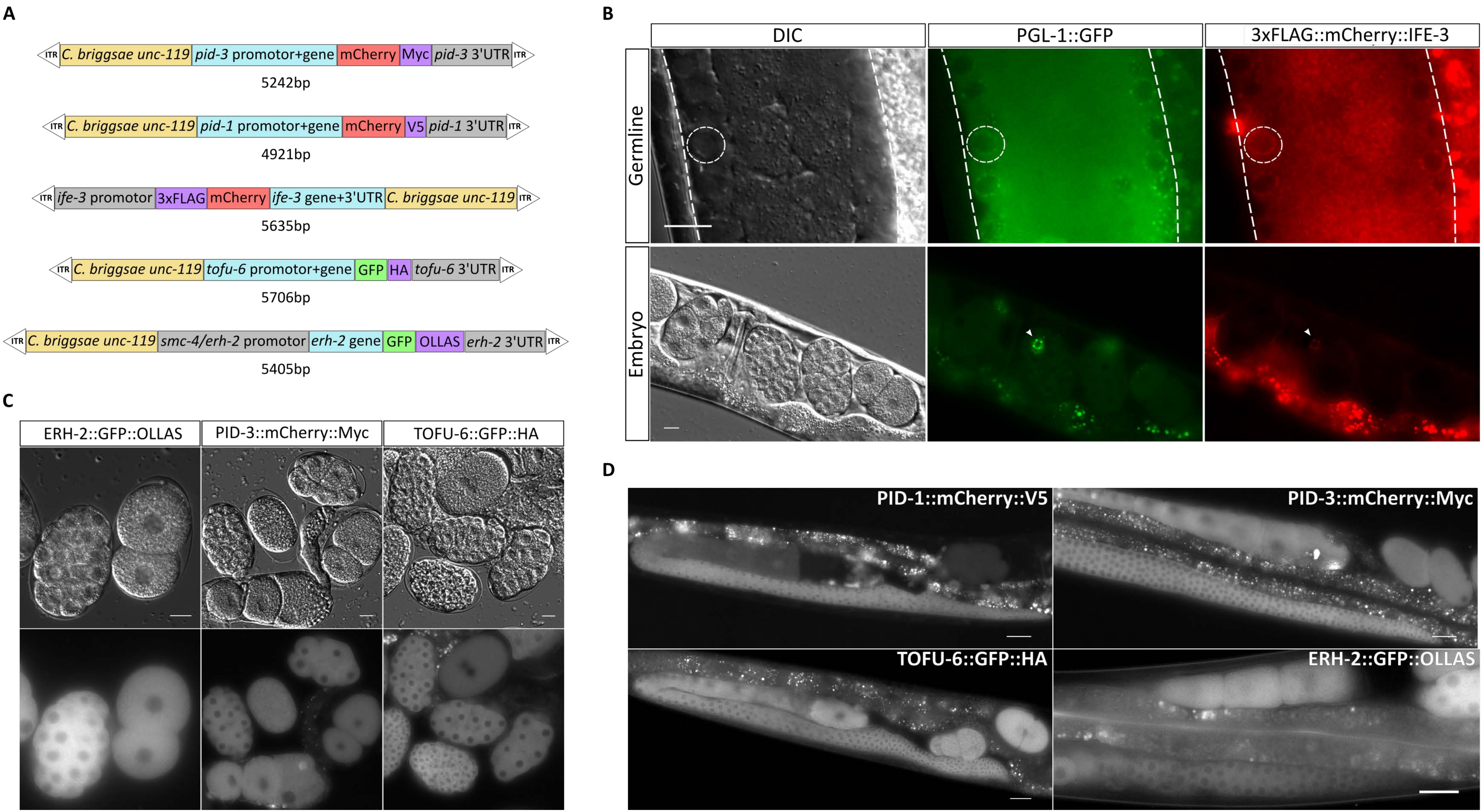
A
ITR C. briggsae unc-119 pid-3 promotor+gene mCherry Myc pid-3 3'UTR ITR
5242bp
ITR C. briggsae unc-119 pid-1 promotor+gene mCherry V5 pid-1 3'UTR ITR
4921bp
ITR ife-3 promotor 3xFLAG mCherry ife-3 gene+3'UTR C. briggsae unc-119 ITR
5635bp
ITR C. briggsae unc-119 tofu-6 promotor+gene GFP HA tofu-6 3'UTR ITR
5706bp
ITR C. briggsae unc-119 smc-4/erh-2 promotor erh-2 gene GFP OLLAS erh-2 3'UTR ITR
5405bp
B
DIC
PGL-1::GFP
3xFLAG::mCherry::IFE-3
Germline
Embryo
C
ERH-2::GFP::OLLAS
PID-3::mCherry::Myc
TOFU-6::GFP::HA
D
PID-1::mCherry::V5
PID-3::mCherry::Myc
TOFU-6::GFP::HA
ERH-2::GFP::OLLAS

Cordeiro_Rodrigues_FigS4

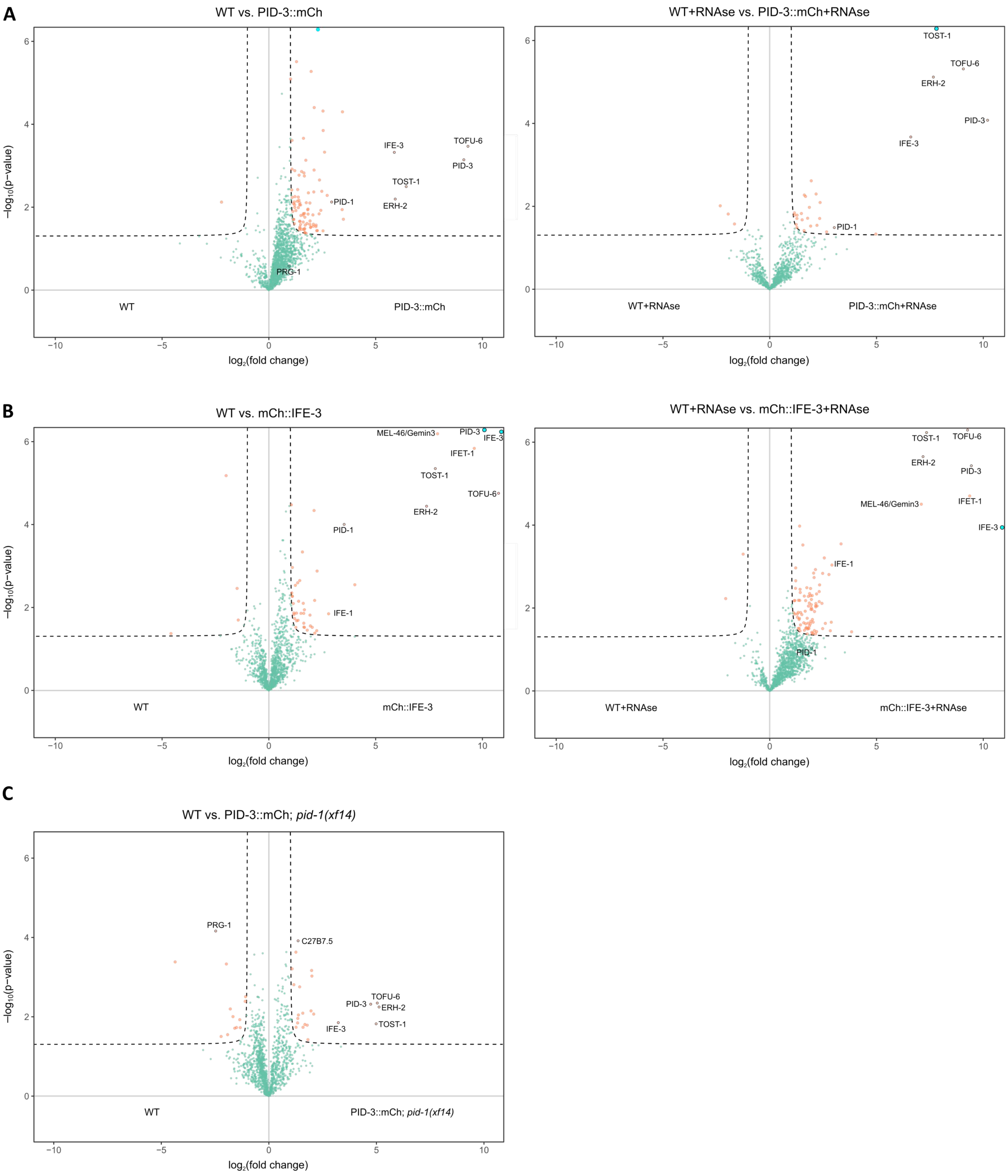

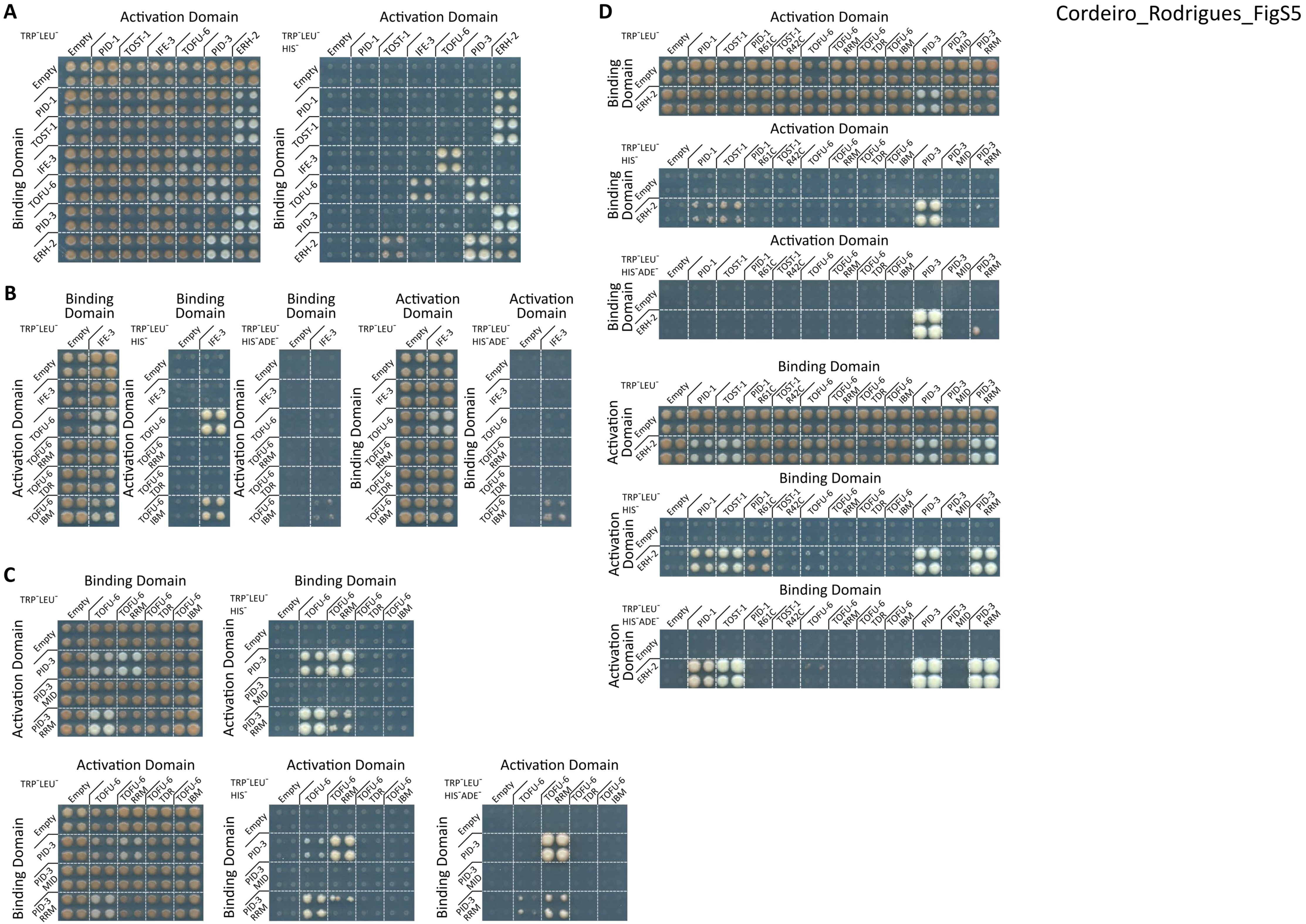
Cordeiro_Rodrigues_FigS5
A
Activation Domain
Binding Domain
TRP⁻LEU⁻
TRP⁻LEU⁻ HIS⁻
Empty
PID-1
TOST-1
IFE-3
TOFU-6
PID-3
ERH-2
B
Binding Domain
Activation Domain
TRP⁻LEU⁻ HIS⁻ADE⁻
TOFU-6 RRM
TOFU-6 TDR
TOFU-6 IBM
C
PID-3 MID
PID-3 RRM
D
PID-1 R61C
TOST-1 R42C

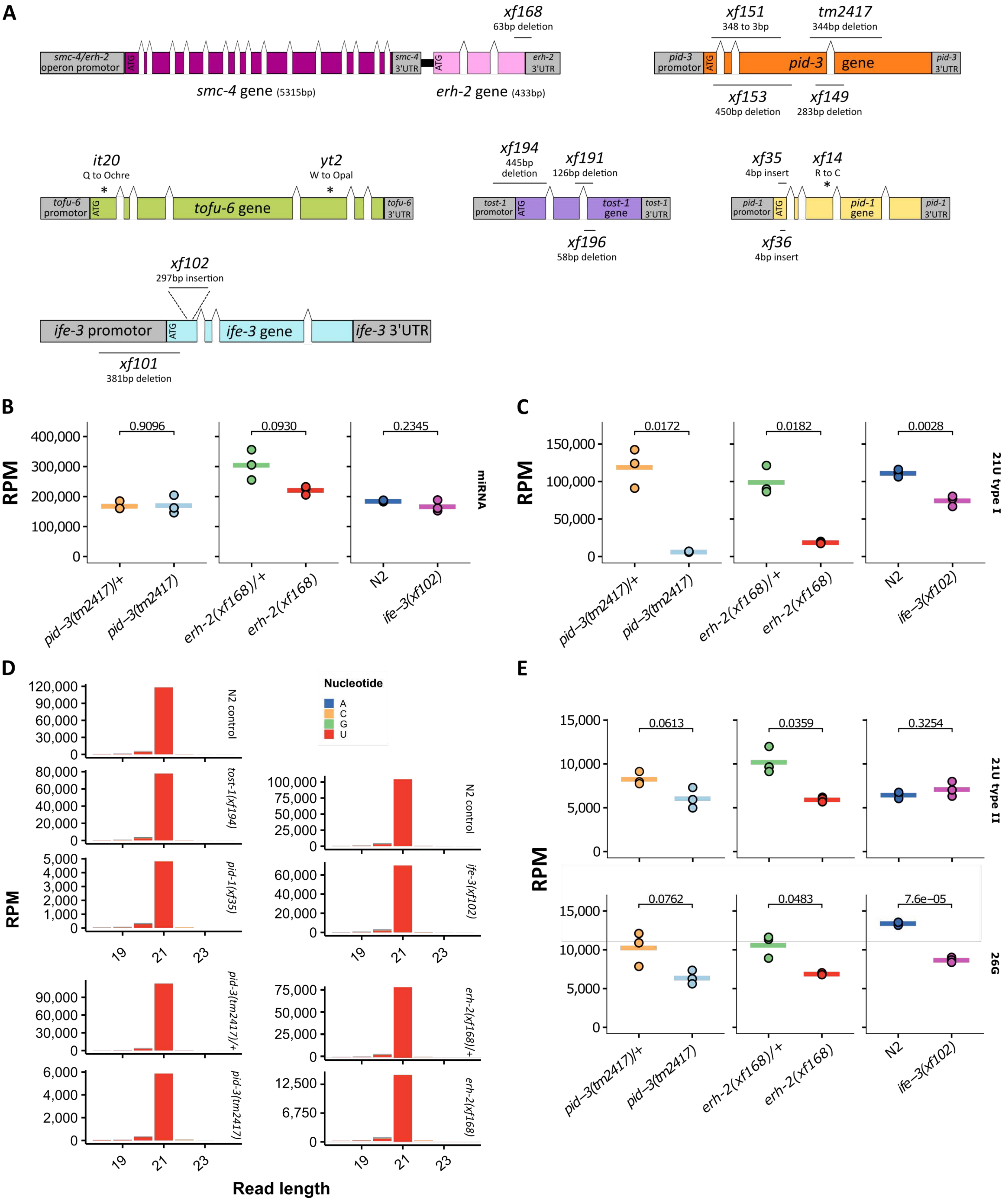
A
xf168
63bp deletion
smc-4/erh-2 operon promotor
ATG
smc-4 3'UTR
erh-2 3'UTR
smc-4 gene (5315bp)
erh-2 gene (433bp)
xf151
348 to 3bp
tm2417
344bp deletion
pid-3 promotor
pid-3 gene
pid-3 3'UTR
xf153
450bp deletion
xf149
283bp deletion
it20
Q to Ochre
yt2
W to Opal
tofu-6 promotor
tofu-6 gene
tofu-6 3'UTR
xf194
445bp deletion
xf191
126bp deletion
tost-1 promotor
tost-1 gene
tost-1 3'UTR
xf196
58bp deletion
xf35
4bp insert
xf14
R to C
pid-1 promotor
pid-1 gene
pid-1 3'UTR
xf36
4bp insert
xf102
297bp insertion
ife-3 promotor
ife-3 gene
ife-3 3'UTR
xf101
381bp deletion
B
RPM
0.9096
0.0930
0.2345
miRNA
pid–3(tm2417)/+
pid–3(tm2417)
erh–2(xf168)/+
erh–2(xf168)
N2
ife–3(xf102)
C
0.0172
0.0182
0.0028
21U type I
D
Nucleotide
A
C
G
U
N2 control
tost-1(xf194)
pid-1(xf35)
ife-3(xf102)
pid-3(tm2417)/+
erh-2(xf168)/+
pid-3(tm2417)
erh-2(xf168)
Read length
E
0.0613
0.0359
0.3254
21U type II
0.0762
0.0483
7.6e–05
26G

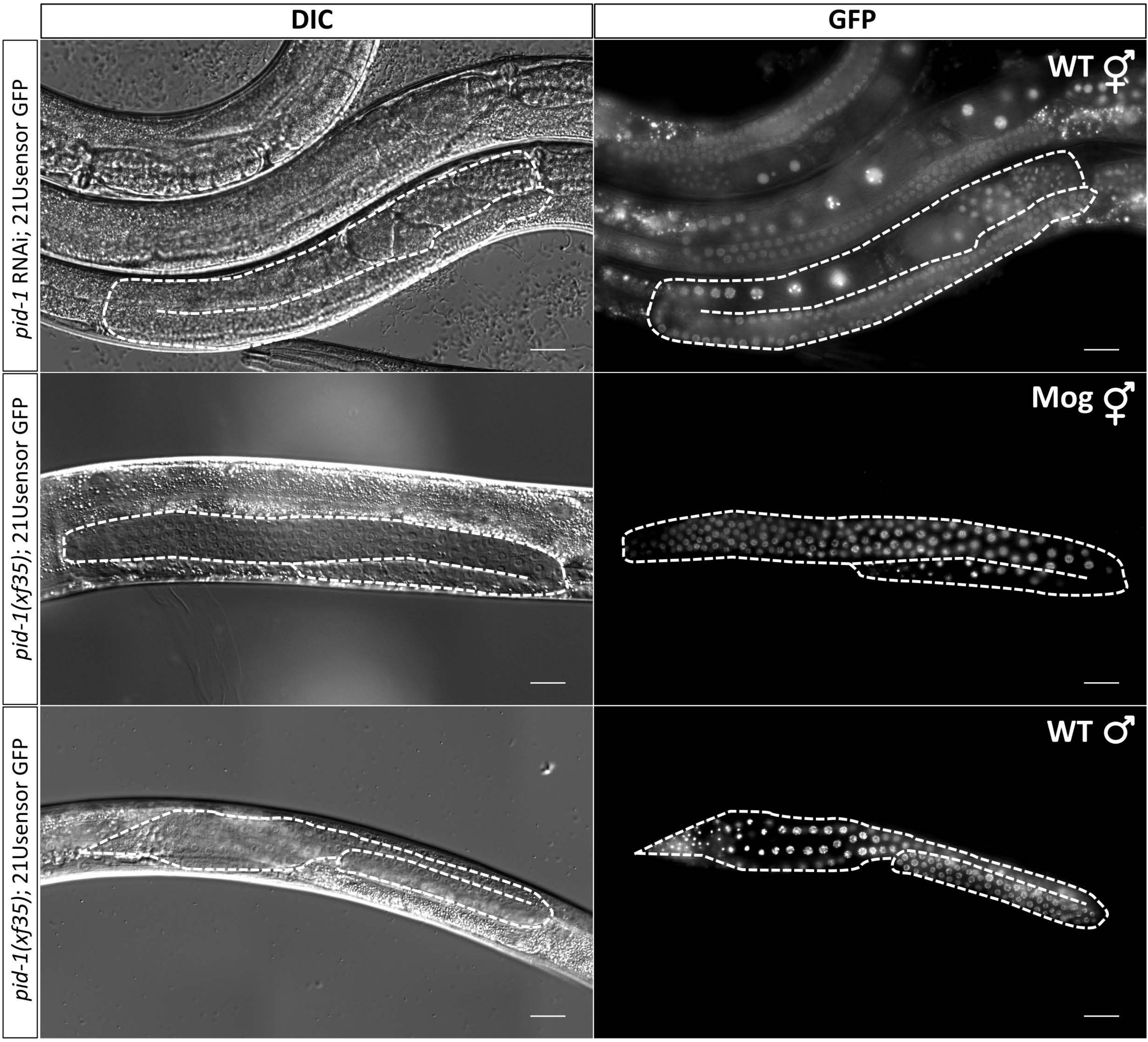
DIC
GFP
pid-1 RNAi; 21Usensor GFP
pid-1(xf35); 21Usensor GFP
pid-1(xf35); 21Usensor GFP
WT ⚥
Mog ⚥
WT ♂

Cordeiro_Rodrigues_FigS8

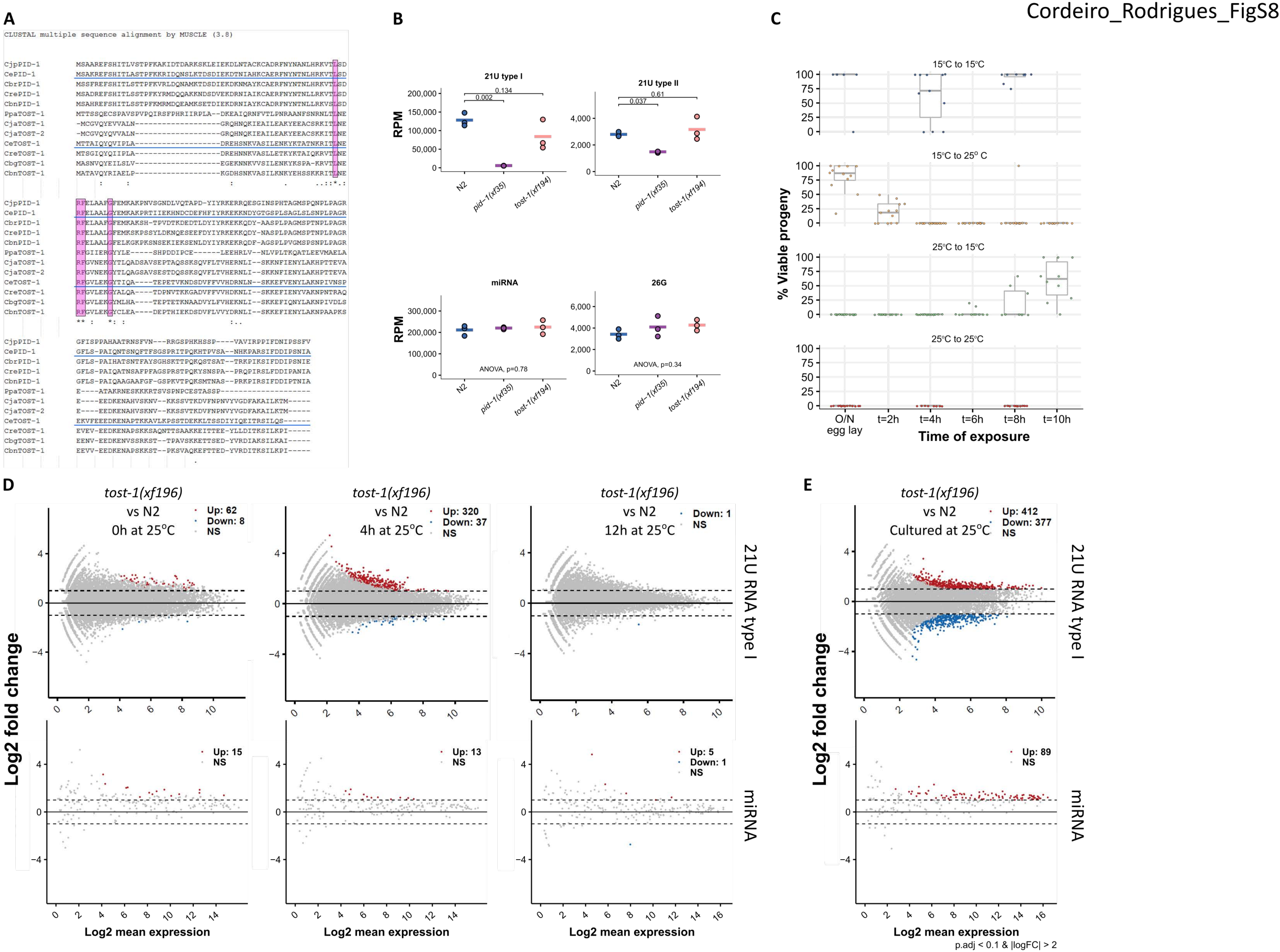

Cordeiro_Rodrigues_FigS9

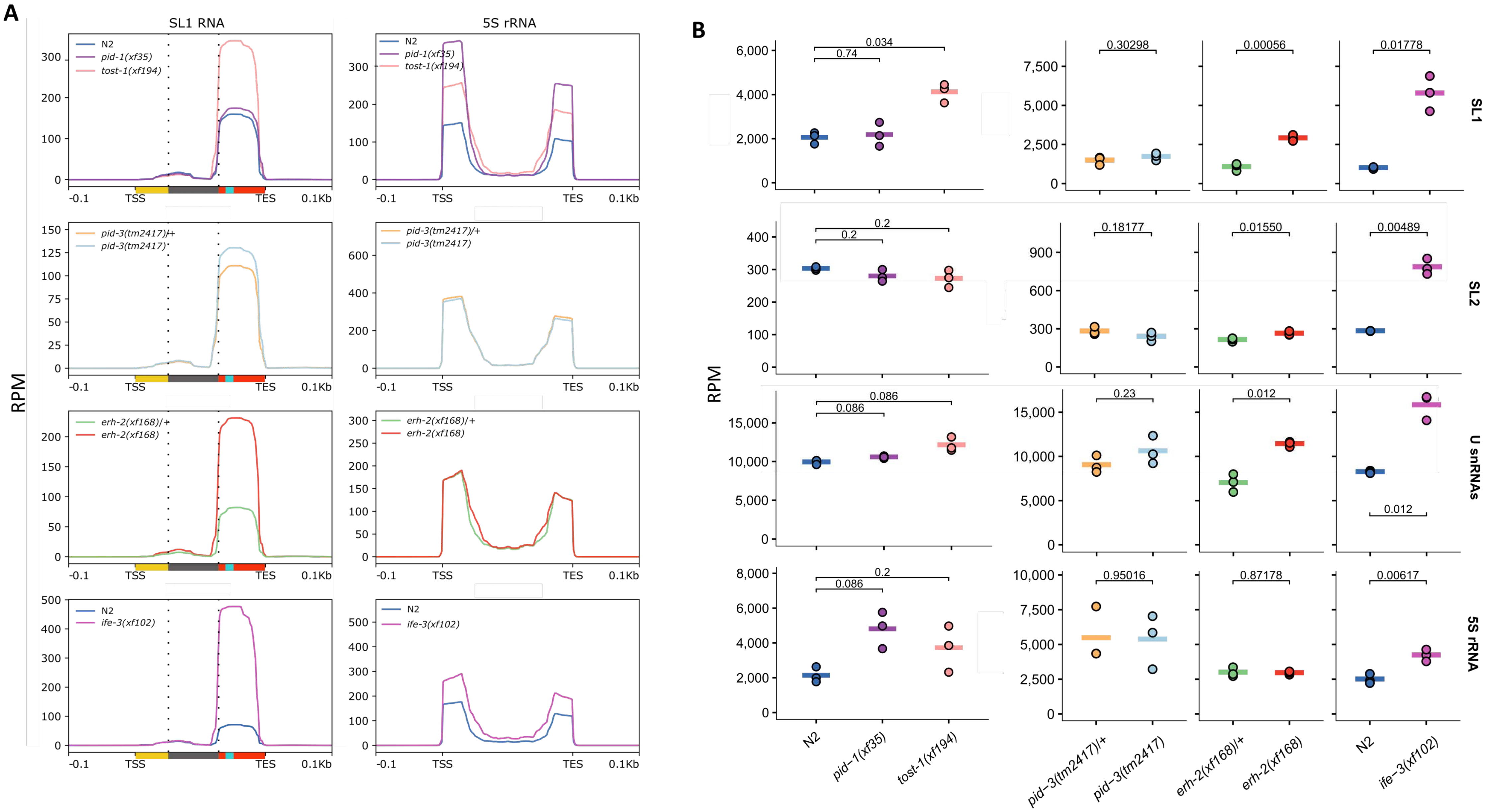

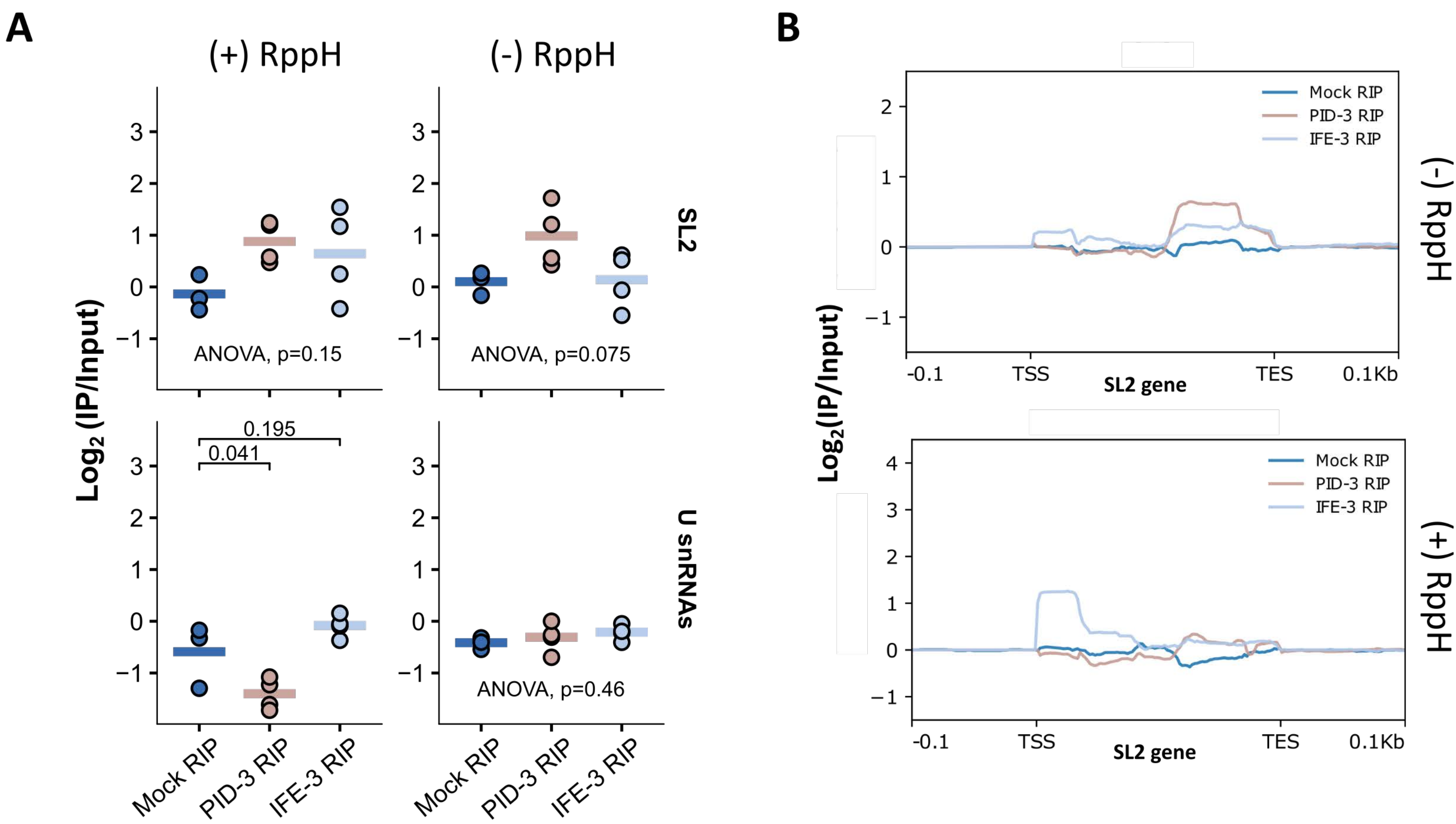
A
(+) RppH
(-) RppH
Log2 (IP/Input)
SL2
U snRNAs
ANOVA, p=0.15
ANOVA, p=0.075
ANOVA, p=0.46
0.195
0.041
Mock RIP
PID-3 RIP
IFE-3 RIP
B
Log2(IP/Input)
(-) RppH
(+) RppH
TSS
TES
-0.1
0.1Kb
SL2 gene

	Replicates	Pearson correlation coefficient 1 vs 2	1 vs 3	2 vs 3	1 vs 4	2 vs 4	3 vs 4
Sample	*pid-3(tm2417)/+*	0.963	0.973	0.989	n.a.	n.a.	n.a.
	pid-3(tm2417)	0.971	0.987	0.968	n.a.	n.a.	n.a.
	erh-2(xf168)/+	0.988	0.976	0.944	n.a.	n.a.	n.a.
	erh-2(xf168)	0.997	0.996	0.996	n.a.	n.a.	n.a.
	N2 - control for *ife-3(xf102)*	0.999	0.998	0.998	n.a.	n.a.	n.a.
	ife-3(xf102)	0.978	0.959	0.994	n.a.	n.a.	n.a.
	N2 - control for *pid-1(xf35)* and *tost-1(xf194)*	0.996	0.992	0.981	n.a.	n.a.	n.a.
	tost-1(xf194)	0.969	0.985	0.927	n.a.	n.a.	n.a.
	pid-1(xf35)	0.998	0.994	0.998	n.a.	n.a.	n.a.
	N2 timelapse (0h)	0.99	0.991	0.998	n.a.	n.a.	n.a.
	N2 timelapse (4h)	0.998	0.97	0.97	n.a.	n.a.	n.a.
	N2 timelapse (12h)	0.989	0.934	0.922	n.a.	n.a.	n.a.
	tost-1(xf196) timelapse (0h)	0.877	0.938	0.98	n.a.	n.a.	n.a.
	tost-1(xf196) timelapse (4h)	0.947	0.965	0.97	n.a.	n.a.	n.a.
	tost-1(xf196) timelapse (12h)	0.765	0.746	0.997	n.a.	n.a.	n.a.
	N2 cultured at 25C	0.997	0.997	0.999	n.a.	n.a.	n.a.
	tost-1(xf196) cultured at 25C	0.996	0.993	0.994	n.a.	n.a.	n.a.
	N2 inputs untreated	n.a.	n.a.	0.999	n.a.	0.999	0.999
	N2 RIP untreated	n.a.	n.a.	1	n.a.	0.999	0.999
	PID-3::mCherry inputs untreated	0.998	0.997	0.998	0.998	0.998	1
	PID-3::mCherry RIP untreated	0.999	0.97	0.979	0.962	0.974	0.999
	IFE-3::mCherry inputs untreated	0.997	0.995	0.998	0.994	0.998	0.999
	IFE-3::mCherry RIP untreated	0.997	0.962	0.975	0.971	0.983	0.999
	N2 inputs RppH treated	n.a.	n.a.	0.998	n.a.	0.997	0.999
	N2 RIP RppH treated	n.a.	n.a.	0.998	n.a.	0.999	0.998
	PID-3::mCherry inputs RppH treated	0.998	0.995	0.991	0.999	0.997	0.992
	PID-3::mCherry RIP RppH treated	0.997	0.956	0.975	0.951	0.97	0.998
	IFE-3::mCherry inputs RppH treated	0.992	0.991	0.999	0.986	0.998	0.998
	IFE-3::mCherry RIP RppH treated	0.999	0.967	0.967	0.972	0.972	0.999
	N2 - control for *pid-1(xf35)* CIP+RppH treated	0.995	0.985	0.99	n.a.	n.a.	n.a.
	pid-1(xf35) CIP+RppH treated	0.999	0.972	0.968	n.a.	n.a.	n.a.

Table S3

Gene	Allele	Mutation Type	21U RNA Presence	Terminal Phenotypes	Ratio (%)
ife-3	*xf101*	Start Codon Loss	n.d.	Mog/Mel	n.d.
	xf102	Frameshift	-	Mog/Mel	n.d.
	RNAi	n.a.	n.d.	Mog/Mel	47/53#
pid-3	*tm2417*	Frameshift	--	Mel	100
	xf149	Frameshift	n.d.	Mel	100
	xf151	Inframe Deletion	n.d.	Mel	100
	xf153	Frameshift	n.d.	Mel	100
tofu-6	*it20*	Nonsense	n.d.	Mel	100
	yt2	Nonsense	n.d.	Mel	100
	RNAi	n.a.	--*	Mel	n.d.
tost-1	*xf191*	Frameshift	n.d.	Viable	n.d.
	xf194	Start Codon Loss	+	Mel	100
	xf196	Splice Site Loss	n.d.	(TS) Mel	100 (25°C)
pid-1	*xf14*	Missense	--	n.d.	n.d.
	xf35	Frameshift	--	Mog	<1
	xf36	Frameshift	--	n.d.	n.d.
erh-2	*xf168*	Stop Codon Loss	--	Mel	100
