# Supplementary figures and images for: PETISCO is a novel protein complex required for 21U RNA biogenesis and embryonic viability
Source: Genes Dev. 2019 Jul 1;33(13-14):857–70. doi: 10.1101/gad.322446.118 (PMC6601512; doi:10.1101/gad.322446.118)

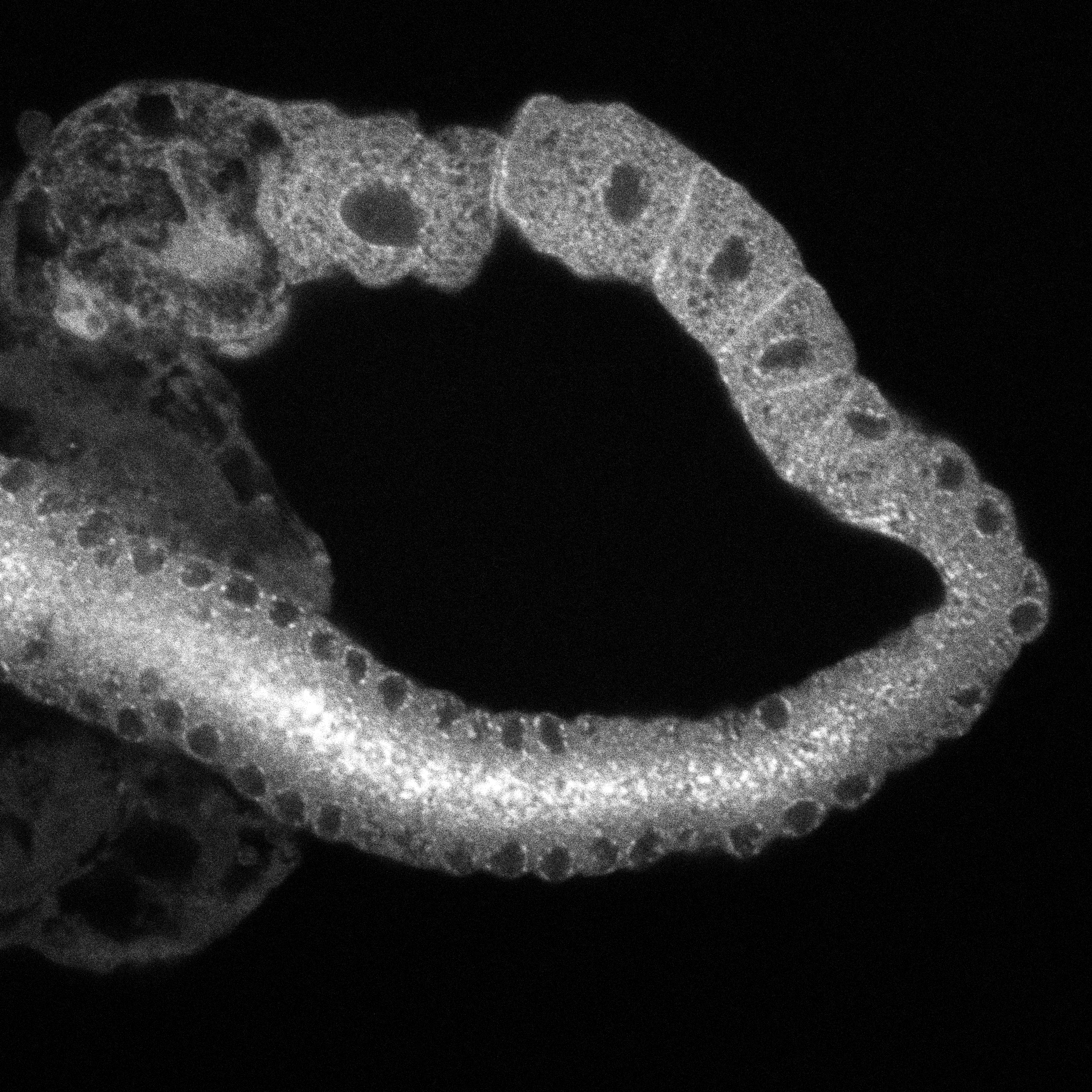

Supplement: Supplemental Material [file supp_gad.322446.118_Supplemental_Source_material.zip › Fig 1 Source material/Panel B/IFE-3 TOFU-6 - IFE-3 mCherry.tif]

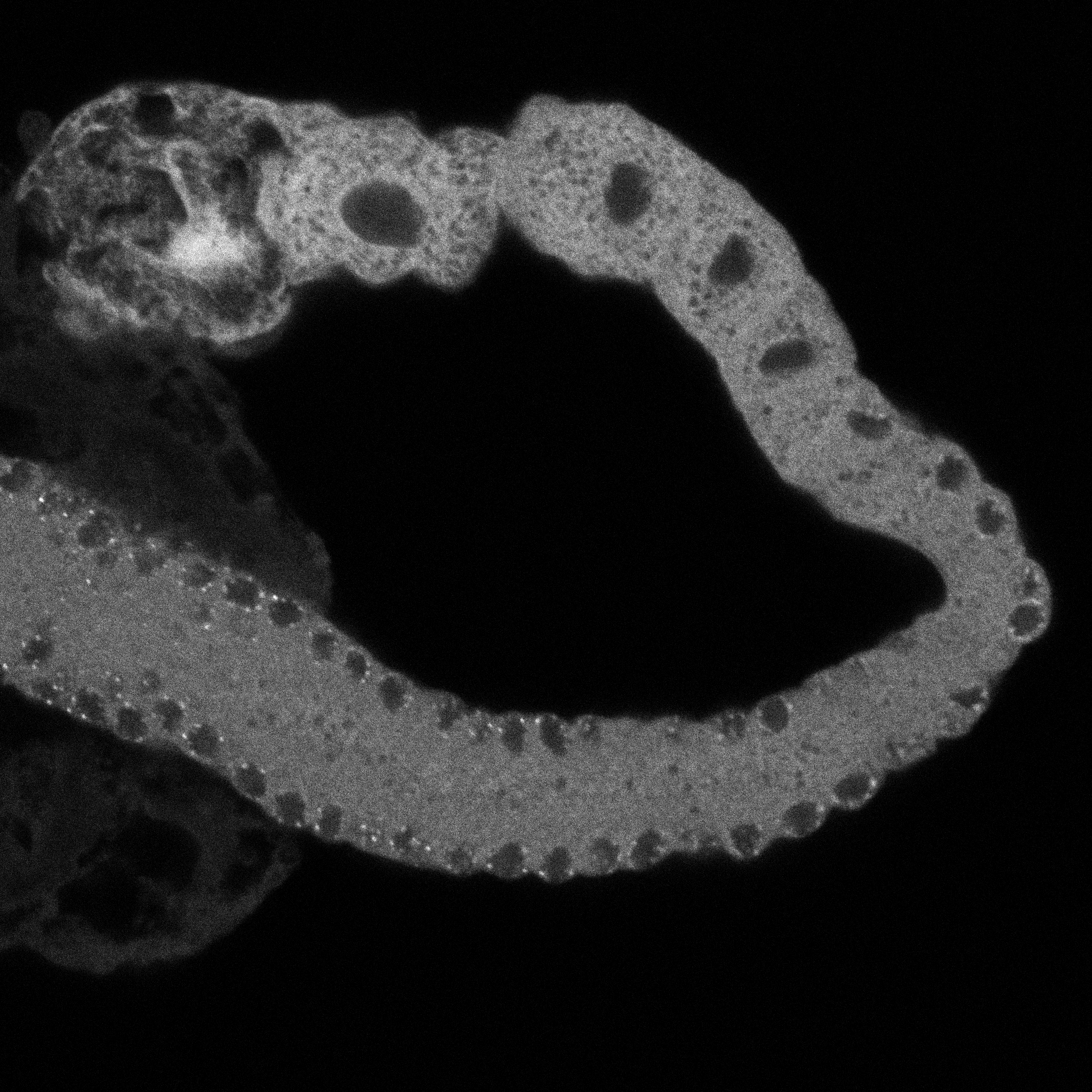

Supplement: Supplemental Material [file supp_gad.322446.118_Supplemental_Source_material.zip › Fig 1 Source material/Panel B/IFE-3 TOFU-6 - TOFU-6 GFP.tif]

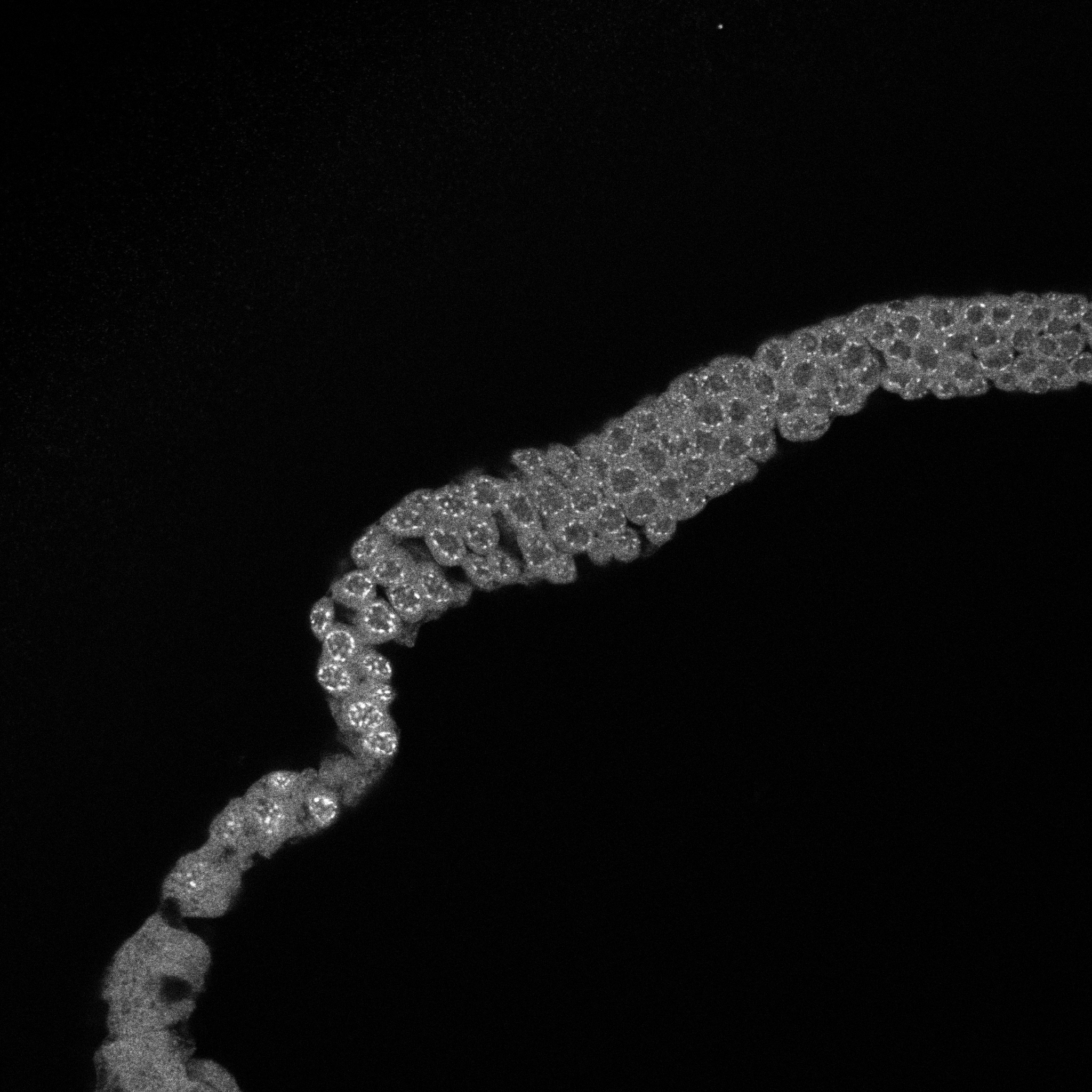

Supplement: Supplemental Material [file supp_gad.322446.118_Supplemental_Source_material.zip › Fig 1 Source material/Panel B/PID-3 PGL-1 - PGL1 GFP.tif]

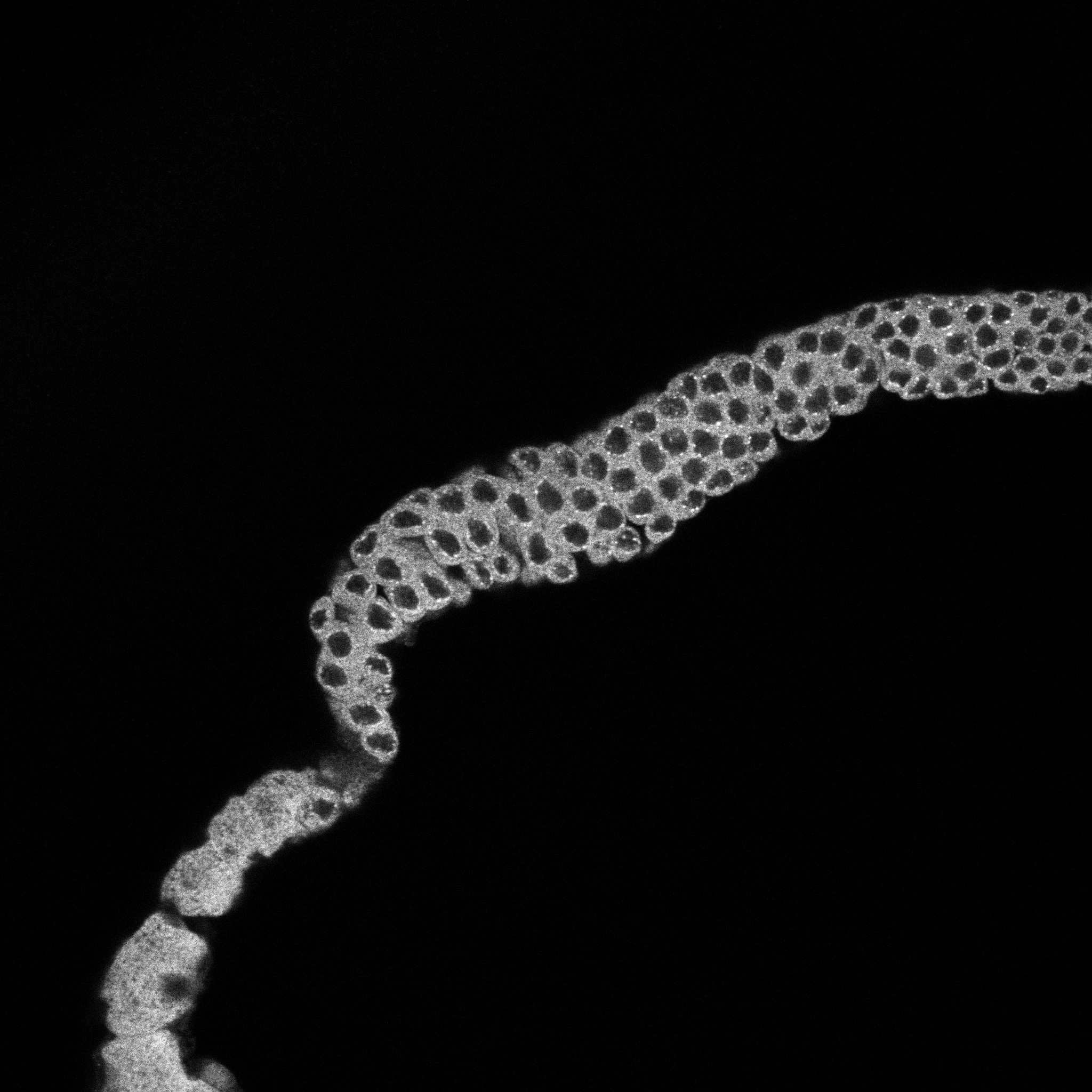

Supplement: Supplemental Material [file supp_gad.322446.118_Supplemental_Source_material.zip › Fig 1 Source material/Panel B/PID-3 PGL-1 - PID3mcherry.tif]

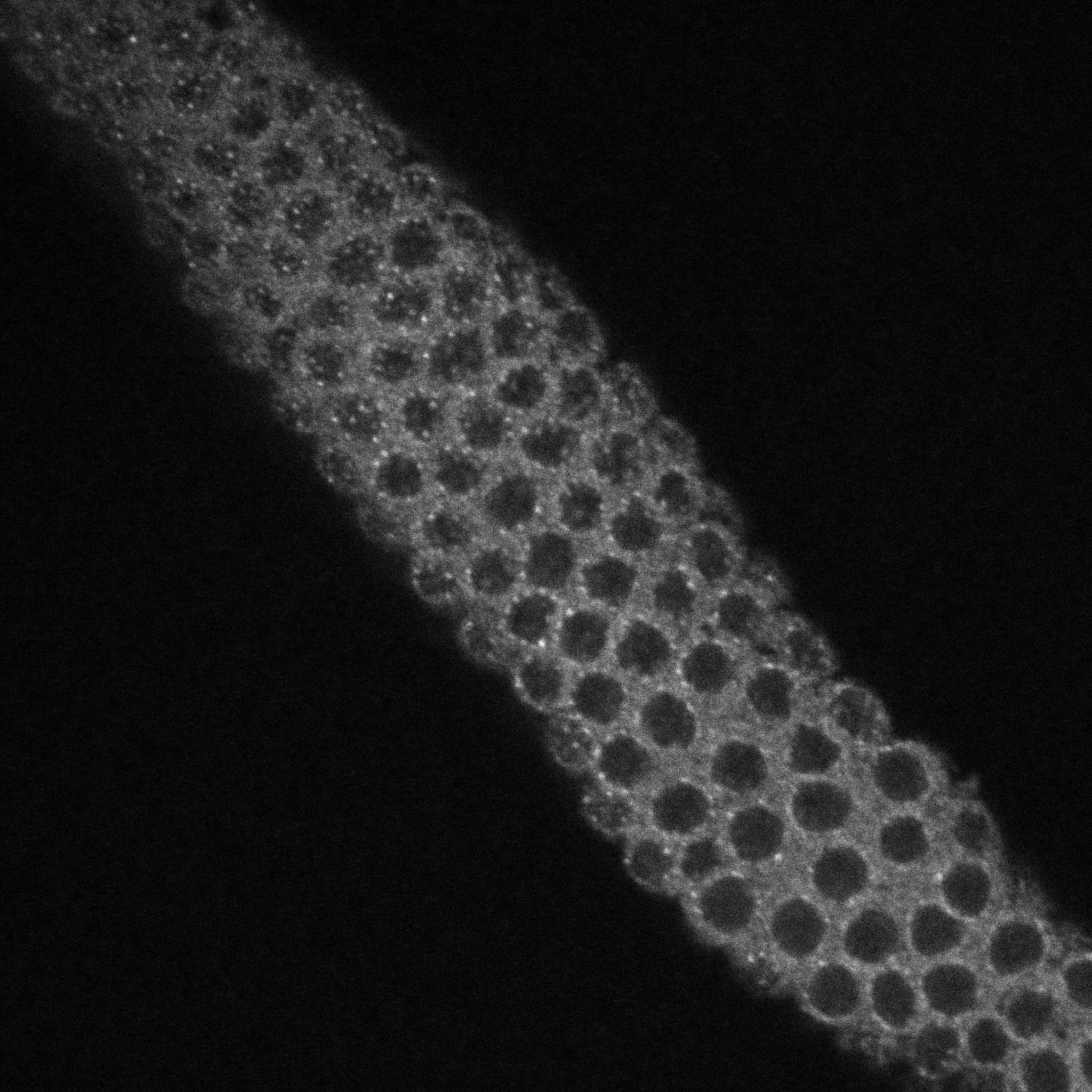

Supplement: Supplemental Material [file supp_gad.322446.118_Supplemental_Source_material.zip › Fig 1 Source material/Panel B/PID-3 TOFU-6 - PID-3 mCherry.tif]

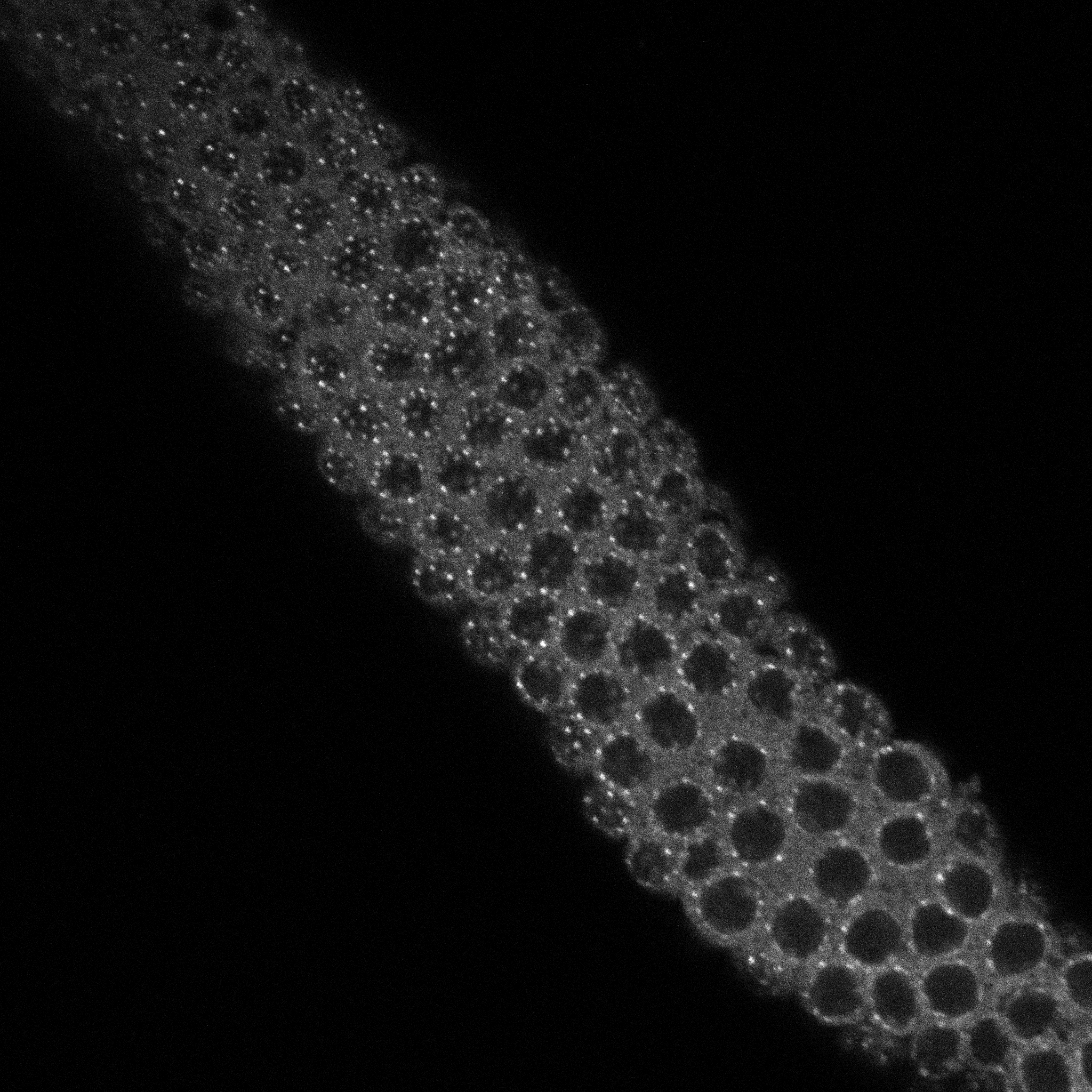

Supplement: Supplemental Material [file supp_gad.322446.118_Supplemental_Source_material.zip › Fig 1 Source material/Panel B/PID-3 TOFU-6 - TOFU-6 GFP.tif]

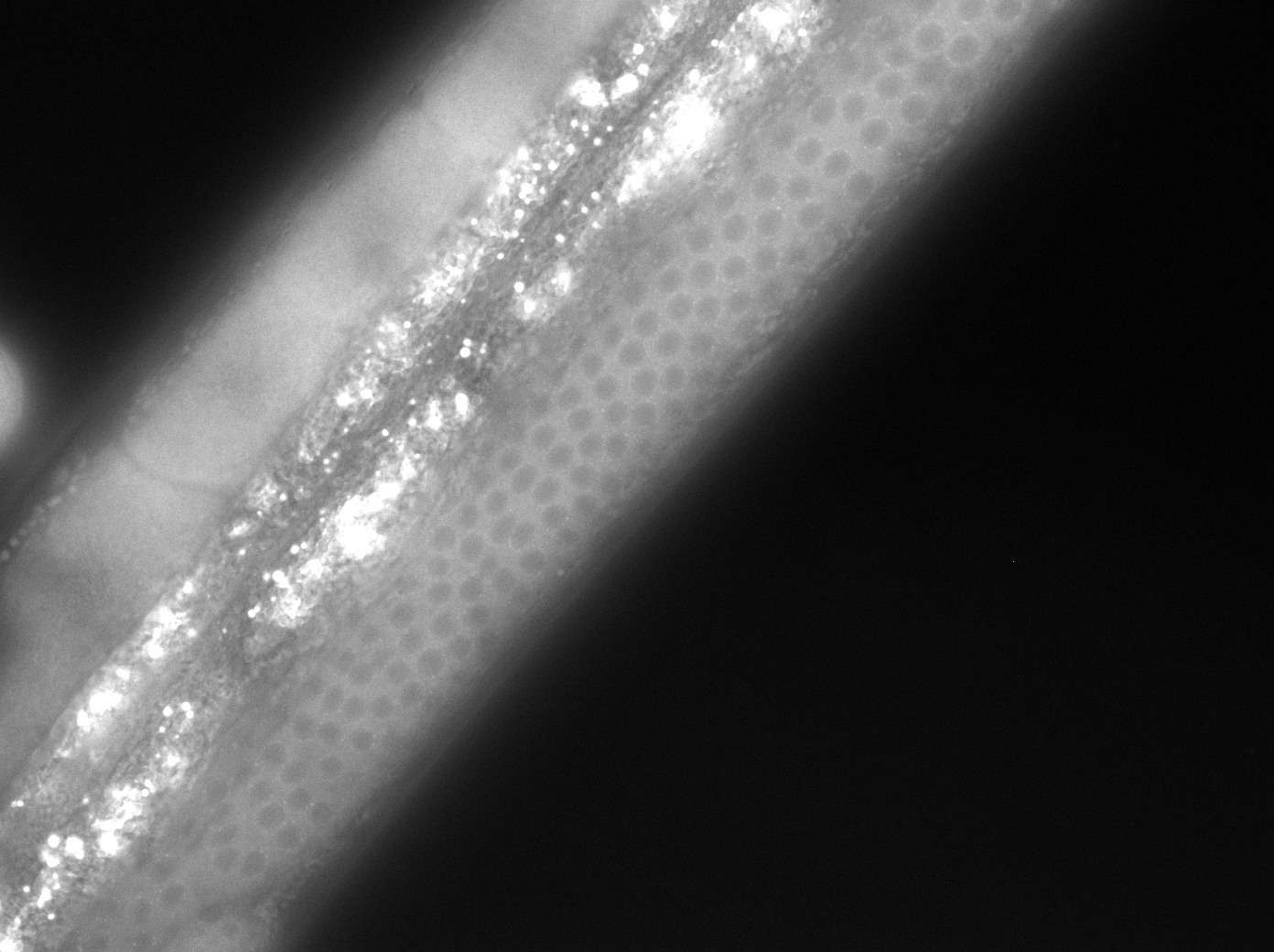

Supplement: Supplemental Material [file supp_gad.322446.118_Supplemental_Source_material.zip › Fig 1 Source material/Panel C/ERH-2 GFP.tif]

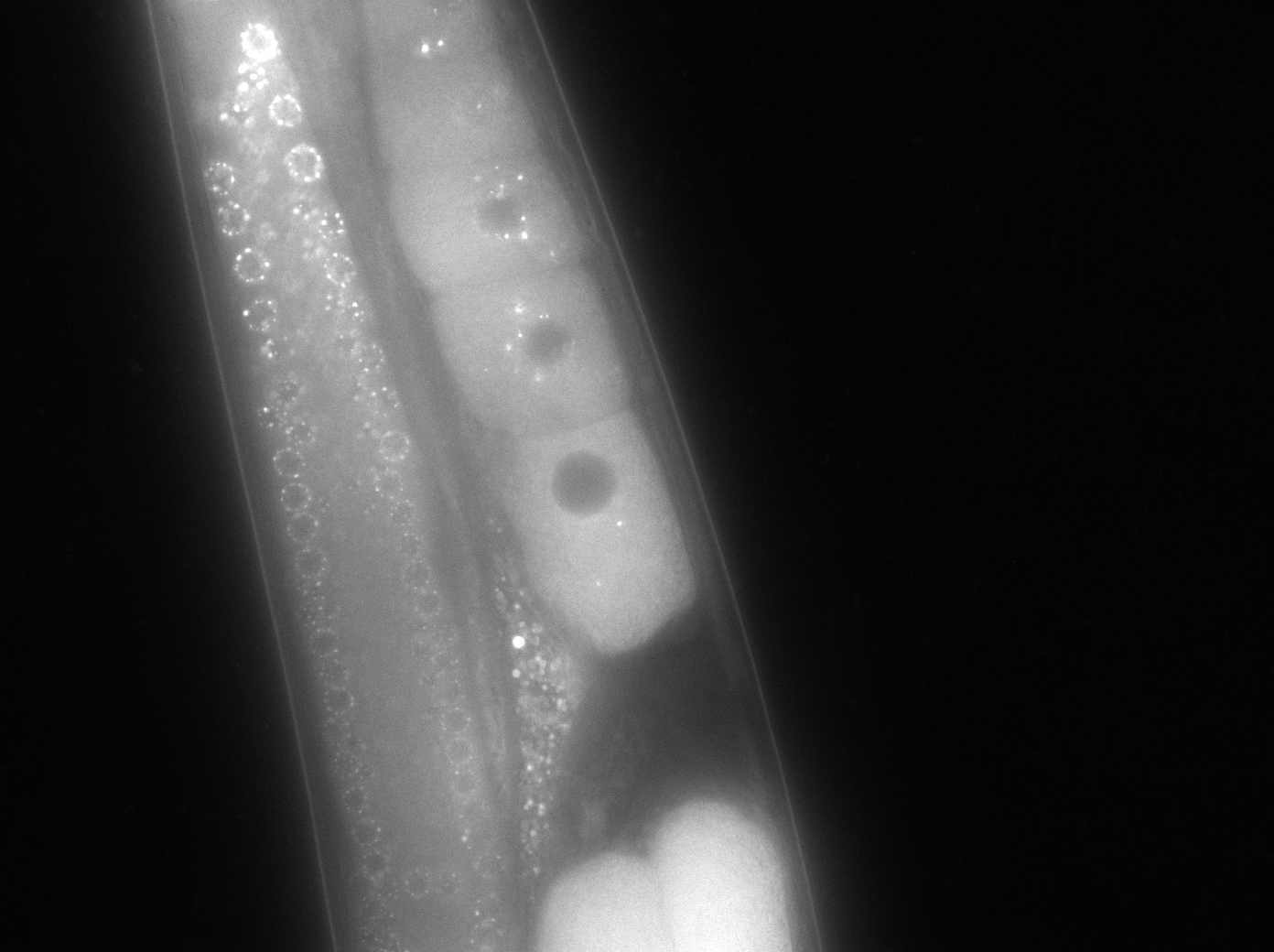

Supplement: Supplemental Material [file supp_gad.322446.118_Supplemental_Source_material.zip › Fig 1 Source material/Panel C/PID1 PGL1 - PGL1 GFP.tif]

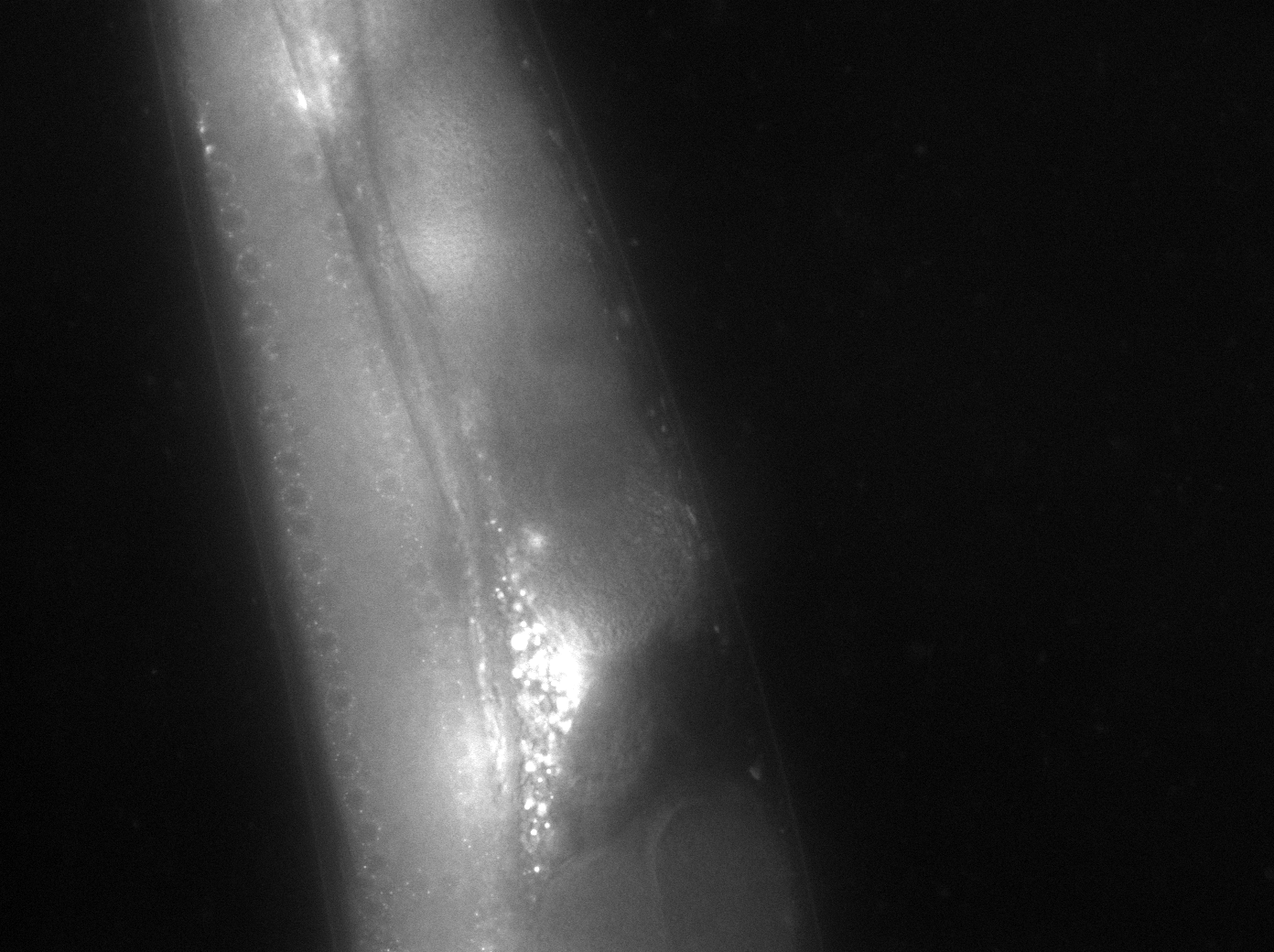

Supplement: Supplemental Material [file supp_gad.322446.118_Supplemental_Source_material.zip › Fig 1 Source material/Panel C/PID1 PGL1 - PID-1mCherry.tif]

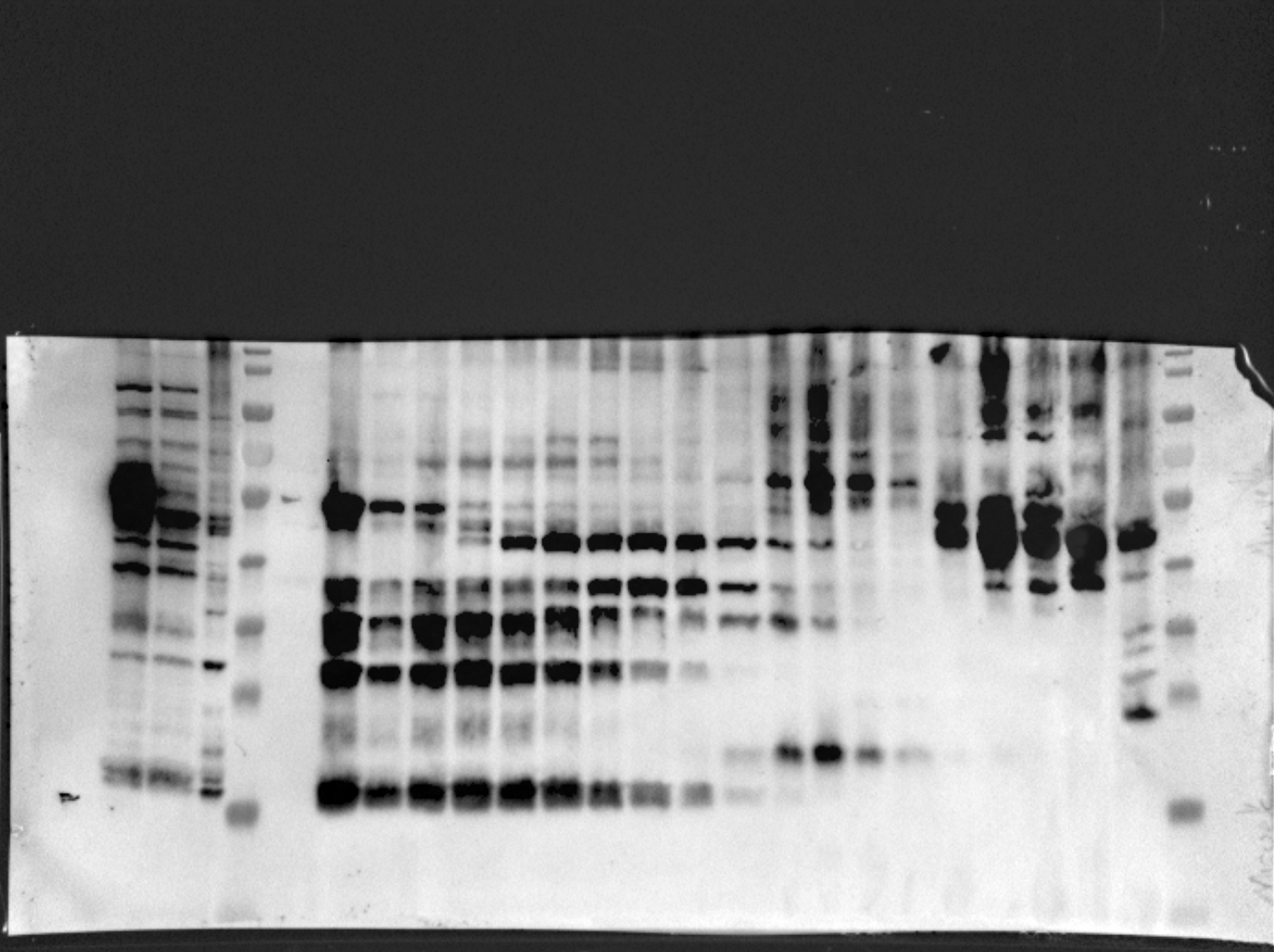

Supplement: Supplemental Material [file supp_gad.322446.118_Supplemental_Source_material.zip › Fig 2 Source material/Panel E/3xFLAgRRF-3 chromatography WB anti PID-1 with marker.tif]

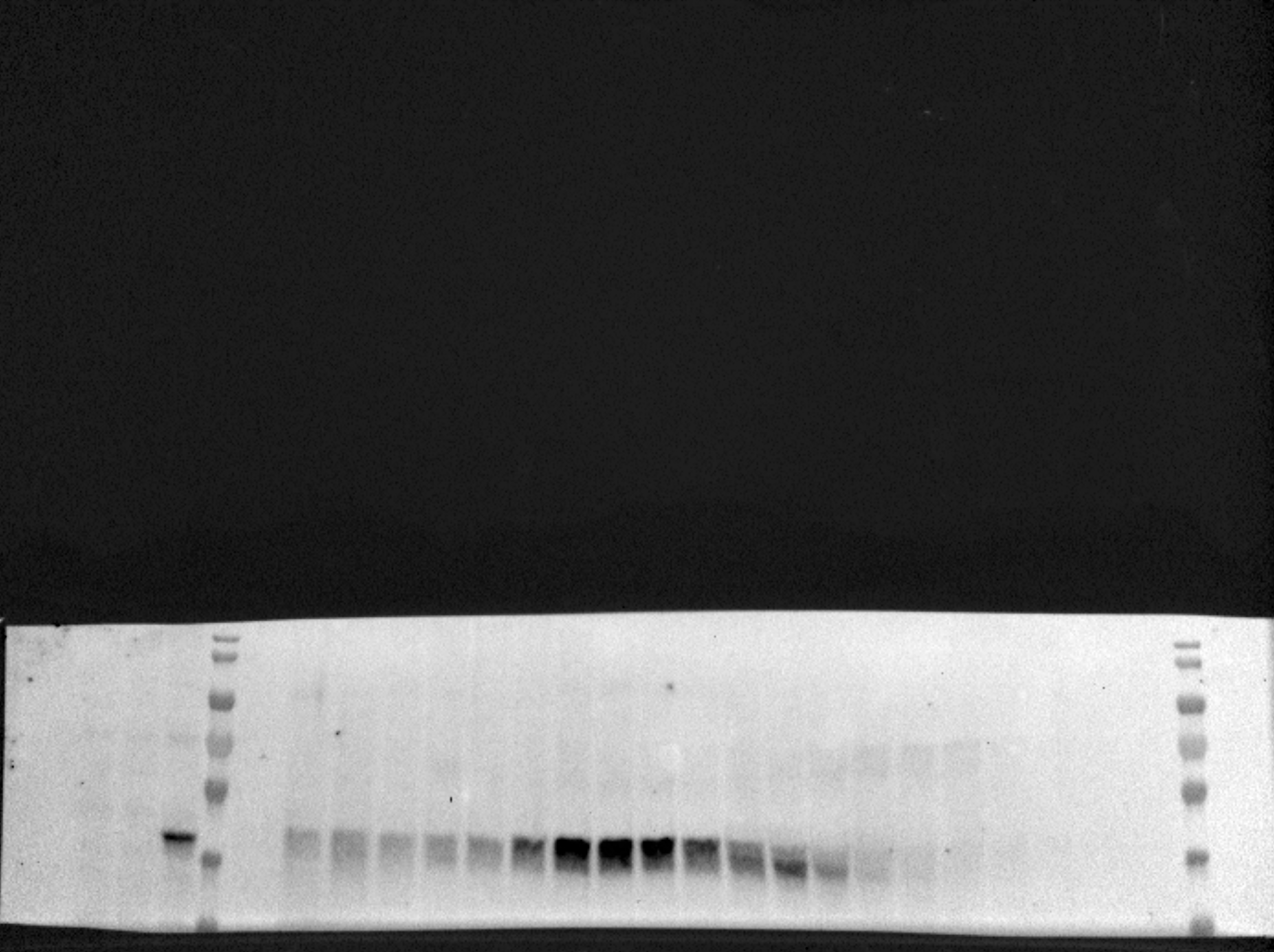

Supplement: Supplemental Material [file supp_gad.322446.118_Supplemental_Source_material.zip › Fig 2 Source material/Panel E/erh-2GFP chromatography WB anti GFP with marker.tif]

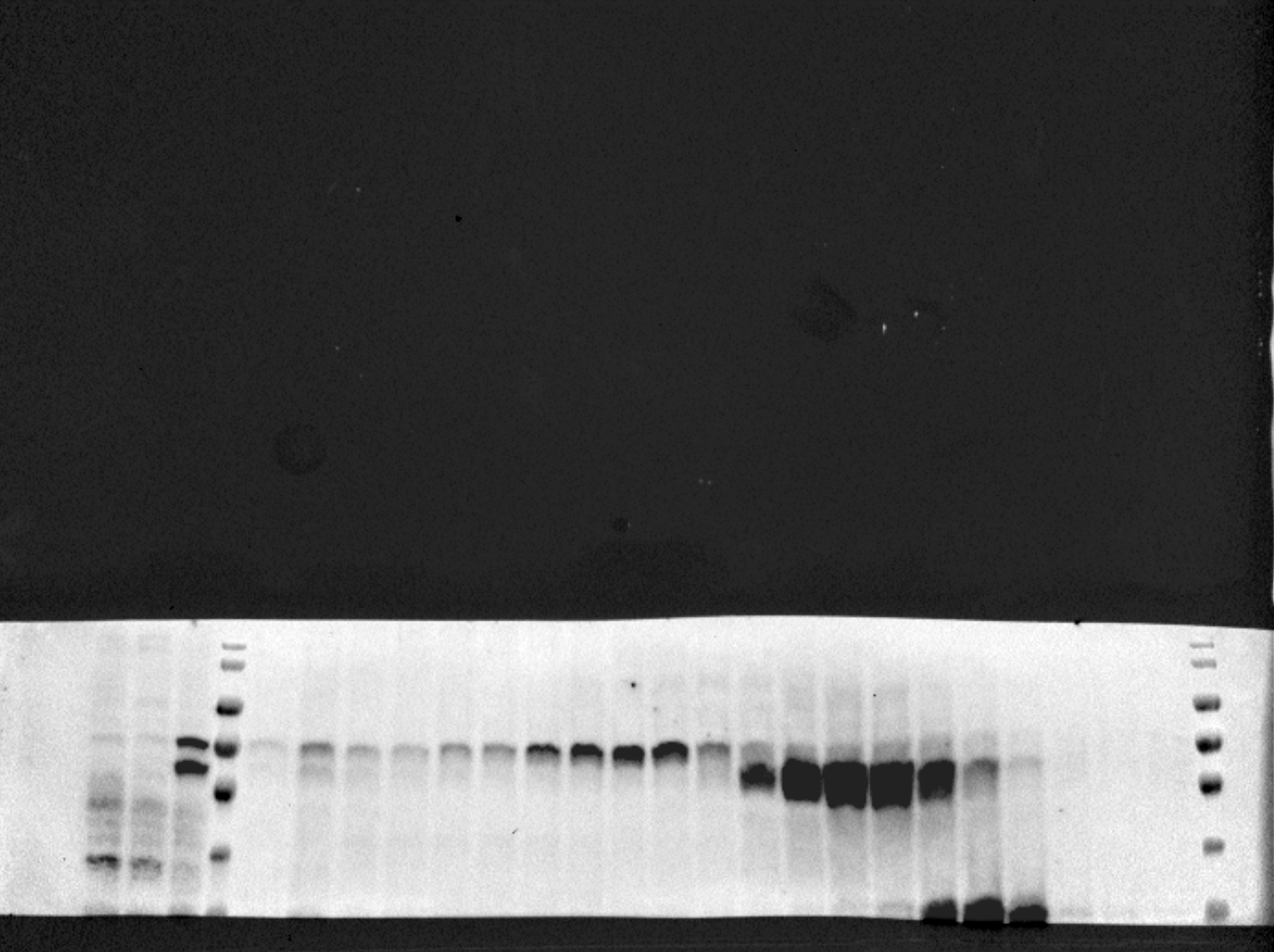

Supplement: Supplemental Material [file supp_gad.322446.118_Supplemental_Source_material.zip › Fig 2 Source material/Panel E/tofu-6GFP chromatography WB anti HA with marker.tif]

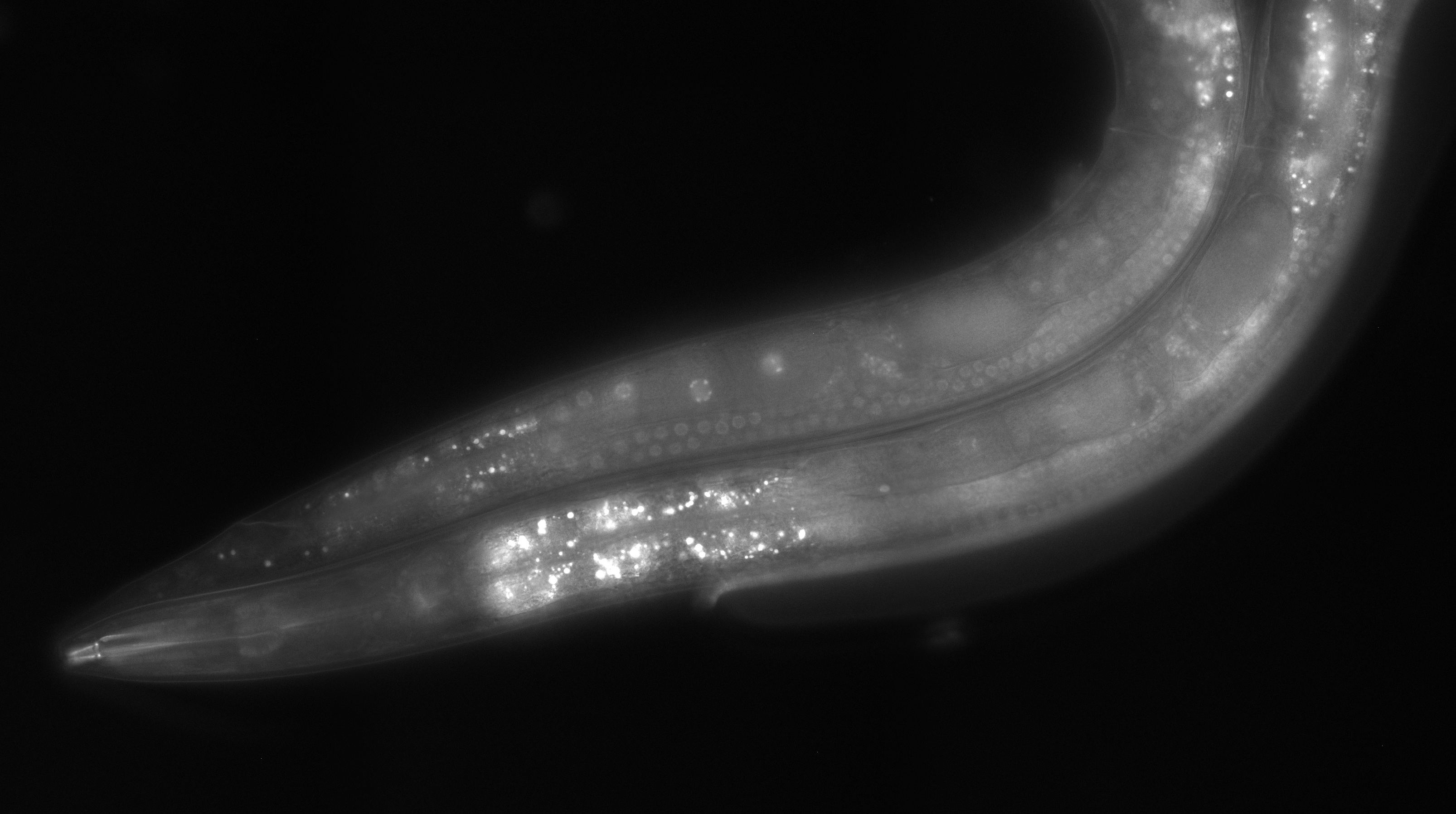

Supplement: Supplemental Material [file supp_gad.322446.118_Supplemental_Source_material.zip › Fig 4 Source material/Panel A/Empty vector.tif]

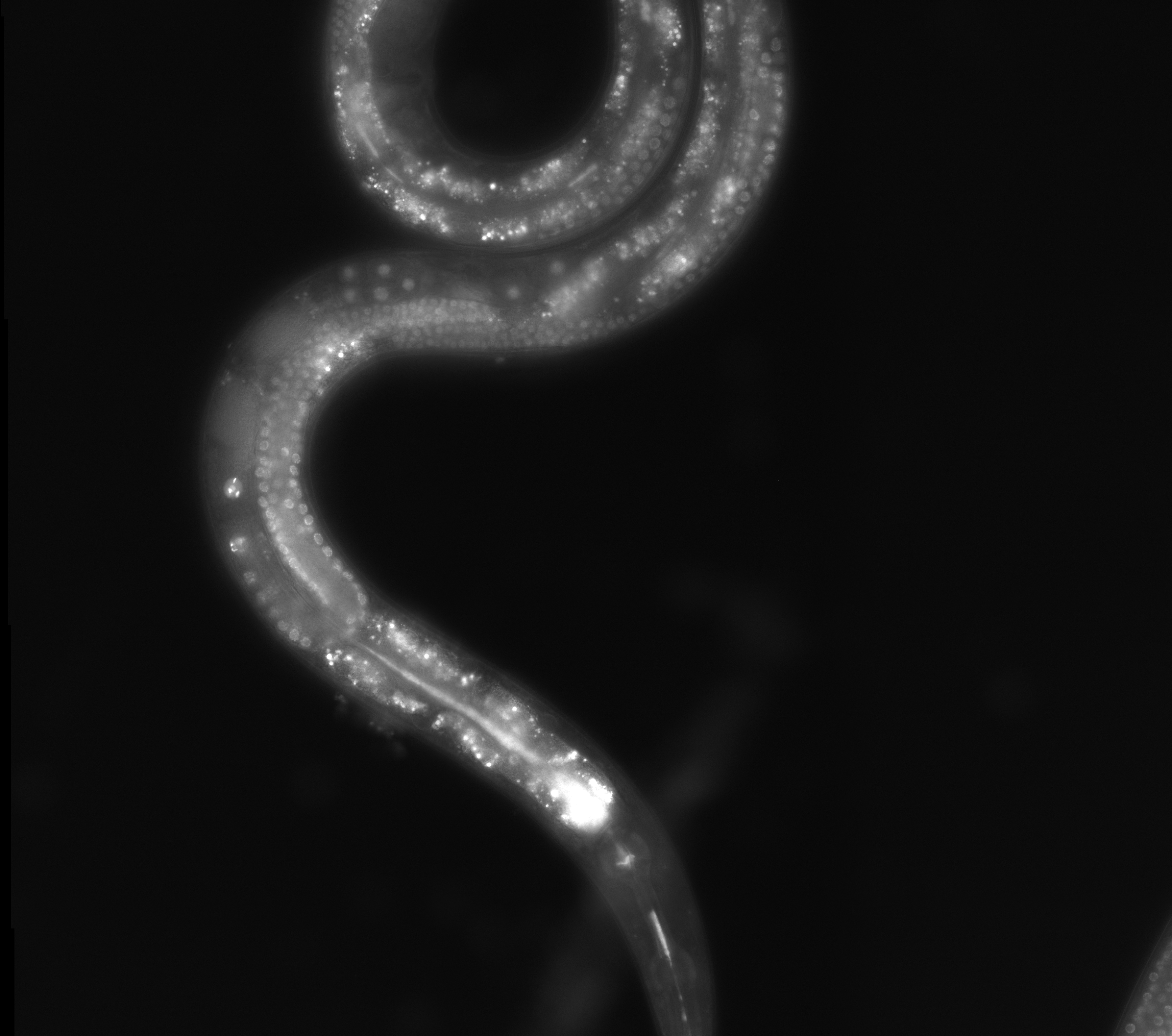

Supplement: Supplemental Material [file supp_gad.322446.118_Supplemental_Source_material.zip › Fig 4 Source material/Panel A/erh-2.tif]

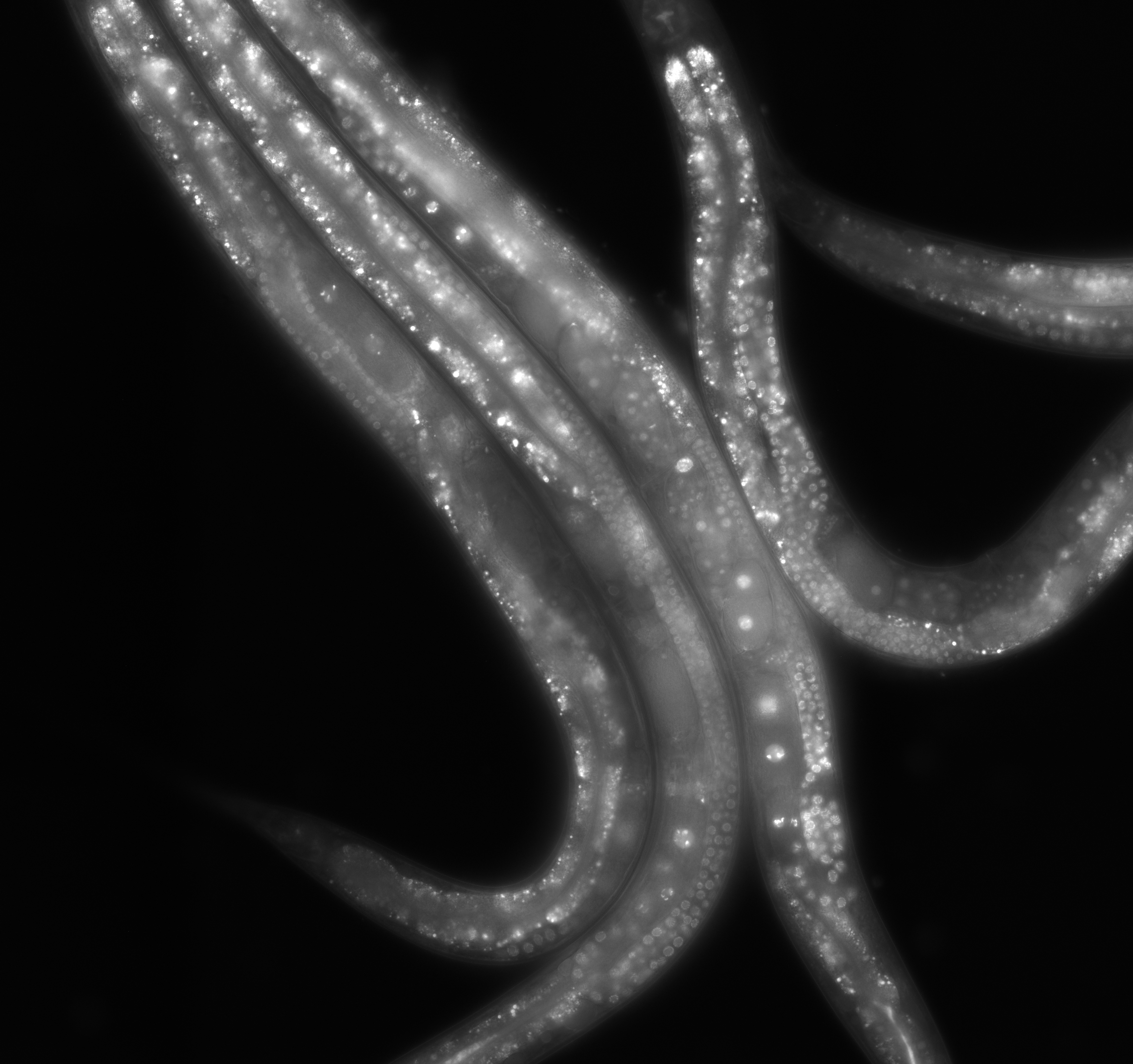

Supplement: Supplemental Material [file supp_gad.322446.118_Supplemental_Source_material.zip › Fig 4 Source material/Panel A/ife-3.tif]

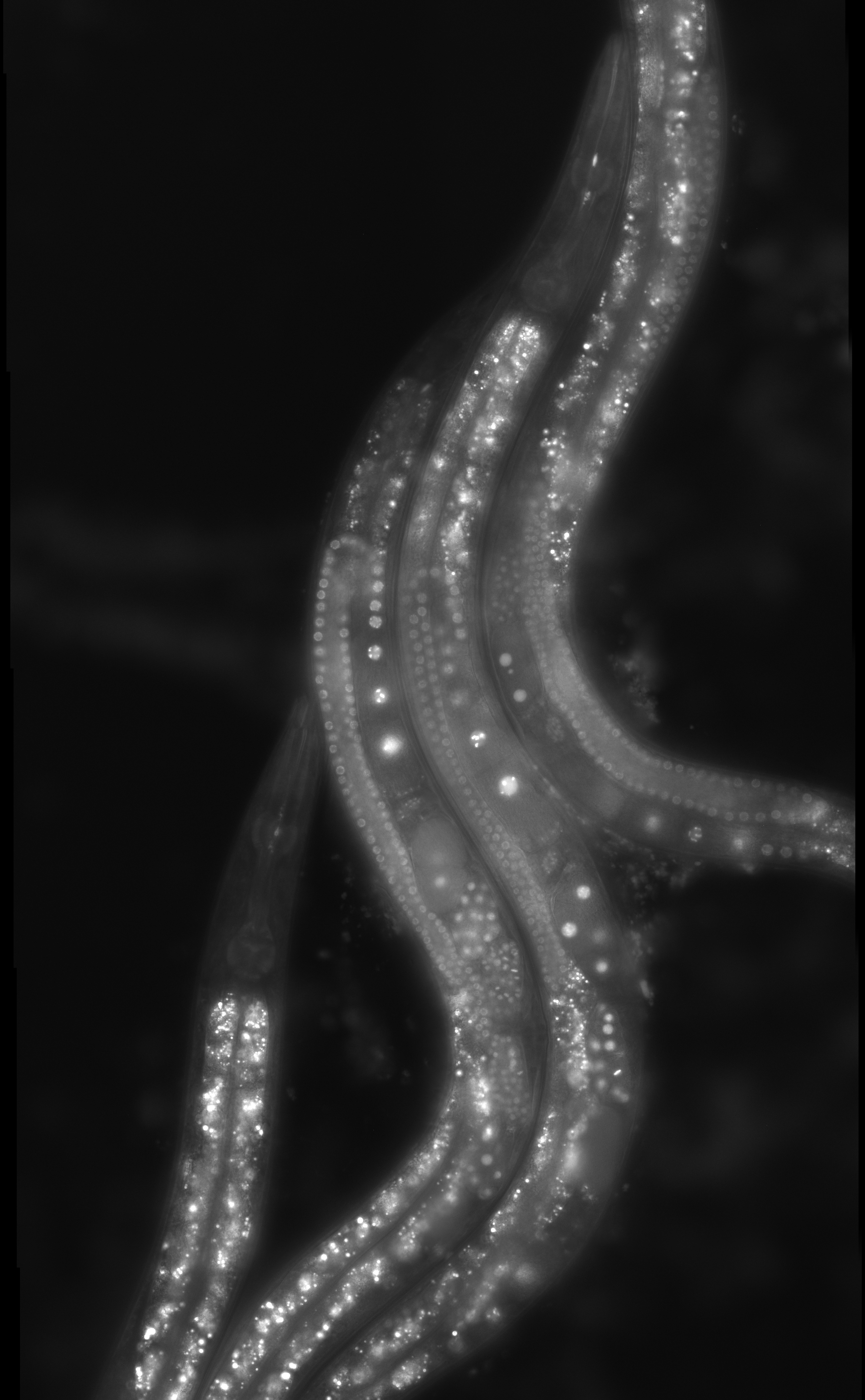

Supplement: Supplemental Material [file supp_gad.322446.118_Supplemental_Source_material.zip › Fig 4 Source material/Panel A/pid-1.tif]

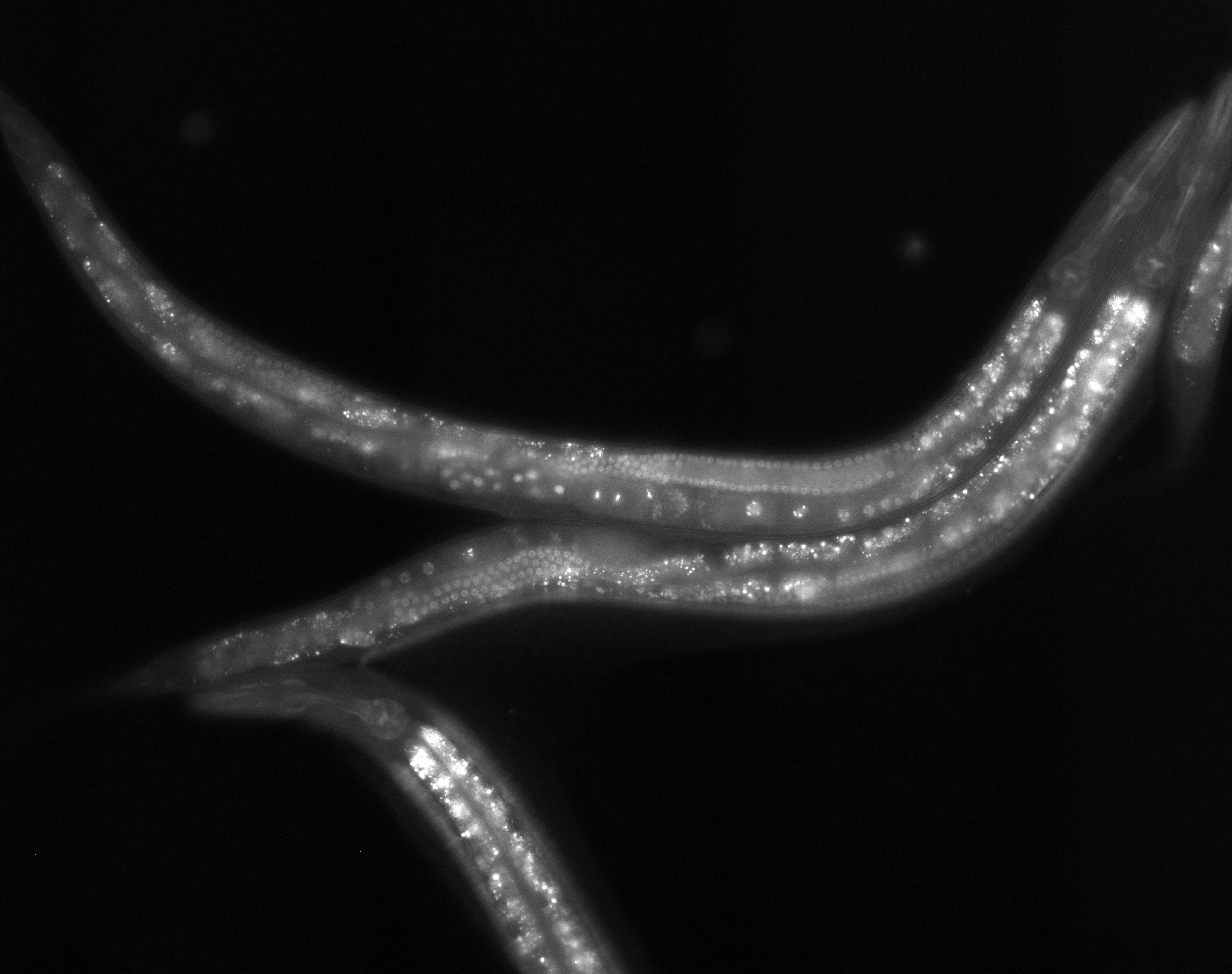

Supplement: Supplemental Material [file supp_gad.322446.118_Supplemental_Source_material.zip › Fig 4 Source material/Panel A/pid-3.tif]

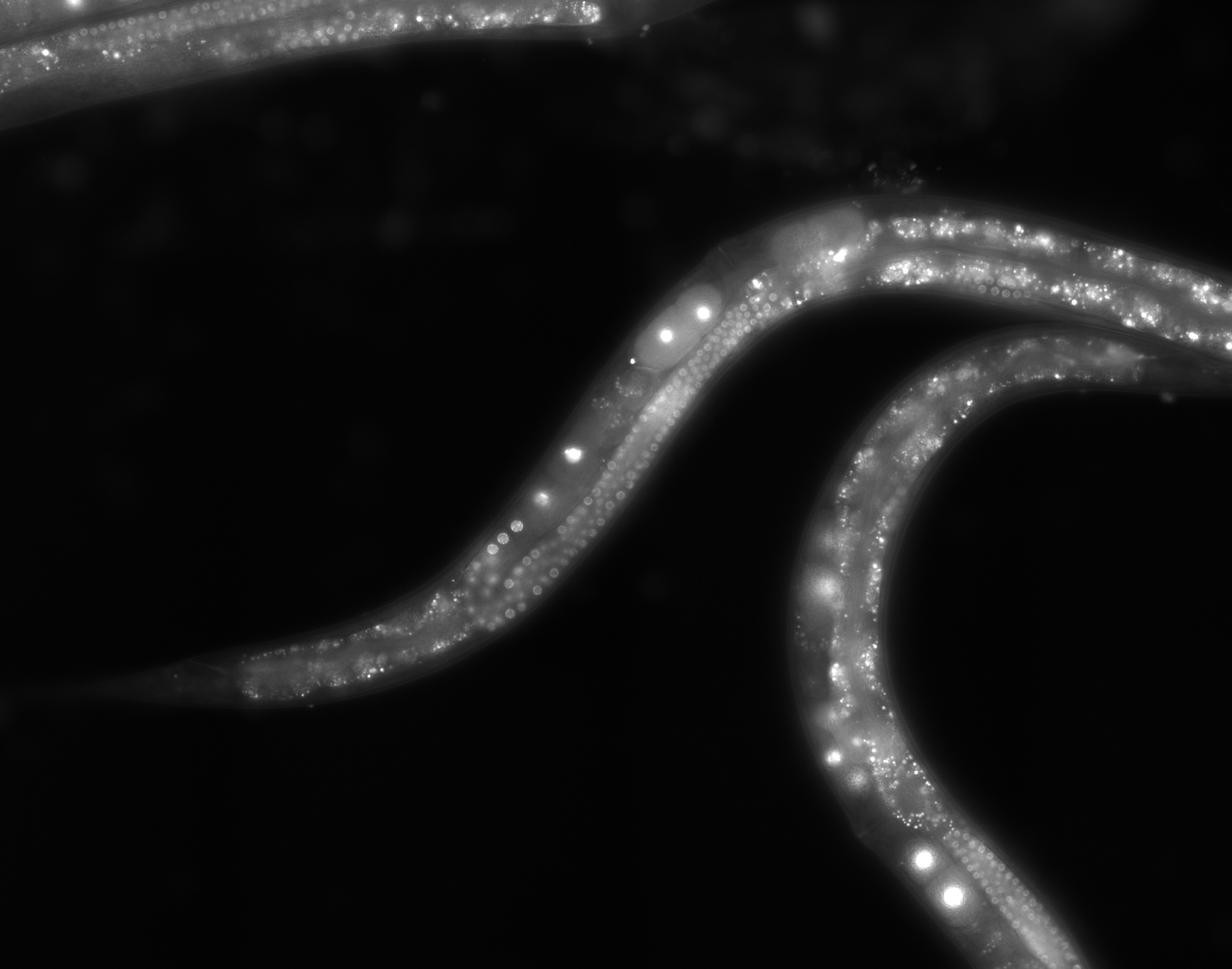

Supplement: Supplemental Material [file supp_gad.322446.118_Supplemental_Source_material.zip › Fig 4 Source material/Panel A/prde-1.tif]

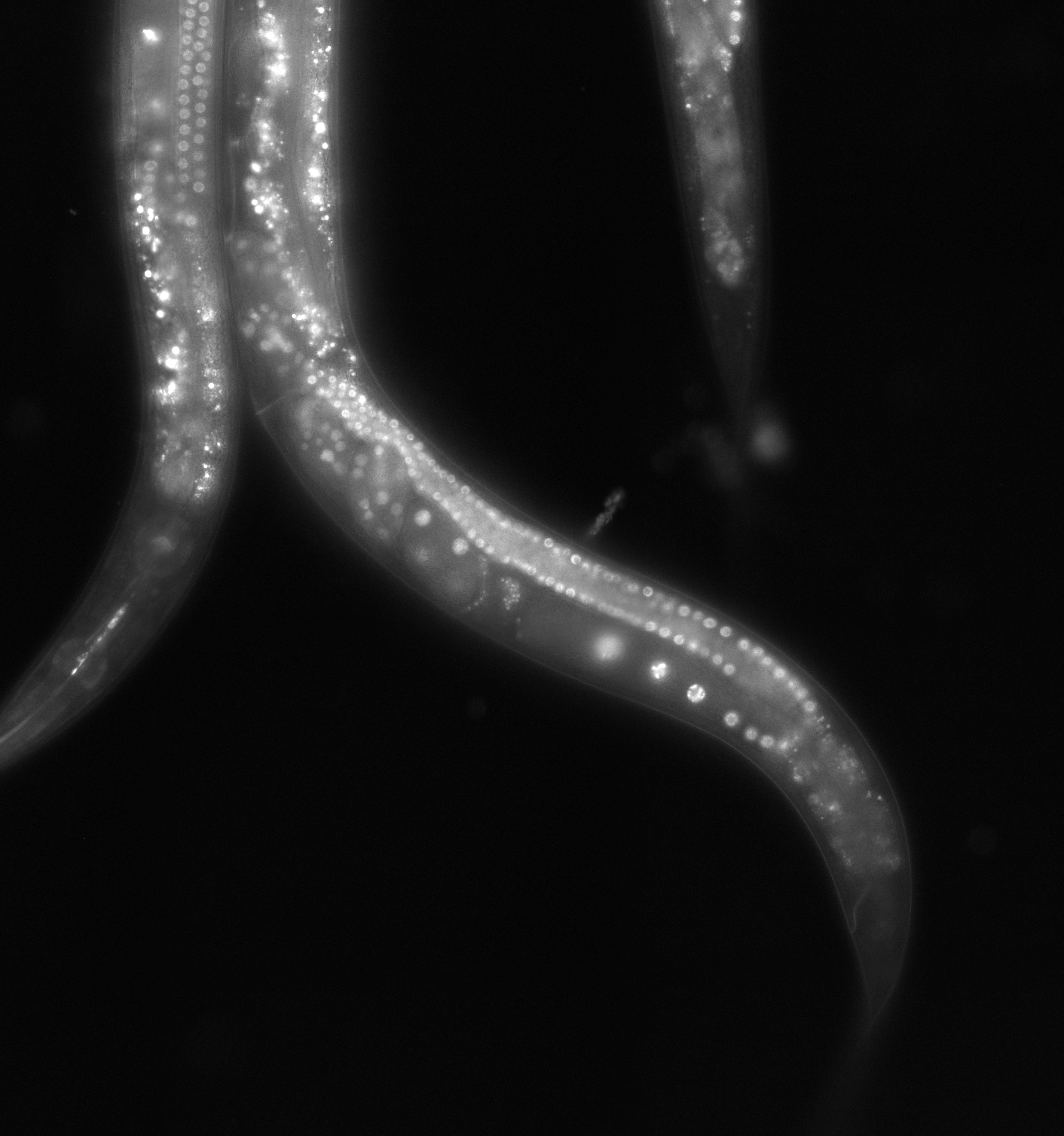

Supplement: Supplemental Material [file supp_gad.322446.118_Supplemental_Source_material.zip › Fig 4 Source material/Panel A/tofu-6.tif]

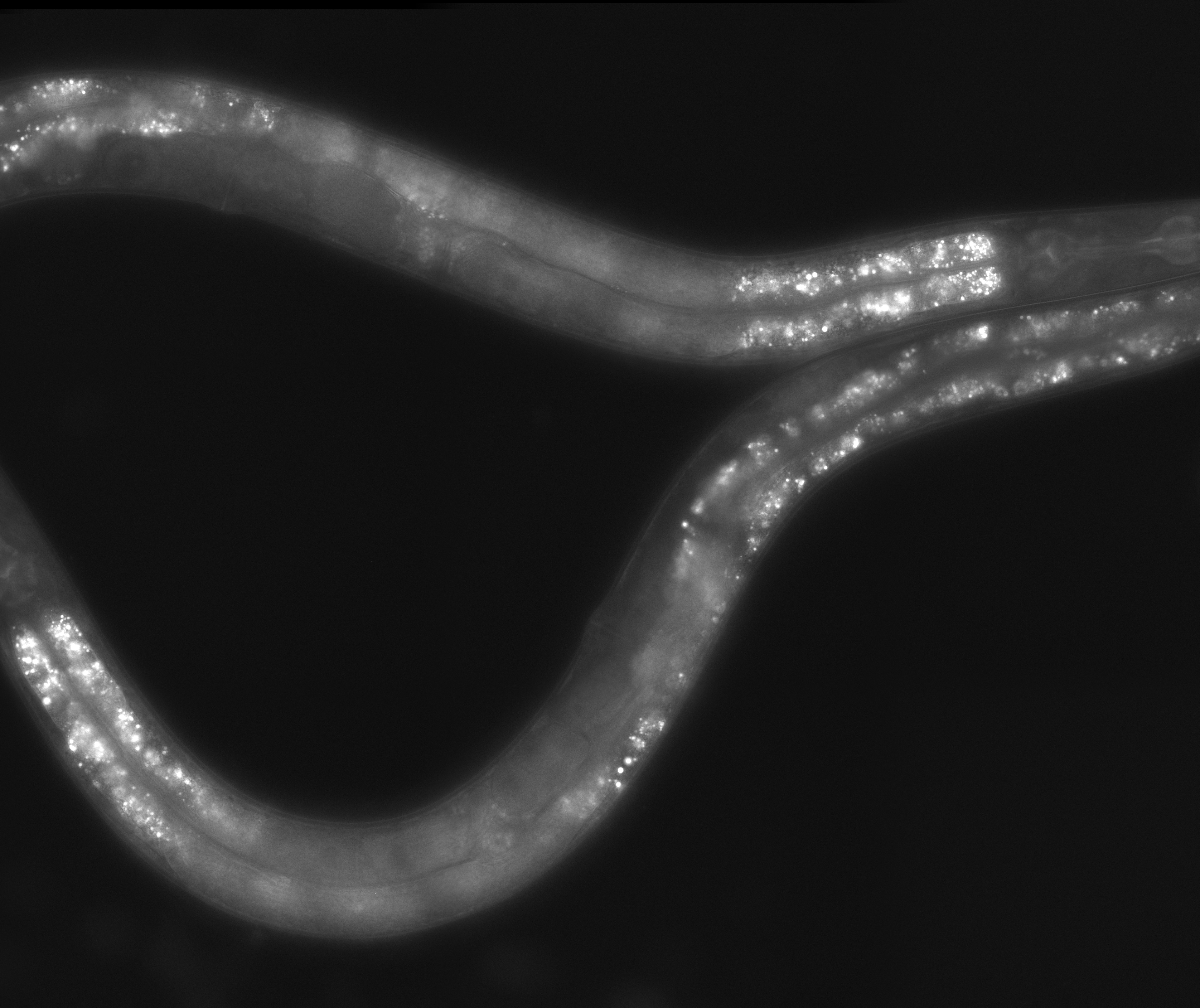

Supplement: Supplemental Material [file supp_gad.322446.118_Supplemental_Source_material.zip › Fig 4 Source material/Panel A/tost-1.tif]
